# Scandium-catalysed intermolecular hydroaminoalkylation of olefins with aliphatic tertiary amines†
†Electronic supplementary information (ESI) available: Full experimental details for catalytic procedures and characterization data. See DOI: 10.1039/c6sc02129h



**DOI:** 10.1039/c6sc02129h

**Published:** 2016-07-04

**Authors:** Adi E. Nako, Juzo Oyamada, Masayoshi Nishiura, Zhaomin Hou

**Affiliations:** a Organometallic Chemistry Laboratory and RIKEN Center for Sustainable Resource Science , RIKEN , 2-1 Hirosawa, Wako , Saitama , 351-0198 , Japan . Email: houz@riken.jp

## Abstract

A homoleptic scandium trialkyl complex in combination with a borate compound served as an excellent catalyst for the C–H addition of aliphatic tertiary amines to olefins. This highly regiospecific, 100% atom efficient C–H bond alkylation reaction was applicable to a wide variety of tertiary amines and olefins, including functionalised styrenes and unactivated α-olefins. This work represents the first example of rare-earth catalysed olefin hydroaminoalkylation and also the first example of catalytic C–H addition of aliphatic tertiary amines to olefins with any catalyst.

## Abstract 2

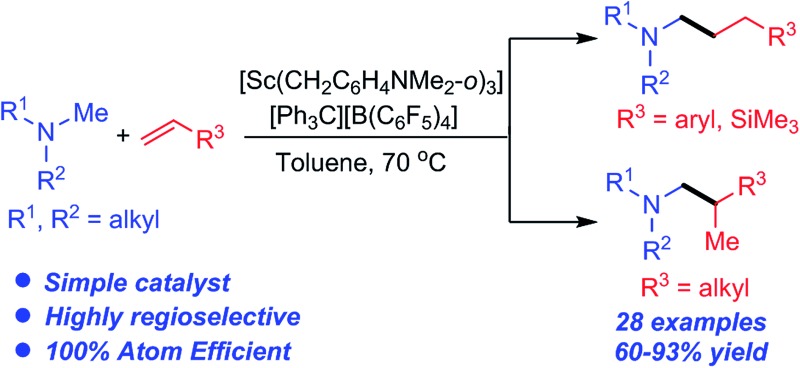
A homoleptic scandium trialkyl complex in combination with a borate compound served as an excellent catalyst for the C–H addition of aliphatic tertiary amines to various olefins.

## Introduction

Aliphatic tertiary amines are ubiquitous across the chemical sciences. Therefore, the synthesis of tertiary amines bearing diverse substituents in a selective and atom efficient manner is of great interest and significance. In this regard, the catalytic hydroamination,^1^ hydroaminomethylation^2^ and reductive amination^3^ of unsaturated bonds can serve as useful synthetic routes to these molecules. However, all of these transformations require cleavage of an N–H bond to form a C–N bond, thus limiting potential substrates to primary and secondary amines.^4^

**Scheme 1:**
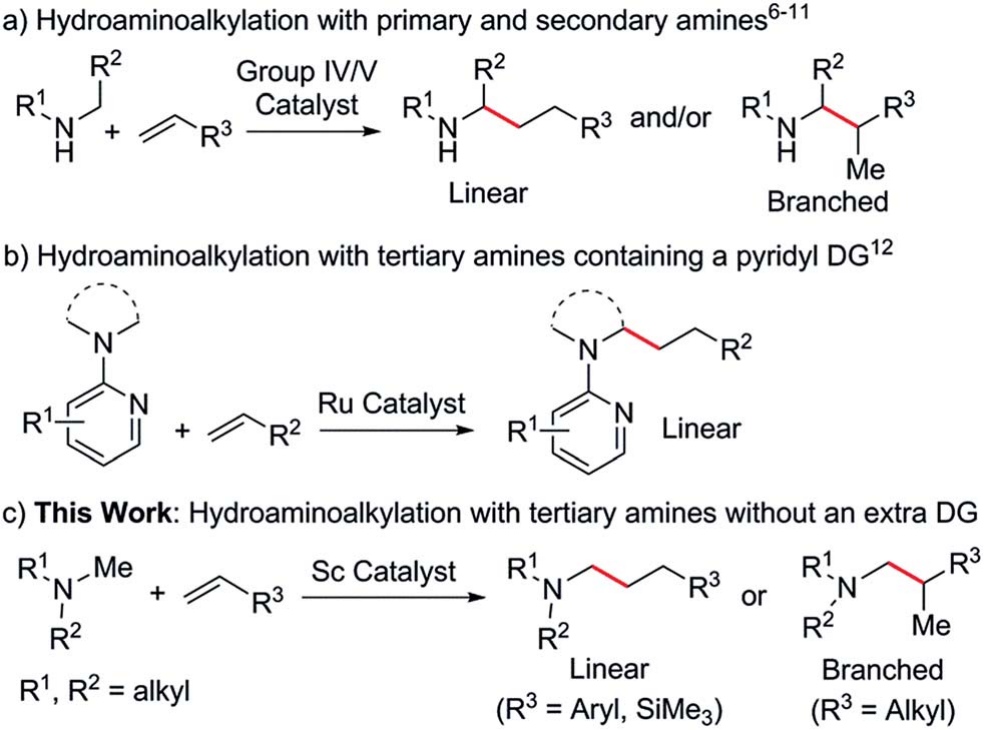
Hydroaminoalkylation of olefins using different amines and different catalysts.

Since the discovery that under certain conditions, group IV and V metal catalysts gave C–C bond formation products instead of the expected C–N coupled products,^5^ intermolecular hydroaminoalkylation has been rapidly gaining interest as a 100% atom efficient synthetic route to substituted amines.^6^ It has been shown that *N*,*N*-^7^ and *N*,*O*-chelated early transition metal complexes based on 2-pyridonate,^8^ amidate,^9^ phosphoramidate^10^ and related ligands are active catalysts for the hydroaminoalkylation of olefins with a variety of primary and secondary amines (Scheme 1a). The reactions are widely considered to occur *via* the initial formation of metal amido intermediates through deprotonation of an N–H bond,^11^ therefore limiting the starting materials to primary and secondary amines. As the N–H bond is restored at the end of the reaction, the corresponding α-C-alkylated primary or secondary amines are obtained as the final products.

Ruthenium catalysts have been reported to mediate intermolecular hydroaminoalkylation with tertiary amines, but in all cases a pyridyl directing group (DG) was essential for reactivity (Scheme 1b).^12^ Catalytic hydroaminoalkylation of olefins with tertiary amines that do not contain an extra directing group has not been reported previously. This is most likely due to a lack of suitable catalysts which can not only effectively interact with an unactivated tertiary amine moiety but also promote C–H activation and C=C double bond insertion. The search for new catalysts for the hydroaminoalkylation of olefins with aliphatic tertiary amines is therefore of considerable importance.

We have recently shown that half sandwich mono-cationic rare-earth alkyl species act as efficient catalysts for various transformations, including olefin polymerization,^13^ the alkylation of sp^2^ and sp^3^ C–H bonds of aromatic compounds such as anisoles, pyridines and *N*,*N*-dimethylanilines,^14^ and the C–H polyaddition of 1,4-dimethoxybenzene to unconjugated dienes.^15^ These C–H functionalisation reactions were all achieved by the initial interaction between a rare-earth metal ion and a substrate heteroatom (such as O and N) followed by deprotonation of an *ortho* C–H bond with a metal alkyl species and the subsequent insertion of a C=C double bond into the resulting M–C bond. This unique catalytic transformation could be ascribed to the strong heteroatom affinity of the rare-earth metal ions as well as to the high activity of the cationic rare-earth metal alkyl species towards both C–H activation and C=C double bond insertion. These results encouraged us to examine whether rare-earth catalysts could promote the hydroaminoalkylation of olefins with tertiary amines. Herein we report the first intermolecular hydroaminoalkylation of various olefins with aliphatic tertiary amines using a cationic scandium alkyl catalyst (Scheme 1c).

## Results and discussion

### Catalyst screening

At first we examined the half-sandwich scandium dialkyl complex [(C_5_Me_5_)Sc(CH_2_C_6_H_4_NMe_2_-*o*)_2_]^16*a*^ together with one equivalent of [Ph_3_C][B(C_6_F_5_)_4_] for the catalytic C–H addition of *N*,*N*-dimethylbutylamine (**1a**) to norbornene (**2a**) (Table 1, entry 1). Whilst this reaction was found to give the corresponding hydroaminoalkylation product **3a** quantitatively after 24 h at 70 °C, initial attempts to expand the substrate scope to include styrene and 1-hexene were unsuccessful (Table 1, entries 3 and 5). In contrast, when the Cp-free trialkyl compound [Sc(CH_2_C_6_H_4_NMe_2_-*o*)_3_]^16*b*^ was used instead, significant product formation was observed for both styrene and 1-hexene without a noticeable drop in catalytic activity for norbornene (Table 1, entries 2, 4 and 6).^17^ These reactions gave the corresponding methyl-functionalised products **3a**, **4a′** and **5f′** with no other functionalised products or regioisomers observed by ^1^H NMR. A significant metal effect was observed for the hydroaminoalkylation of **2a** to give **3a**. When [Ln(CH_2_C_6_H_4_NMe_2_-*o*)_3_] (Ln = Y, Lu, Gd, Sm) was used as a pre-catalyst with one equivalent of [Ph_3_C][B(C_6_F_5_)_4_], no reaction was observed, highlighting the importance of a small, highly electropositive metal centre in the activation of tertiary amine methyl groups (Table S1,† entries 3–6). No reaction was seen when either [Sc(CH_2_C_6_H_4_NMe_2_-*o*)_3_] or [Ph_3_C][B(C_6_F_5_)_4_] alone was used as a catalyst, further highlighting the importance of a cationic scandium alkyl species in this reaction (Table S1,† entries 7 and 8).^18^ When two equivalents of borate were used in combination with [Sc(CH_2_C_6_H_4_NMe_2_-*o*)_3_], only trace reactivity was observed (Table S1,† entry 9).

**Table 1:** A comparison of [(C_5_Me_5_)Sc(CH_2_C_6_H_4_NMe_2_-*o*)_2_] and [Sc(CH_2_C_6_H_4_NMe_2_-*o*)_3_] as catalysts

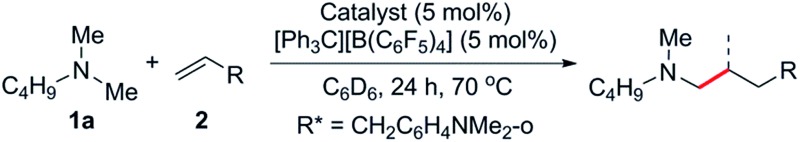
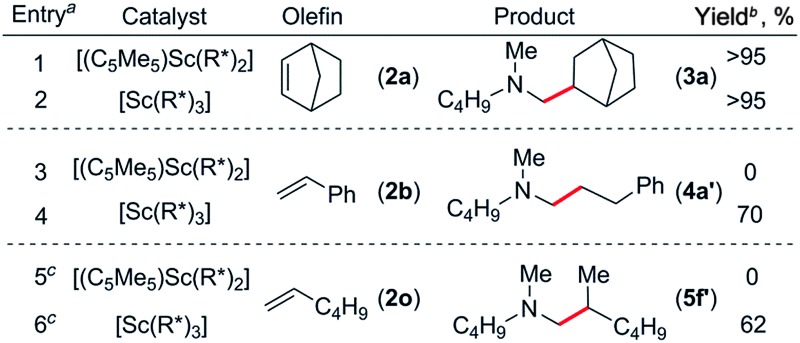

^*a*^Reactions were carried out with 0.25 mmol amine and 0.275 mmol norbornene in 1 mL of C_6_D_6_.

^*b*^NMR yield calculated against Cp_2_Fe as an internal standard.

^*c*^10 mol% of co-catalysts used.

### Hydroaminoalkylation of norbornene with various aliphatic amines

Based on these catalyst screening results, the [Sc(CH_2_C_6_H_4_NMe_2_-*o*)_3_]/[Ph_3_C][B(C_6_F_5_)_4_] combination was then chosen as a catalyst to investigate the C–H addition of various tertiary amines to norbornene. Some representative results are summarised in Table 2. Simple dimethylamine substrates such as *N*,*N*-dimethylbutylamine (**1a**), *N*,*N*-dimethyloctylamine (**1b**) and *N*,*N*-dimethylcyclohexylamine (**1c**) are efficient for this reaction, giving the corresponding *N*-methyl-alkylation products **3a–c** in 87, 91 and 93% isolated yields, respectively (Table 2, entries 1–3). Acyclic monomethylamines such as *N*-methyl-*N*-ethylbutylamine (**1d**) and *N*-methyldibutylamine (**1e**) can also be used to give the corresponding products **3d** and **3e** in 88 and 86% yields, respectively (Table 2, entries 4 and 5).

**Table 2:** Catalytic C–H addition of various tertiary aliphatic amines to norbornene

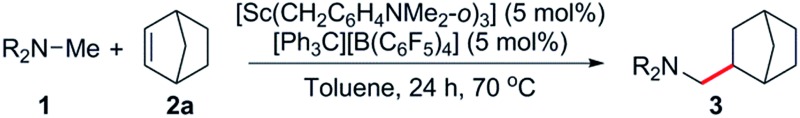
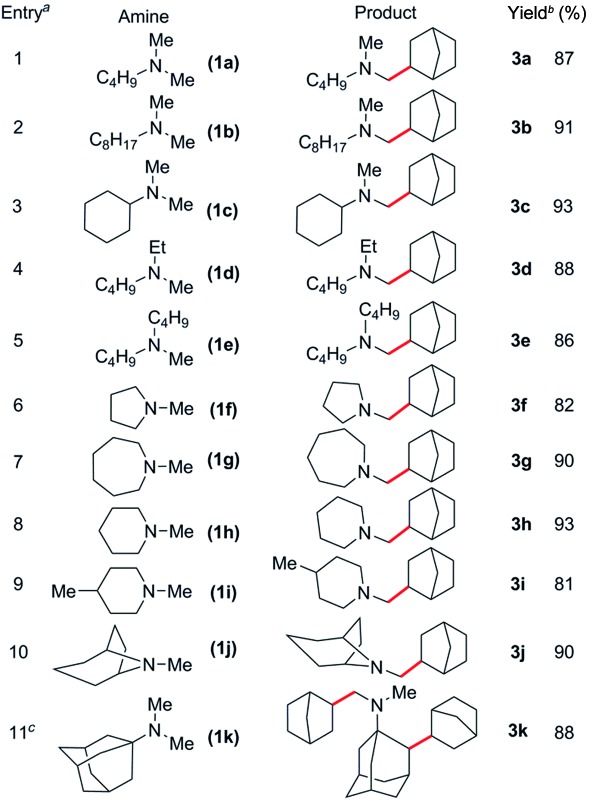

^*a*^Reactions were carried out with 1 mmol amine and 1.1 mmol norbornene in 4 mL of toluene unless otherwise stated.

^*b*^Isolated yield.

^*c*^4 mmol norbornene used.

The reaction scope was also expanded to include cyclic methylamines, including the pharmacologically important tropane (**1j**), to give the products **3f–j** in 81 to 93% yields (Table 2, entries 6–10). When *N*,*N*-dimethyladamantylamine (**1k**) was used as a substrate, clean conversion to the dialkylated species **3k** was observed in the presence of excess olefin (4 equiv., Table 2, entry 11). Remarkably, subsequent alkylation was found to occur at the secondary β-C–H bond of the adamantyl moiety.^19^ This could be a result of intramolecular C–H activation of the β-C–H bond following initial alkylation at one amine methyl group (Scheme S1†). Attempts to expand the scope to include sterically congested amines such as *N*,*N*-dicyclohexylamine failed, presumably due to steric hindrance, whereas *N*,*N*-diethylmethylamine also gave no reaction, most likely due to strong coordination resulting from a lack of steric bulk.^14*c*^

### Hydroaminoalkylation of various olefins with *N*-methylpiperidine

Based on these results, *N*-methylpiperidine (**1h**) was chosen to explore the olefin scope of this reaction and some representative results are summarised in Tables 3 and 4. The catalyst system [Sc(CH_2_C_6_H_4_NMe_2_-*o*)_3_]/[Ph_3_C][B(C_6_F_5_)_4_] was found to be very effective for the C–H addition of **1h** to styrene (**2b**), giving exclusively the linear product **4a** in 85% yield (Table 3, entry 1). Whilst this regioselectivity is common for related rare-earth catalysed C–H additions to styrenes,^14^ early transition metal catalysts regularly give branched,^5g,7c,7e,11a^ linear^7*b*^ or often mixtures of both isomers.^6–11^ Alkyl-substituted styrene derivatives such as **2c** and **2d** were also tolerated (Table 3, entries 2 and 3). In the case of the vinylaniline **2e**, at a 1 : 1.1 ratio of **1h** to **2e** the polymerization of **2e** was found to be competitive with hydroaminoalkylation.^20^ However, when an excess amount of **1h** (5 equiv.) was used, the hydroaminoalkylation product **4d** was obtained exclusively (89% yield, Table 3, entry 4).^21^ No alkylation was observed at the aniline methyl groups,^22^ and there was no evidence for *ortho*-alkylation of the aromatic ring.^14*g*^ Styrene derivatives with *para*-electron-withdrawing groups such as fluoro, chloro and phenyl were also suitable for this reaction, though an increased catalyst loading was needed to obtain high yields (Table 3, entries 5–7). Oxygen containing substrates such as 4-methoxystyrene and 4-*tert*-butoxystyrene were incompatible with this catalyst system, likely due to the substantial oxophilicity of the sterically flexible cationic scandium centre. In addition to styrenes, vinyltrimethylsilane (**2i**) also gave the linear product **4h** in 83% yield (Table 3, entry 8).^23^

**Table 3:** Catalytic C–H addition of *N*-methylpiperidine to various olefins to give linear products

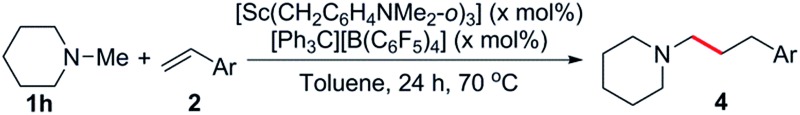
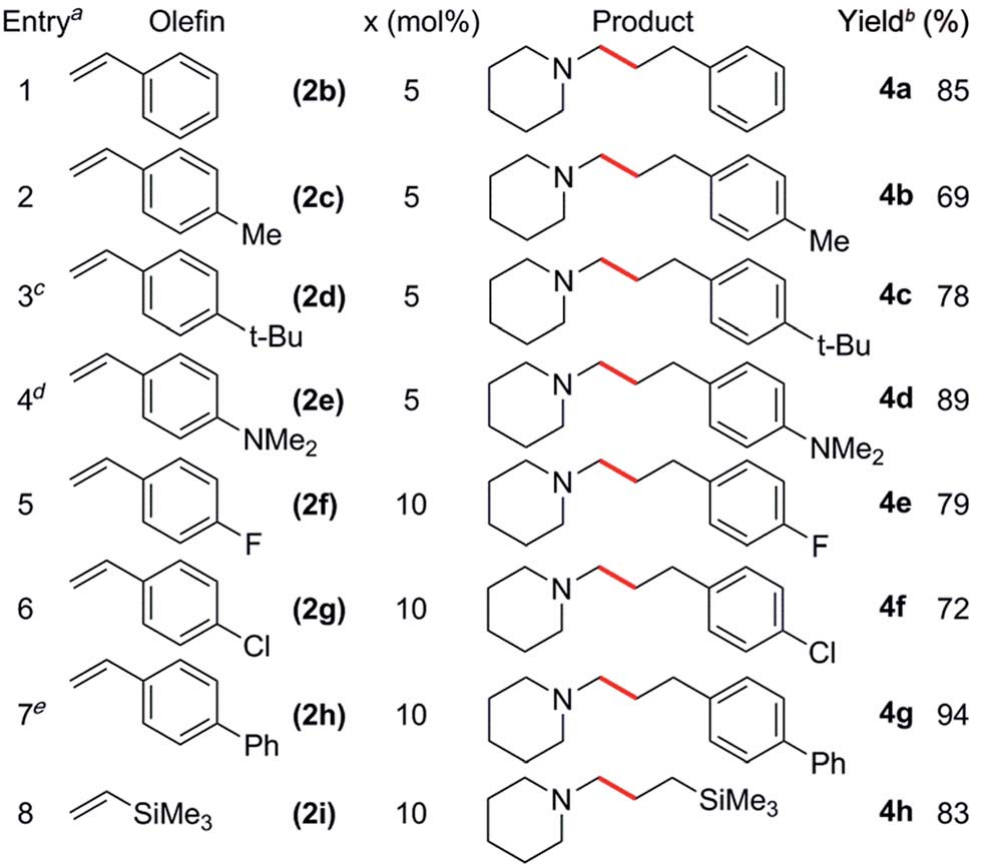

^*a*^Reactions were carried out with 1 mmol *N*-methylpiperidine and 1.1 mmol olefin in 4 mL of toluene unless otherwise stated.

^*b*^Isolated yield.

^*c*^48 h reaction time.

^*d*^5 mmol amine.

^*e*^36 h reaction time.

**Table 4:** Catalytic C–H addition of *N*-methylpiperidine to various olefins to give branched products

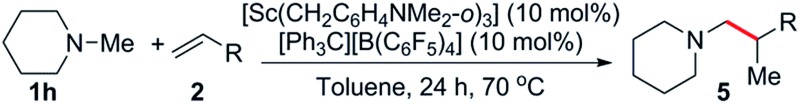
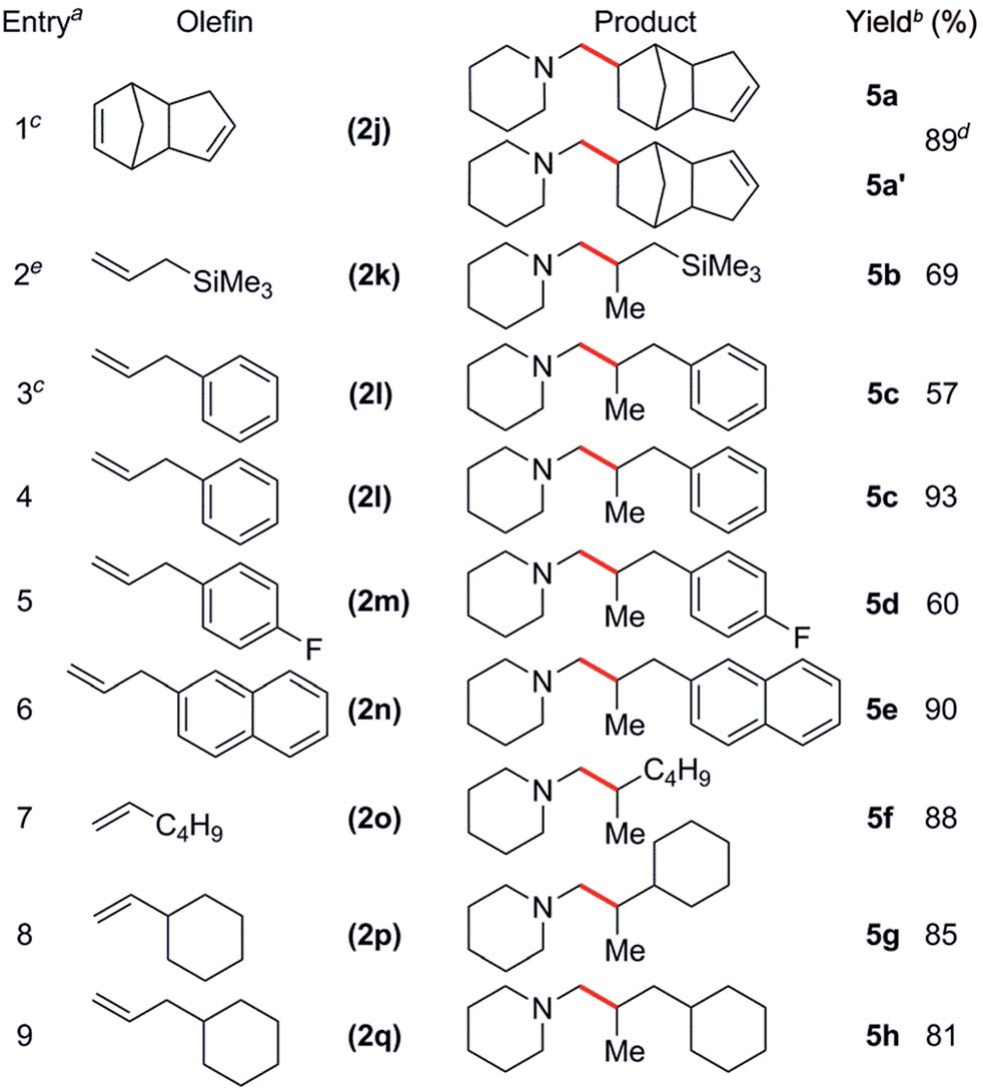

^*a*^Reactions were carried out with 1 mmol *N*-methylpiperidine and 1.1 mmol olefin in 4 mL of toluene unless otherwise stated.

^*b*^Isolated yield.

^*c*^5 mol% of co-catalysts used.

^*d*^1 : 3 mixture of regioisomers.

^*e*^48 h reaction time.

The reaction scope was also expanded to include alkyl substituted olefins, which gave exclusively branched products (Table 4). This is in sharp contrast to late transition metal systems which give linear alkylation products for both alkyl and aryl substituted olefins.^12^ When dicyclopentadiene (DCPD) was used as a substrate, the reaction occurred exclusively at the norbornene fragment, and a mixture of regioisomers was isolated in a *ca.* 1 : 3 **5a** : **5a′** ratio in 89% yield (Table 4, entry 1); similar results have previously been observed in the polymerisation^24^ and hydropyridinylation^14*c*^ of DCPD. A variety of allylic substrates were also tolerated, with allyltrimethylsilane giving **5b** in 69% yield (Table 4, entry 2). When a 5 mol% catalyst loading was used for the reaction of **1h** with allylbenzene (**2l**), the corresponding branched product (**5c**) was obtained in 57% yield and this could be improved to 93% yield by increasing the catalyst loading (Table 4, entries 3 and 4). The addition of a *para*-fluoro group had a significant effect on the yield of **5d** (60%, Table 4, entry 5), whereas reaction with allylnaphthalene gave **5e** in 90% yield. Unactivated, simple α-olefins such as 1-hexene (**2o**), vinylcyclohexene (**2p**) and allylcyclohexene (**2q**) all gave the corresponding products **5f**, **5g** and **5h** in high yields (81–88%) despite the reactions being performed with a 1 : 1.1 amine : olefin ratio (Table 4, entries 7–9). This is in contrast to the alkylation of pyridines by Cp-supported rare-earth catalysts that often require a significant excess of olefin (10–30 equiv.) to give high yields.^14^ Attempts to expand the substrate scope to include ethylene and conjugated dienes such as isoprene and 1,3-cyclohexadiene resulted in the polymerization of the olefin monomers,^25^ with no consumption of *N*-methylpiperidine observed by ^1^H NMR spectroscopy. Attempts to favour hydroaminoalkylation by using an excess of amine (5 equiv., *vide supra*) failed for both the aforementioned dienes.

### Possible mechanism of C–H functionalisation including isotopic labelling studies

The reaction of a 1 : 1 : 1 mixture of *d*_3_-*N*-methylpyrrolidine, *N*-methylpyrrolidine and styrene showed a significant kinetic isotope effect (*k*_H_/*k*_D_ = 2.70) (eqn (1), Fig. S1†). A kinetic isotope effect was also observed when the initial rates of the separate C–H (eqn (2)) and C–D (eqn (3)) addition reactions were compared (*k*_H_/*k*_D_ = 1.97), implying that C–H activation could be involved in the rate determining step in this reaction (Fig. S2†).
1

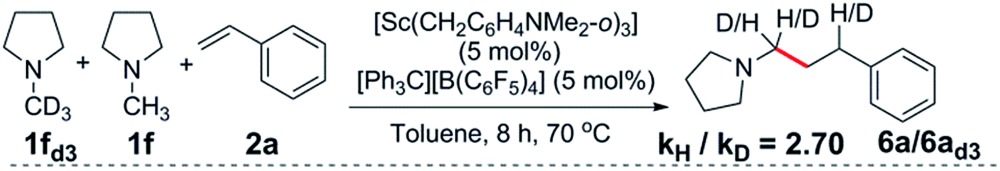



2

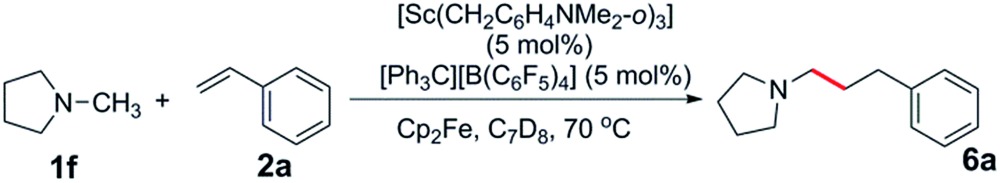



3

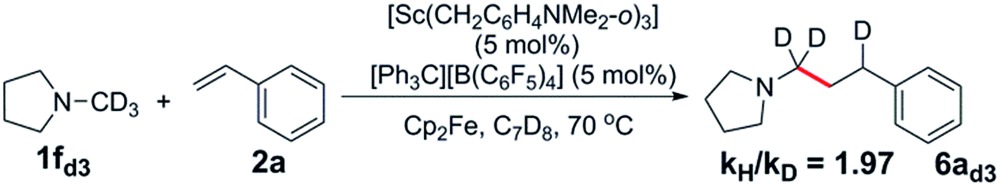




**Scheme 2:**
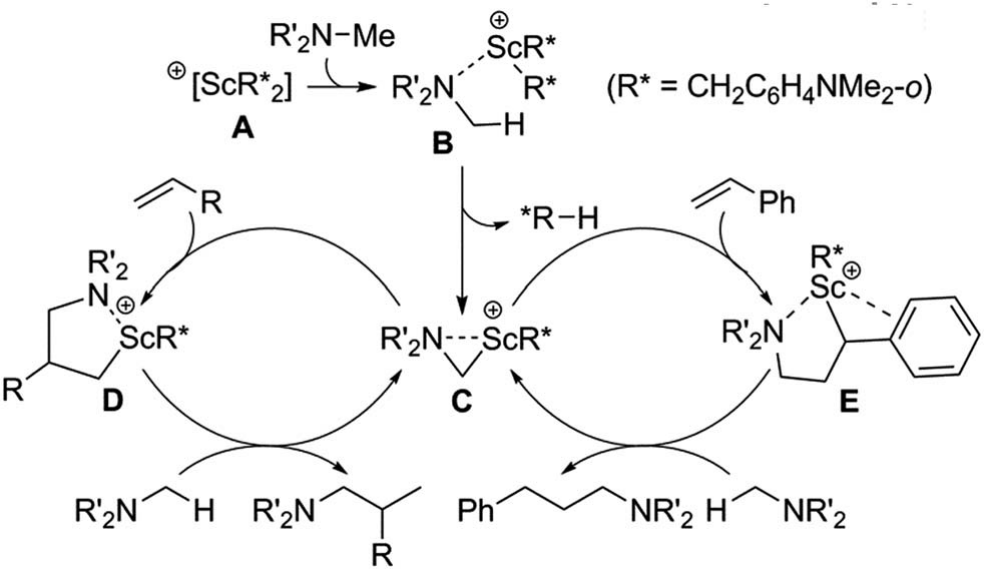
Plausible mechanisms of scandium-catalysed intermolecular hydroaminoalkylation of both α-olefins and styrenes.

A plausible catalytic mechanism is shown in Scheme 2. The reaction of [Sc(CH_2_C_6_H_4_NMe_2_-*o*)_3_] with an equiv of [Ph_3_C][B(C_6_F_5_)_4_] would afford a mono-cationic scandium bis(aminobenzyl) species **A**, which upon coordination of the amine NMe_2_ group would form an adduct such as **B**.^14*g*^ Deprotonation of an amine methyl group by an aminobenzyl ligand (R*) would then give an η^2^-azametallacyclic intermediate, **C**, along with release of one equivalent of *N*,*N*-dimethyl-*o*-toluidine. This highly strained species would then undergo 1,2-addition to an α-olefin to give the corresponding branched ring-expanded azametallocyclic intermediate, **D**. Subsequent C–H activation of another molecule of amine substrate would release the corresponding branched product and complete the catalytic cycle. When styrene derivatives are used as substrates, 2,1-insertion instead occurs, possibly due to stabilization of the Sc centre through interaction with the aromatic ring of **E**, which undergoes subsequent activation of a further amine equivalent to release the corresponding linear product.^14^

## Conclusion

In summary, we have achieved the efficient and highly selective catalytic hydroaminoalkylation of various olefins with tertiary amines by using a homoleptic Sc alkyl pre-catalyst. This represents not only the first example of rare-earth catalysed olefin hydroaminoalkylation but also the first example of 100% atom efficient, intermolecular catalytic C–H alkylation of aliphatic tertiary amines with any catalyst. The reaction occurs between a wide variety of cyclic and acyclic amines and olefins, including functionalised styrenes and unactivated α-olefins, affording a new family of tertiary amines with diversified substituents. The C–H alkylation of a β-C–H bond was also observed, demonstrating that this chemistry may be extended to amine-directed functionalisation of remote C–H bonds. The success of these transformations is obviously due to the unique affinity and reactivity of cationic scandium alkyl species towards tertiary amino groups and C–H and C=C bonds. Moreover, the observation that a homoleptic trialkyl scandium complex such as [Sc(CH_2_C_6_H_4_NMe_2_-*o*)_3_] can show superior performance to its Cp-ligated analogue [(C_5_Me_5_)Sc(CH_2_C_6_H_4_NMe_2_-*o*)_2_] for C–H functionalisation is also remarkable. These results suggest that cationic homoleptic rare earth alkyls, which have previously received little attention as catalysts for organic synthesis, may activate and functionalise C–H bonds that do not work with other catalysts. Further studies along this direction are currently in progress.
