# Supplementary material for: Scandium-catalysed intermolecular hydroaminoalkylation of olefins with aliphatic tertiary amines
Source: Chem Sci. 2016 Jul 4;7(10):6429–34. doi: 10.1039/c6sc02129h (PMC5356020; doi:10.1039/c6sc02129h)
Supplement: Supplementary file 1 [file SC-007-C6SC02129H-s001.pdf]

Supporting Information

Scandium-Catalyzed Intermolecular Hydroaminoalkylation of Olefins with Aliphatic Tertiary Amines

Adi E. Nako, Juzo Oyamada, Masayoshi Nishiura and Zhaomin Hou*

Organometallic Chemistry Laboratory and RIKEN Center for Sustainable Resource Science, RIKEN, 2-1 Hirosawa, Wako, Saitama 351-0198, Japan

houz@riken.jp

Contents

1. General Methods **S2**

2. Catalyst Screening **S3**

3. Typical Procedure for the Catalytic Alkylation of Amines with Olefins **S4**

4. Analytical Data **S4**

5. Kinetic Isotope Effect Experiments **S9**

6. Dialkylation of 1k to give 3k **S11**

7. References **S12**

8. ^{1}H and ^{13}C NMR Spectra of Products **S13**

1. General Methods

All manipulations were performed under a nitrogen atmosphere by use of standard Schlenk techniques or in an mBRAUN Labmaster glovebox. Nitrogen was purified by passing through a Dryclean column (4 Å molecular sieves, Nikka Seiko Co.) and a Gasclean GC-XR column (Nikka Seiko Co.). Hexane, THF, toluene and benzene (dehydrated, stabilizer-free) were obtained from Kanto Chemical Co. and purified by use of an MBraun SPS-800 solvent purification system. Silica gel column chromatography was performed with Silica Gel 60 N (spherical, neutral, 40-50 mm) obtained from Kanto Chemical Co. [Ln($CH_2C_6H_4NMe_2$-o)$_3$] (Ln = Sc, Y, Lu, Gd, Sm)[1] and [(C_5Me_5)Sc($CH_2C_6H_4NMe_2$-o)$_2$][2] were prepared according to the literature methods. *N,N*-dimethylbutylamine (**1a**), *N,N*-dimethyloctylamine (**1b**), *N,N*-dimethylcyclohexylamine (**1c**), *N,N*-dibutylmethylamine (**1e**), *N*-methylpyrrolidine (**1f**), d_3-*N*-methylpyrrolidine (**1f$_{d3}$**), *N*-methylpiperidine (**1h**) and tropane (**1j**) were commercially available. *N*-methyl,*N*-ethylbutylamine (**1d**), *N*-methyl,4-methylpiperidine (**1i**), *N*-methylhexamethyleneimine (**1g**), and *N,N*-dimethyladamantylamine (**1k**), were prepared *via* the literature method.[3] All olefins were commercially available, except 4-dimethylaminostyrene (**2e**) which was synthesized *via* the literature method.[4] Amines and olefins were all distilled from appropriate drying agents such as CaH_2 and Na. All ^{1}H NMR and ^{13}C NMR spectra were recorded on either a JEOL AL-400 MHz instrument or a Bruker AVANCE III HD 500 NMR spectrometer in C_6D_6 with tetramethylsilane as an internal standard otherwise mentioned. Data are reported as follows: chemical shift in ppm (d), multiplicity (s = singlet, d = doublet, t = triplet, q = quartet, quin = quintet, sex = sextet, sep = septet, m = multiplet, br = broad signal), coupling constant (Hz), integration. Gas chromatography analysis was performed on Shimadzu GC2014 using a capillary column (Agilent J&W GC columns DB-1, 30m, 0.32 mm i.d., 0.25 mm film thickness. High-resolution MS were obtained on a Bruker microTOF-Q III (ESI+).

2. Catalyst Screening

In a glovebox, [Sc($CH_2C_6H_4NMe_2$-*o*)$_3$] (6 mg, 0.013 mmol) was dissolved in C_6D_6 (1.0 mL). To this solution, ferrocene (19 mg, 0.1 mmol), *N,N*-dimethylbutylamine (25mg, 0.25 mmol), norbornene (26 mg, 0.28 mmol) and [Ph_3C][$B(C_6F_5)_4$] (16 mg, 0.013 mmol) were added sequentially. The biphasic mixture was transferred to a J Young NMR tube, sealed and a baseline ^{1}H NMR spectrum taken. The tube was then heated at 70 °C for 24 hours, and the yield calculated from the internal standard (ferrocene). For entries 5 and 6, the sample was quenched with EtOAc (2 mL) after 24 h at 70 °C, filtered and the volatiles removed *in vacuo* to remove the paramagnetic catalyst.

Table S1. Catalyst Dependant Hydroaminoalkylation of Norbornene with N,N-dimethylbutylamine

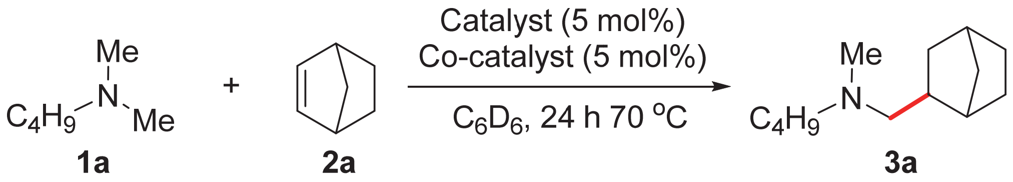

Entry[a]	Catalyst	Co-catalyst	Yield, %[b]
1	[($C_5Me_4SiMe_3$)Sc($CH_2C_6H_4NMe_2$-o)$_2$]	[Ph_3C][$B(C_6F_5)_4$]	90
2	[(C_5H_5)Sc($CH_2C_6H_4NMe_2$-o)$_2$]	[Ph_3C][$B(C_6F_5)_4$]	52
3	[Y($CH_2C_6H_4NMe_2$-o)$_3$]	[Ph_3C][$B(C_6F_5)_4$]	0
4	[Lu($CH_2C_6H_4NMe_2$-o)$_3$]	[Ph_3C][$B(C_6F_5)_4$]	0
5	[Gd($CH_2C_6H_4NMe_2$-o)$_3$]	[Ph_3C][$B(C_6F_5)_4$]	0
6	[Sm($CH_2C_6H_4NMe_2$-o)$_3$]	[Ph_3C][$B(C_6F_5)_4$]	0
7	[Sc($CH_2C_6H_4NMe_2$-o)$_3$]	N/A	0
8	N/A	[Ph_3C][$B(C_6F_5)_4$]	0
9[c]	[Sc($CH_2C_6H_4NMe_2$-o)$_3$]	[Ph_3C][$B(C_6F_5)_4$]	<5

[a] Reactions were carried out with 0.25 mmol amine and 0.275 mmol norbornene in 1 mL of C_6D_6. [b] NMR yield calculated against Cp_2Fe as an internal standard. [c] 10 mol% [Ph_3C][$B(C_6F_5)_4$] used.

3. Typical Procedure for the Catalytic Alkylation of Amines with Olefins

In a glovebox, [Sc($CH_2C_6H_4NMe_2$-*o*)$_3$] (22 mg, 0.05 mmol) was dissolved in toluene (2.0 mL). To this solution *N*-methylpiperidine (99 mg, 1 mmol), styrene (115 mg, 1.1 mmol) and [Ph_3C][B(C_6F_5)$_4$] (46 mg, 0.05 mmol) were added sequentially. The biphasic mixture was transferred to a Schlenk ampoule, sealed and heated at 70 ^{o}C for 24 hours. EtOAc (5 mL) was added to the crude mixture and the volatiles removed *in vacuo.* The compound was purified by silica gel column chromatography (hexane/EtOAc), to afford **4a** as colourless oil (173 mg, 0.851 mmol, 85 % yield).

4. Analytical Data

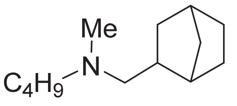

3a: ^{1}H NMR (500 MHz, C_6D_6): δ 2.30 – 2.25 (m, 2H), 2.22 (br, 1H), 2.16 (br, 1H), 2.16 – 2.12 (m, 1H), 2.14 (s, 3H), 1.94 (dd, *J* = 7.5 Hz, 4.5 Hz, 1H), 1.64 – 1.58 (m, 1H), 1.52 – 1.40 (m, 4H), 1.37 – 1.27 (m, 4H), 1.18 – 1.13 (m, 1H), 1.05 (d, *J* = 9.5 Hz, 1H), 0.91 (t, *J* = 7.0 Hz, 3H). ^{13}C NMR (125 MHz, C_6D_6): δ 64.3, 58.3, 42.7, 40.7, 39.9, 36.9, 36.5, 35.6, 30.5, 30.2, 29.5, 20.9, 14.3. HR MS (ESI+): Found 196.2066 [M+H]$^+$, calcd. for $C_{13}H_{26}N^+$ 196.2065.

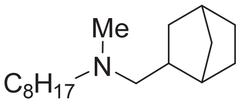

3b: ^{1}H NMR (500 MHz, C_6D_6): δ 2.32 – 2.27 (m, 2H), 2.23 (br, 1H), 2.18 – 2.14 (m, 2H), 2.16 (s, 3H), 1.96 (dd, *J* = 7.0 Hz, 5.0 Hz, 1H), 1.66 – 1.60 (m, 1H), 1.50 – 1.45 (m, 4H), 1.38 – 1.25 (br m, 12H), 1.18 – 1.13 (m, 3H), 1.05 (d, *J* = 9.5 Hz, 1H), 0.90 (t, *J* = 8.0 Hz, 3H). ^{13}C NMR (125 MHz, C_6D_6): δ 64.3, 58.7, 42.7, 40.7, 39.9, 37.0, 36.5, 35.6, 32.4, 30.5, 30.1, 29.9, 29.5, 28.1, 27.9, 23.1, 14.4. HR MS (ESI+): Found 252.2692 [M+H]$^+$, calcd. for $C_{17}H_{34}N^+$ 252.2691.

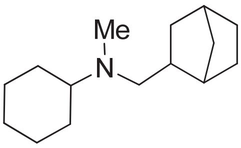

3c: ^{1}H NMR (500 MHz, C_6D_6): δ 2.35 – 2.17 (m, 4H), 2.21 (s, 3H), 2.05 (dd, *J* = 12.3 Hz, 7.2 Hz, 1H), 1.81 – 1.67 (m, 4H), 1.66 – 1.58 (m, 1H), 1.58 – 1.44 (m, 3H), 1.40 – 1.33 (m, 1H), 1.32 – 1.27 (m, 1H), 1.23 – 1.10 (m, 7H), 1.06 (d, *J* = 9.7 Hz, 1H), 1.04 – 0.96 (m, 1H). ^{13}C NMR (125 MHz, C_6D_6): δ 63.7, 59.7, 41.1, 39.9, 38.1, 37.0, 36.3, 35.6, 30.5, 29.6, 29.1, 28.9, 26.9, 26.5. HR MS (ESI+): Found 222.2221 [M+H]$^+$, calcd. for $C_{15}H_{28}N^+$ 222.2222.

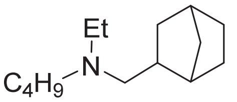

3d: ^{1}H NMR (500 MHz, C_6D_6): δ 2.50 – 2.31 (m, 4H), 2.24 (br, 1H), 2.21 – 2.17 (m, 2H), 2.03 (dd, *J* = 12.5 Hz, 7.1 Hz, 1H), 1.65 – 1.58 (m, 1H), 1.55 – 1.27 (m, 8H), 1.20 – 1.12 (m, 3H), 1.06 (d, *J* = 9.7 Hz, 1H), 0.98 (t, *J* = 7.1 Hz, 3H), 0.92 (t, *J* = 7.2 Hz, 3H). ^{13}C NMR (125 MHz, C_6D_6): δ 60.3, 54.0, 48.2, 41.0, 39.9, 36.9, 36.5, 35.6, 30.5, 30.2, 29.5, 21.0, 14.4, 12.4. HR MS (ESI+): Found: 210.2222 [M+H]$^+$, calcd. for $C_{14}H_{28}N^+$ 210.2222.

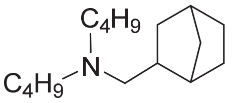

3e: ^{1}H NMR (500 MHz, C_6D_6): δ 2.44 – 2.31 (m, 4H), 2.26 (br, 1H), 2.23 – 2.18 (m, 2H), 2.04 (dd, *J* = 12.5 Hz, 7.0 Hz, 1H), 1.67 – 1.60 (m, 1H), 1.56 – 1.41 (m, 6H), 1.39 – 1.29 (m, 6H), 1.21 – 1.11 (m, 3H), 1.06 (d, *J* = 9.7 Hz, 1H), 0.92 (t, *J* = 7.2 Hz, 6H). ^{13}C NMR (125 MHz, C_6D_6): δ 61.0, 54.7, 41.0, 39.9, 36.9, 36.5, 35.5, 30.5, 30.2, 29.5, 21.0, 14.4. HR MS (ESI+): Found 238.2536 [M+H]$^+$, calcd. for $C_{16}H_{32}N^+$ 238.2535.

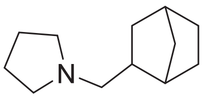

3f: ^{1}H NMR (400 MHz, C_6D_6): δ 2.46 - 2.36 (m, 4H), 2.32 (dd, *J* = 11.6 Hz, 8.8 Hz, 1H), 2.28 (br, 1H), 2.17 (br, 1H), 2.07 (dd, *J* = 11.6 Hz, 7.3 Hz, 1H), 1.67 – 1.60 (m, 5H), 1.51 – 1.44 (m, 2H), 1.41 – 1.35 (m, 1H), 1.30 (ddt, *J* = 9.7 Hz, 3.8 Hz, 1.9 Hz, 1H),

1.22 – 1.10 (m, 3H), 1.07 – 1.03 (m, 1H). ^{13}C NMR (100 MHz, C_6D_6): δ 62.6, 54.6, 41.9, 39.9, 36.9, 36.7, 35.6, 30.5, 29.5, 24.0. HR MS (ESI+): Found 180.1755 $[M+H]^+$, calcd. for $C_{12}H_{22}N^+$ 180.1752.

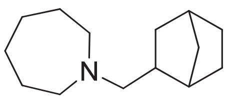

3g: ^{1}H NMR (500 MHz, C_6D_6): δ 2.55 (br, 4H), 2.27 (dd, *J* = 12.1 Hz, 8.5 Hz, 1H), 2.21 – 2.18 (m, 2H), 2.11 (dd, *J* = 12.1 Hz, 7.4 Hz, 1H), 1.57 (br, 9H), 1.51 – 1.44 (m, 2H), 1.37 – 1.30 (m, 1H), 1.28 (d, *J* = 9.5 Hz, 1H), 1.15 (br, 3H), 1.05 (d, *J* = 9.4 Hz, 1H). ^{13}C NMR (125 MHz, C_6D_6): δ 64.5, 56.0, 41.3, 39.9, 37.0, 36.4, 35.7, 30.5, 29.5, 29.3, 27.6. HR MS (ESI+): Found: 208.2066 $[M+H]^+$, calcd. for $C_{14}H_{26}N^+$ 208.2065.

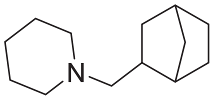

3h: ^{1}H NMR (500 MHz, C_6D_6): δ 2.30 (br, 4H), 2.21 (br, 1H), 2.16 (br, 1H), 2.12 (dd, *J* = 12.2 Hz, 8.6 Hz, 1H), 1.93 (dd, *J* = 12.1 Hz, 7.4 Hz, 1H), 1.66 – 1.61 (m, 1H), 1.56 (s, 2H), 1.56 – 1.51 (m, 4H), 1.48 – 1.44 (m, 1H), 1.35 – 1.27 (m, 4H), 1.18 – 1.10 (m, 3H), 1.03 (d, *J* = 9.7 Hz, 1H). ^{13}C NMR (125 MHz, C_6D_6): δ 65.8, 55.4, 40.0, 37.0, 36.6, 35.6, 30.5, 30.1, 29.5, 26.7, 25.2. HR MS (ESI+): Found 194.1904 $[M+H]^+$, calcd. for $C_{13}H_{24}N^+$ 194.1909.

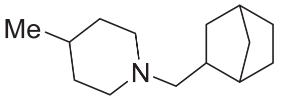

3i: ^{1}H NMR (500 MHz, C_6D_6): δ 2.80 – 2.73 (m, 2H), 2.22 (d, *J* = 9.0 Hz, 1H), 2.17 – 2.12 (m, 2H), 1.99 – 1.91 (m, 1H), 1.77 (m, 1H), 1.71 – 1.63 (m, 2H), 1.61 – 1.42 (m, 7H), 1.40 – 1.27 (m, 2H), 1.20 – 1.10 (m, 3H), 1.05 (d, *J* = 9.7 Hz, 1H), 0.83 (d, *J* = 6.6 Hz, 3H). ^{13}C NMR (125 MHz, C_6D_6): δ 65.5 (x2), 63.2, 62.8, 55.0, 54.6, 40.1, 40.0 (x3), 37.0, 36.6 (x2), 35.6 (x2), 33.7, 31.6, 31.5, 30.5, 29.5, 26.1, 20.0. HR MS (ESI+): Found 208.2065 $[M+H]^+$, calcd. for $C_{14}H_{26}N^+$ 208.2065.

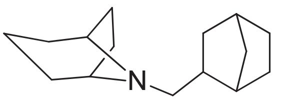

3j: ^{1}H NMR (500 MHz, C_6D_6): δ 3.06 (s, 2H), 2.32 (s, 1H), 2.20 (s, 1H), 2.11 (dd, *J* = 12.1 Hz, 8.5 Hz, 1H), 1.99 (dd, *J* = 12.1 Hz, 7.2 Hz, 1H), 1.85 – 1.78 (m, 4H), 1.58 – 1.47 (m, 3H), 1.44 – 1.35 (m, 5H), 1.32 – 1.30 (m, 1H), 1.22 – 1.13 (m, 5H), 1.07 (d, *J* = 9.1 Hz, 1H). ^{13}C NMR (125 MHz, C_6D_6): δ 57.9, 57.8, 56.7, 39.5, 37.4, 34.5, 34.`, 33.`, 29.1, 29.0, 28.1, 27.1, 24.5, 24.3, 15.0. HR MS (ESI+): Found 220.2065 $[M+H]^+$, calcd. for $C_{15}H_{25}N^+$ 220.2065.

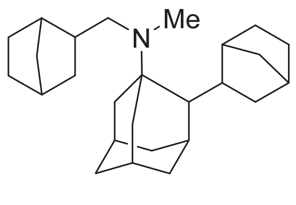

3k: ^{1}H NMR (500 MHz, C_6D_6): δ 2.72 (d, *J* = 3.9 Hz, 1H), 2.53 (t, *J* = 12.5 Hz, 1H), 2.25 (s, 3H), 2.22 – 2.17 (m, 4H), 2.00 (br, 3H), 1.77 (d, *J* = 11.7 Hz, 3H), 1.73 – 1.71 (m, 1H), 1.67 (d, *J* = 11.7 Hz, 3H), 1.61 – 1.45 (m, 12H), 1.43 – 1.40 (m, 1H), 1.35 – 1.17 (m, 5H), 1.15 (d, *J* = 7.5 Hz, 2H), 1.01 (d, *J* = 9.5 Hz, 2H). ^{13}C NMR (125 MHz, C_6D_6): δ 54.0, 52.8, 49.3, 46.0, 43.5, 41.8, 41.4, 41.3, 40.4, 39.3, 37.5, 37.3, 35.4, 34.5, 33.3, 31.8, 30.6, 30.2, 29.7 (x2). HR MS (ESI+): Found 368.3317 $[M+H]^+$, calcd. for $C_{26}H_{42}N^+$ 368.3317.

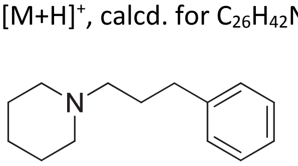

4a: ^{1}H NMR (500 MHz, C_6D_6): δ 7.19 – 7.16 (m, 2H), 7.12 (d, *J* = 7.6 Hz, 2H), 7.08 (t, *J* = 7.6 Hz, 1H), 2.58 (t, *J* = 7.7 Hz, 2H), 2.25 (br, 4H), 2.20 (t, *J* = 7.1 Hz, 2H), 1.73 (quin, *J* = 7.5 Hz, 2H), 1.52 (quin, *J* = 5.5 Hz, 4H), 1.33 (br, 2H). ^{13}C NMR (125 MHz, C_6D_6): δ 142.9, 128.9, 128.6, 126.0, 58.6, 54.9, 33.3, 29.3, 26.6, 25.1. HR MS (ESI+): Found 204.1753 $[M+H]^+$, calcd. for $C_{14}H_{21}N^+$ 204.1752.

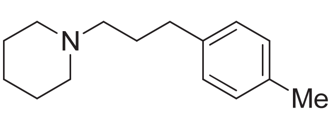

4b: ^{1}H NMR (500 MHz, C_6D_6): δ 7.07 (d, *J* = 7.7 Hz, 2H), 7.02 (d, *J* = 7.7 Hz, 2H), 2.60 (t, *J* = 7.6 Hz, 2H), 2.27 (br, 4H), 2.23 (t, *J* = 7.2 Hz, 2H), 2.16 (s, 3H), 1.76 (quin, *J* = 7.5 Hz, 2H), 1.53 (quin, *J* = 6.0 Hz, 4H), 1.33 (br,

2H). ^{13}C NMR (125 MHz, C_6D_6): δ 139.9, 135.1, 129.3, 128.8, 58.7, 55.0, 33.5, 29.4, 26.7, 25.2, 21.1. HR MS (ESI+): Found 218.1906 $[M+H]^+$, calcd. for $C_{15}H_{24}N^+$ 218.1909.

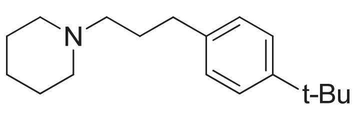

4c: ^{1}H NMR (500 MHz, C_6D_6): δ 7.28 (d, *J* = 8.1 Hz, 2H), 7.14 (d, *J* = 8.1 Hz, 2H), 2.63 (t, *J* = 7.6 Hz, 2H), 2.27 (br, 4H), 2.24 (t, *J* = 7.0 Hz, 2H), 1.78 (quin, *J* = 7.5 Hz, 2H), 1.53 (quin, *J* = 5.5 Hz, 4H), 1.33 (m, 2H), 1.26 (s, 9H). ^{13}C NMR (125 MHz, C_6D_6): δ 148.1, 139.5, 128.2, 125.1, 58.4, 54.6, 34.0, 33.1, 31.2, 29.0, 26.3, 24.8. HR MS (ESI+): Found 260.2373 $[M+H]^+$, calcd. for $C_{18}H_{30}N^+$ 260.2373.

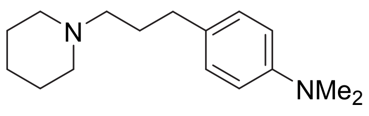

4d: ^{1}H NMR (500 MHz, C_6D_6): δ 7.15 (d, *J* = 8.3 Hz, 2H), 6.67 (d, *J* = 8.5 Hz, 2H), 2.65 (t, *J* = 7.5 Hz, 2H), 2.56 (s, 6H), 2.31 – 2.28 (m, 6H), 1.83 (quin, *J* = 7.5 Hz, 2H), 1.54 (quin, *J* = 6.0 Hz, 4H), 1.34 (br, 2H). ^{13}C NMR (125 MHz, C_6D_6): δ 149.5, 131.1, 129.4, 113.5, 55.8, 55.0, 40.7, 33.1, 29.8, 26.7, 25.2. HR MS (ESI+): Found 247.2177 $[M+H]^+$, calcd. for $C_{16}H_{27}N_2^+$ 247.2174.

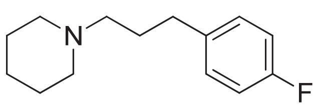

4e: ^{1}H NMR (500 MHz, C_6D_6): δ 6.87 - 6.80 (m, 4H), 2.45 (t, *J* = 7.6 Hz, 2H), 2.24 (br, 4H), 2.14 (t, *J* = 7.0 Hz, 2H), 1.62 (quin, *J* = 7.5 Hz, 2H), 1.52 (quin, *J* = 5.5 Hz, 4H), 1.3 (br, 2H). ^{13}C NMR (125 MHz, C_6D_6): δ 159.8 (*J* = 242.8 Hz), 136.5 (*J* = 3.2 Hz), 128.2 (*J* = 7.7 Hz), 113.3 (*J* = 21.0 Hz), 56.4, 53.0, 31.0, 27.3, 24.7, 23.2. HR MS (ESI+): Found 222.1657 $[M+H]^+$, calcd. for $C_{14}H_{21}FN^+$ 222.1658.

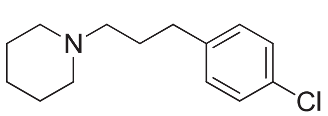

4f: ^{1}H NMR (500 MHz, C_6D_6): δ 7.12 (d, *J* = 8.3 Hz, 2H), 6.79 (d, *J* = 8.3 Hz, 2H), 2.40 (t, *J* = 7.6 Hz, 2H), 2.23 (br, 4H), 2.12 (t, *J* = 7.0 Hz, 2H), 1.58 (quin, *J* = 7.5 Hz, 2H), 1.52 (quin, *J* = 5.5 Hz, 4H), 1.33 (br, 2H). ^{13}C NMR (125 MHz, C_6D_6): δ 141.3, 131.8, 130.2, 128.7, 58.3, 54.9, 37.0, 29.0, 26.6, 25.1HR MS (ESI+): Found 238.1357 $[M+H]^+$, calcd. for $C_{14}H_{21}ClN^+$ 238.1363.

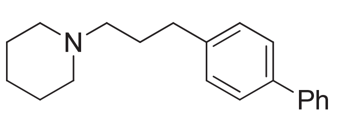

4g: ^{1}H NMR (500 MHz, C_6D_6): δ 7.52 (d, *J* = 7.2 Hz, 2H), 7.47 (d, *J* = 8.1 Hz, 2H), 7.23 (t, *J* = 7.6 Hz, 2H), 7.17 – 7.12 (m, 3H), 2.62 (t, *J* = 7.6 Hz, 2H), 2.28 (br, 4H), 2.24 (t, *J* = 7.1 Hz, 2H), 1.77 (quin, *J* = 7.0 Hz, 2H), 1.54 (quin, *J* = 5.5 Hz, 4H), 1.34 (br, 2H). ^{13}C NMR (125 MHz, C_6D_6): δ 142.0, 141.8, 139.2, 129.3, 129.0, 127.4 (x2), 127.2, 58.6, 55.0, 33.5, 29.3, 26.7, 25.2. HR MS (ESI+): Found 280.2060 $[M+H]^+$, calcd. for $C_{20}H_{26}N^+$ 280.2065.

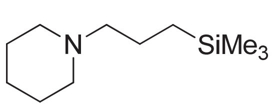

4h: ^{1}H NMR (500 MHz, C_6D_6): δ 2.34 (br, 4H), 2.27 (t, *J* = 7.5 Hz, 2H), 1.58 – 1.50 (m, 6H), 1.35 (br, 2H) 0.53 – 0.49 (m, 2H), 0.01 (s, 9H). ^{13}C NMR (125 MHz, C_6D_6): δ 63.2, 55.1, 26.7, 25.2, 22.1, 14.5 HR MS (ESI+): Found 200.1834 $[M+H]^+$, calcd. for $C_{11}H_{26}NSi^+$ 200.1835.

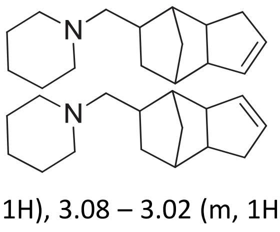

5a: ^{1}H NMR (500 MHz, C_6D_6): **5a** δ 5.64 – 5.59 (m, 1H), 5.53 – 5.51 (m, 1H), 3.01 – 2.95 (m, 1H), 2.48 – 2.41 (m, 1H), 2.38 – 2.23 (br m, 5H), 2.21 – 2.12 (m, 3H), 2.10 (dd, *J* = 7.9 Hz, 4.0Hz, 1H), 2.01 – 1.90 (m, 1H), 1.84 – 1.77 (m, 1H), 1.56 – 1.51 (m, 5H), 1.43 (d, *J* = 9.6 Hz, 1H) 1.36 – 1.25 (m, 3H), 0.931 (dtd, *J* = 12.0 Hz, 5.5Hz, 1.5 Hz, 1H). **5a'** δ 5.64 – 5.59 (m, 1H), 5.53 – 5.51 (m, 1H), 3.08 – 3.02 (m, 1H), 2.48 – 2.41 (m, 1H), 2.38 – 2.23 (br m, 5H), 2.21 – 2.12 (m, 3H), 2.08 – 2.06 (br, 1H), 2.01 – 1.90 (m, 2H), 1.56 – 1.51 (m, 6H), 1.36 – 1.25 (m, 3H), 1.01 (dtd, *J* = 12.9 Hz, 4.4Hz, 1.7 Hz, 1H); ^{13}C NMR (125 MHz, C_6D_6): **5a** δ 132.9, 130.7, 65.9, 55.3, 53.1, 44.7, 42.9, 40.4, 38.7, 32.5, 32.2,

31.3, 26.7, 25.2. **5a'** δ 132.7, 131.4, 65.6, 55.4, 54.1, 43.1, 42.5, 41.5, 38.8, 34.3, 32.7, 29.0, 26.7, 25.2; HR MS (ESI+): Found 232.2070 $[M+H]^+$, calcd. for $C_{16}H_{26}N^+$ 232.2065.

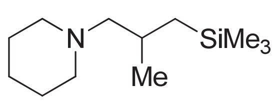

5b: ^{1}H NMR (500 MHz, C_6D_6): δ 2.30 (br, 4H), 2.04 (ddd, *J* = 28.9 Hz, 12.0 Hz, 7.3 Hz, 2H), 1.81 (td, *J* = 13.7 Hz, 6.8 Hz, 1H), 1.53 (quin, *J* = 6.0 Hz, 4H), 1.34 (br, 2H), 1.02 (d, *J* = 6.5 Hz, 3H), 0.84 (dd, *J* = 14.7 Hz, 4.5 Hz, 1H), 0.36 – 0.28 (m, 1H), 0.07 (s, 9H). ^{13}C NMR (125 MHz, C_6D_6): δ 69.8, 55.5, 27.6, 26.7, 25.1, 23.3, 21.8, -0.37. HR MS (ESI+): Found 214.1987 $[M+H]^+$, calcd. for $C_{12}H_{28}NSi^+$ 214.1991.

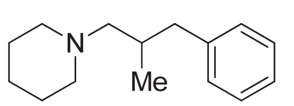

5c: ^{1}H NMR (500 MHz, C_6D_6): δ 7.19 (t, *J* = 7.4 Hz, 2H), 7.13 – 7.08 (m, 3H), 2.84 (dd, *J* = 13.3 Hz, 4.7 Hz, 1H), 2.31 – 2.26 (br m, 5H), 2.09 (dd, *J* = 12.0 Hz, 7.6 Hz, 1H), 1.99 (dd, *J* = 12.0 Hz, 7.1 Hz, 1H), 1.96 – 1.88 (m, 1H), 1.52 (quin, *J* = 6.0 Hz, 4H), 1.33 (br, 2H), 0.87 (d, *J* = 6.5 Hz, 3H). ^{13}C NMR (125 MHz, C_6D_6): δ 141.6, 129.7, 128.4 (x2), 126.0, 66.0, 55.3, 41.6, 33.0, 26.7, 25.1, 18.2. HR MS (ESI+): Found 218.1904 $[M+H]^+$, calcd. for $C_{15}H_{24}N^+$ 218.1909.

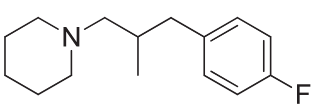

5d: ^{1}H NMR (500 MHz, C_6D_6): δ 6.88 - 6.81 (m, 4H), 2.69 (dd, *J* = 13.4 Hz, 4.7 Hz, 1H), 2.24 (br, 4H), 2.17 (dd, *J* = 13.4 Hz, 8.4 Hz, 1H), 2.02 (dd, *J* = 12.51 Hz, 7.9 Hz, 1H), 1.95 (dd, *J* = 12.2 Hz, 6.9 Hz, 1H), 1.85 – 1.76 (m, 1H), 1.52 (quin, *J* = 5.5 Hz, 4H), 1.33 (br, 2H), 0.80 (d, *J* = 6.6 Hz, 3H).^{13}C NMR (125 MHz, C_6D_6): δ 161.8 (d, *J* = 242.9 Hz), 137.1 (d, *J* = 3.2 Hz), 131.0 (d, *J* = 7.6 Hz), 115.1 (d, *J* = 20.9 Hz), 65.8, 55.3, 40.5, 32.9, 26.6, 25.1, 18.0. HR MS (ESI+): Found 236.1817 $[M+H]^+$, calcd. for $C_{15}H_{23}FN^+$ 236.1815.

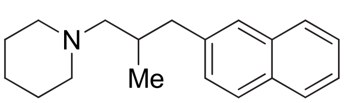

5e: ^{1}H NMR (500 MHz, C_6D_6): δ 8.29 (d, *J* = 8.5 Hz, 1H), 7.72 (d, *J* = 8.2 Hz, 1H), 7.61 (d, *J* = 8.1 Hz, 1H), 7.41 (t, *J* = 7.6 Hz, 1H), 7.33 – 7.27 (m, 2H), 7.23 (d, *J* = 6.9 Hz, 1H), 3.60 (dd, *J* = 13.5 Hz, 8.5 Hz, 1H), 2.46 (dd, *J* = 13.5 Hz, 8.9 Hz, 1H), 2.35 (br, 2H), 2.24 (br, 2H), 2.17 – 1.98 (m, 3H), 1.56 (quin, *J* = 5.5 Hz, 4H), 1.35 (br, 2H), 0.85 (d, *J* = 6.4 Hz, 3H).^{13}C NMR (125 MHz, C_6D_6): δ 138.4, 134.8, 133.0, 129.2, 127.5, 127.0, 125.8, 125.6 (x2), 124.9, 67.0, 55.7, 39.3, 32.8, 26.7, 25.1, 18.7. HR MS (ESI+): Found 268.2063 $[M+H]^+$, calcd. for $C_{19}H_{26}N^+$ 268.2065.

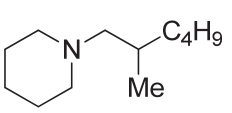

5f: ^{1}H NMR (500 MHz, C_6D_6): δ 2.29 (br, 4H), 2.10 (dd, *J* = 12.1 Hz, 6.7 Hz, 1H), 2.00 (dd, *J* = 12.1 Hz, 7.9 Hz, 1H), 1.68 – 1.59 (m, 1H), 1.54 (quin, *J* = 5.5 Hz, 4H), 1.51 – 1.45 (m, 1H), 1.38 – 1.22 (m, 6H), 1.12 – 1.05 (m, 1H), 0.97 (d, *J* = 6.6 Hz, 3H), 0.92 (t, *J* = 7.0 Hz, 3H). ^{13}C NMR (125 MHz, C_6D_6): δ 67.0, 55.5, 35.4, 30.9, 29.7, 26.7, 25.2, 23.5, 18.7, 14.4. HR MS (ESI+): Found 184.2064 $[M+H]^+$, calcd. for $C_{12}H_{26}N^+$ 184.2065.

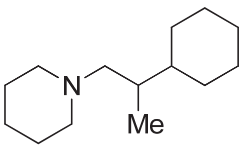

5g: ^{1}H NMR (500 MHz, C_6D_6): δ 2.32 (br m 2H), 2.25 (br, 2H), 2.20 (dd, *J* = 12.1 Hz, 6.2 Hz, 1H), 2.01 (dd, *J* = 12.1 Hz, 8.6 Hz, 1H), 1.75 – 1.72 (m, 2H), 1.68 – 1.65 (m, 1H), 1.60 – 1.51 (m, 7H), 1.36 -1.31 (m, 3H), 1.27 – 1.20 (m, 2H), 1.17 – 1.08 (m, 2H), 1.06 -0.97 (m, 1H), 0.94 (d, *J* = 6.8 Hz, 3H).^{13}C NMR (125 MHz, C_6D_6): δ 64.4, 55.6, 41.3, 35.8, 31.5, 28.7, 27.4, 27.3, 27.2, 26.7, 25.2, 15.1.HR MS (ESI+): Found 210.2224 $[M+H]^+$, calcd. for $C_{14}H_{28}N^+$ 210.2222.

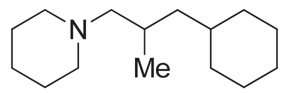

5h: ^{1}H NMR (500 MHz, C_6D_6): δ 2.31 – 2.28 (br, 4H), 2.09 (dd, *J* = 12.0 Hz, 6.7 Hz, 1H), 2.00 (dd, *J* = 12.0 Hz, 7.9 Hz, 1H), 1.89 – 1.64 (m, 6H), 1.54 (quin, *J* = 6.0 Hz, 4H), 1.44 – 1.30 (m, 4H), 1.27 – 1.10 (m, 3H), 0.99 – 0.79 (m, 3H), 0.96 (d, *J* = 6.5 Hz, 3H). ^{13}C NMR (125 MHz, C_6D_6): δ 67.4, 55.5, 44.0, 35.4, 34.8, 33.5, 27.7, 27.2, 26.9, 26.8, 26.7, 25.2, 19.2. HR MS (ESI+): Found 224.2378 $[M+H]^+$, calcd. for $C_{15}H_{30}N^+$ 224.2378.

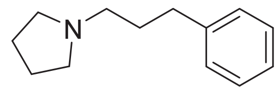

6a: ^{1}H NMR (500 MHz, C_6D_6): δ 7.18 (d, *J* = 7.5 Hz, 2H), 7.11 (d, *J* = 7.3 Hz, 2H) 7.08 (t, *J* = 7.3 Hz, 1H), 2.61 (t, *J* = 7.6 Hz, 2H), 2.35 – 2.32 (m, 6H), 1.74 (quin, *J* = 7.5 Hz, 2H), 1.61 – 1.55 (m, 4H). ^{13}C NMR (125 MHz, C_6D_6): δ 142.7, 128.8, 128.7, 126.1, 55.7, 54.2, 33.9, 30.9, 23.9. HR MS (ESI+): Found 190.1599 $[M+H]^+$, calcd. for $C_{13}H_{20}N^+$ 190.1596.

5. Kinetic Isotope Effect Experiments

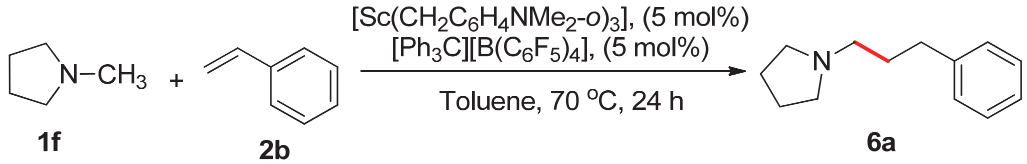

Compound **6a** could be synthesized following the general procedure above in an 81 % isolated yield.

Intermolecular Competition Experiment

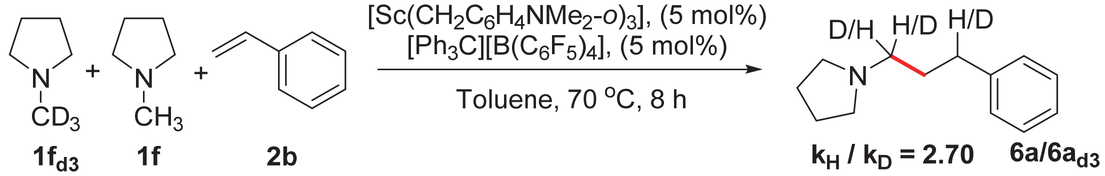

In a glovebox, $[Sc(CH_2C_6H_4NMe_2\text{-}o)_3]$ (6 mg, 0.013 mmol) was dissolved in toluene (1.0 mL). To this solution $\mathbf{1f_{d3}}$ (22 mg, 0.25 mmol), **1f** (21 mg, 0.25 mmol), styrene (26 mg, 0.25 mmol) and $[Ph_3C][B(C_6F_5)_4]$ (16 mg, 0.013 mmol) were added sequentially. The biphasic mixture was transferred to a Schlenk ampoule, sealed and heated at 70 °C for 8 hours. EtOAc (5 mL) was added to the crude mixture and the volatiles removed *in vacuo.* The compound was purified by silica gel column chromatography (hexane/EtOAc), to afford a mixture of **6a** and $\mathbf{6a_{d3}}$ as a colorless oil (12 mg, 25 % yield). A k_H/k_D value of 2.70 was found by comparison of the relative ratios of **6a** and $\mathbf{6a_{d3}}$ by ^{1}H NMR spectroscopy using the integration values for the benzylic proton(s). No deuterium scrambling was observed when this mixture of **6a** and $\mathbf{6a_{d3}}$ was exposed to the standard reaction conditions.

Fig. S1. The ^{1}H NMR Spectrum of an Isolated Mixture of 6a and $6a_{d3}$

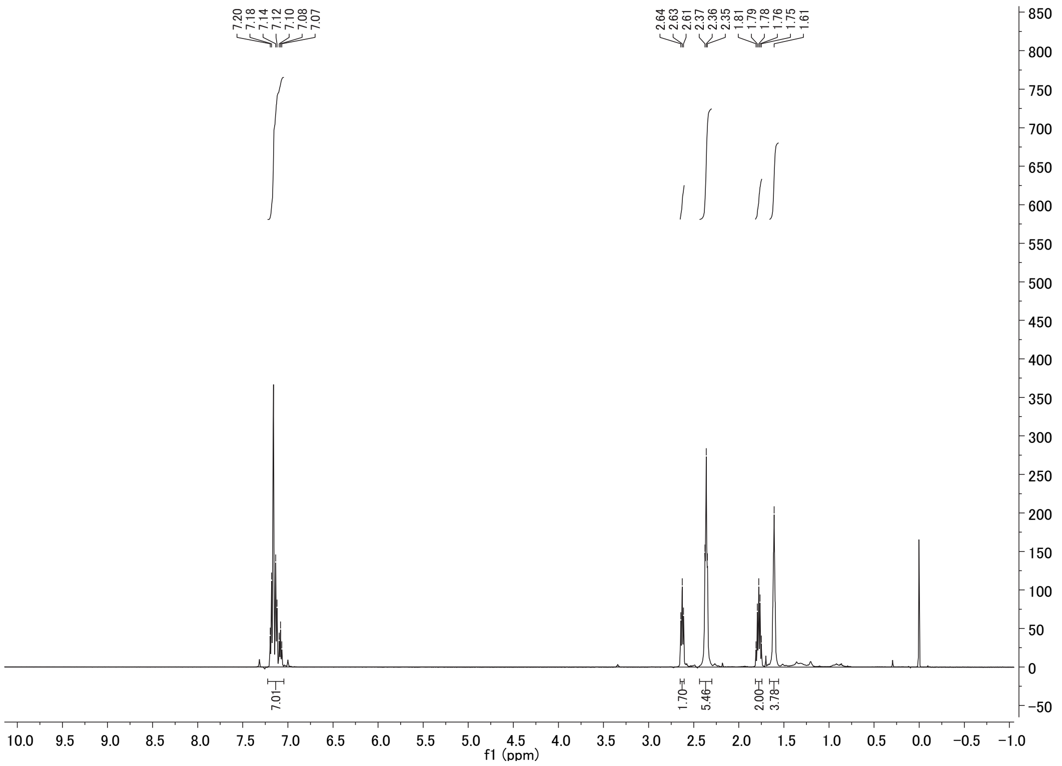

Comparison of Initial Rates

In a glovebox, [Sc($CH_2C_6H_4NMe_2$-*o*)$_3$] (6 mg, 0.013 mmol) was dissolved in C_7D_8 (1.0 mL). To this solution, ferrocene (19 mg, 0.1 mmol), *N*-methylpyrrolidine (21mg, 0.25 mmol), styrene (29 mg, 0.28 mmol) and [Ph_3C][B(C_6F_5)$_4$] (16 mg, 0.013 mmol) were added sequentially. The biphasic mixture was transferred to a J Young NMR tube, sealed and the reaction monitored by ^{1}H NMR at 70 ^{o}C for 12 h. The reaction with d$_3$-*N*-methylpyrrolidine was performed and monitored under exactly the same conditions. Initial rates were extracted by comparing the ^{1}H NMR integration of the benzylic proton(s) of **6a** and **6a$_{d3}$** against the internal standard. A k_H/k_D value of 1.97 ± 0.03 was found by comparison of the initial rates of reaction.

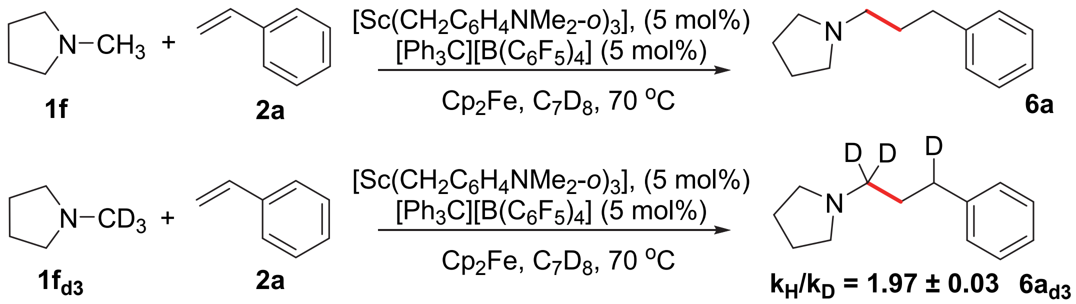

Fig. S2. Initial Rates of C–H/C–D Addition of *N*-methylpyrrolidine to Styrene

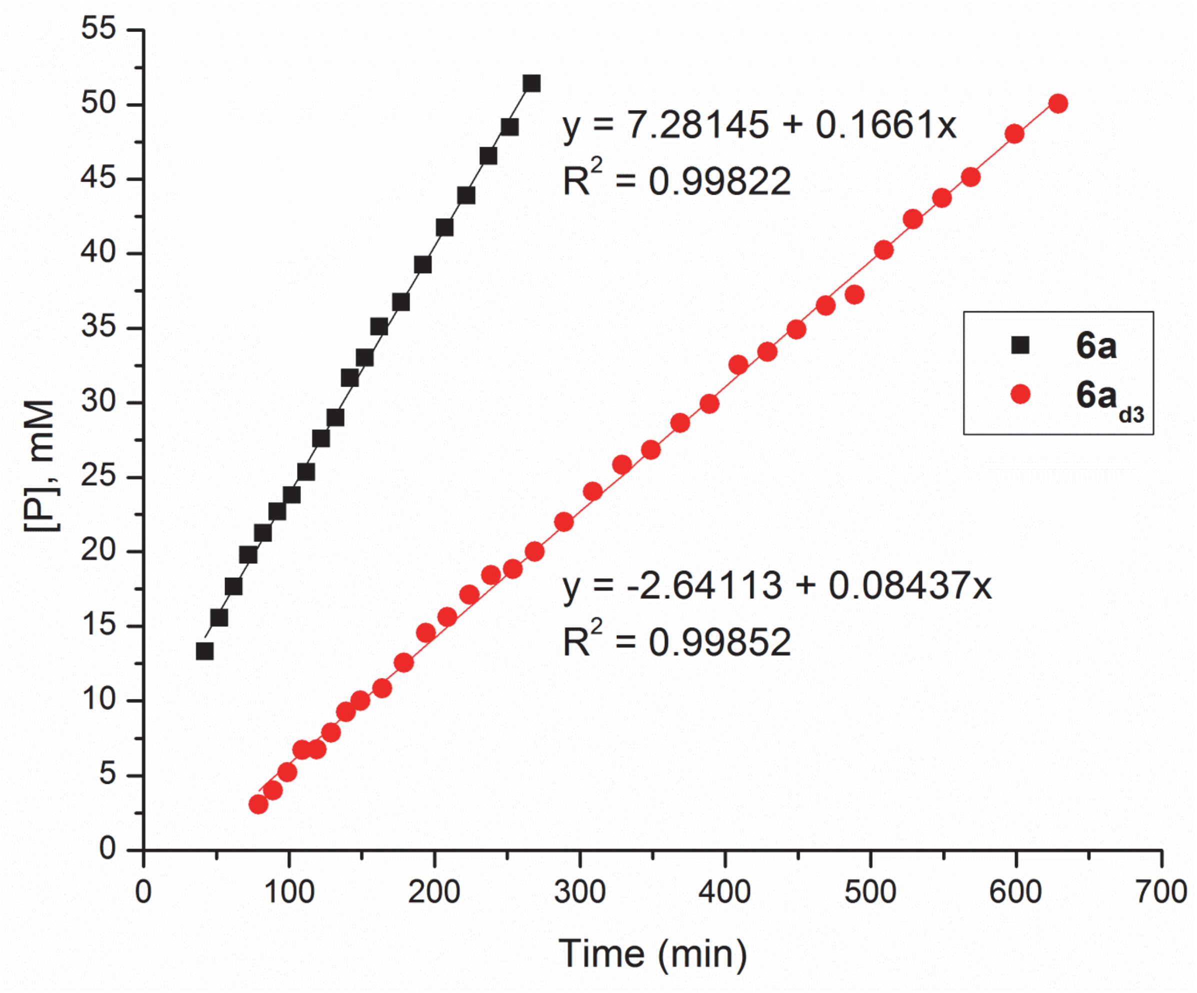

6. Dialkylation of 1k to give 3k

Scheme S1. A Plausible Mechanism for the Formation of 3k

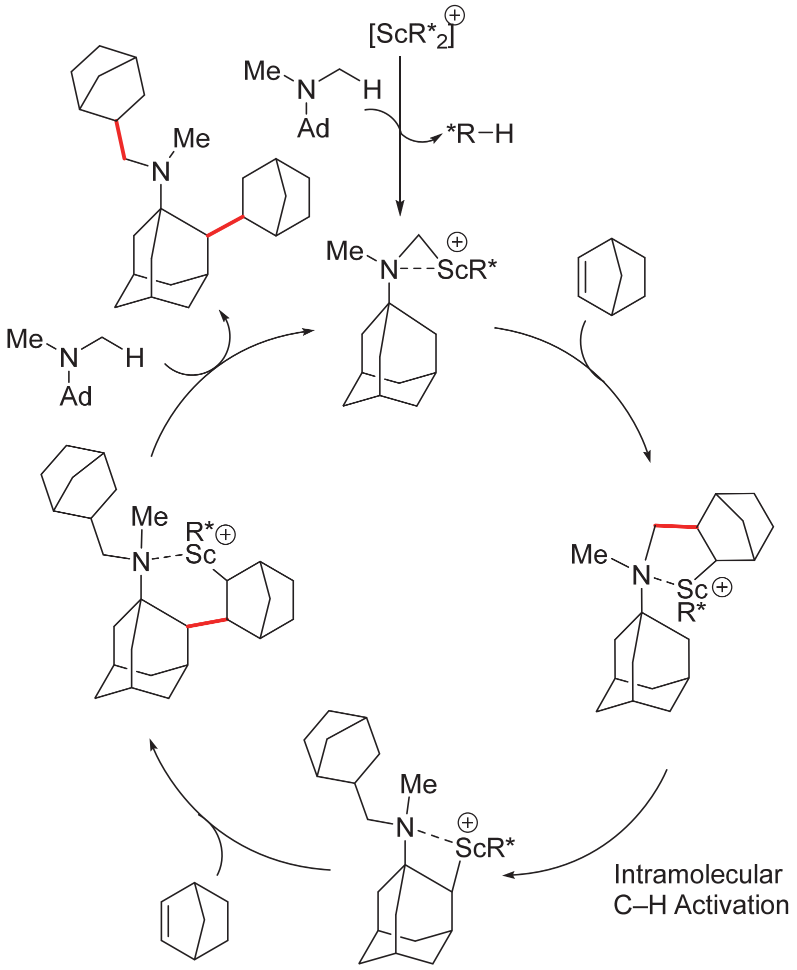

7. References

(1) (a) L. E. Manzer, *J. Am. Chem. Soc.*, 1978, **100**, 8068; (b) W. X. Zhang, M. Nishiura, T. Mashiko and Z. Hou, *Chem. Eur. J.*, 2008, **14**, 2167.

(2) X. Li, M. Nishiura, K. Mori, T. Mashiko and Z. Hou, *Chem. Commun.*, 2007, 4137.

(3) R. N. Icke, B. B. Wisegarver, and G. A. Alles *Org. Synth.*, 1945, **25**, 89.

(4) P. Wei, X. Bi, Z. Wu and Z. Xu, *Org. Lett.*, 2005, **7**, 3199.

8. ^{1}H and ^{13}C NMR spectra of products

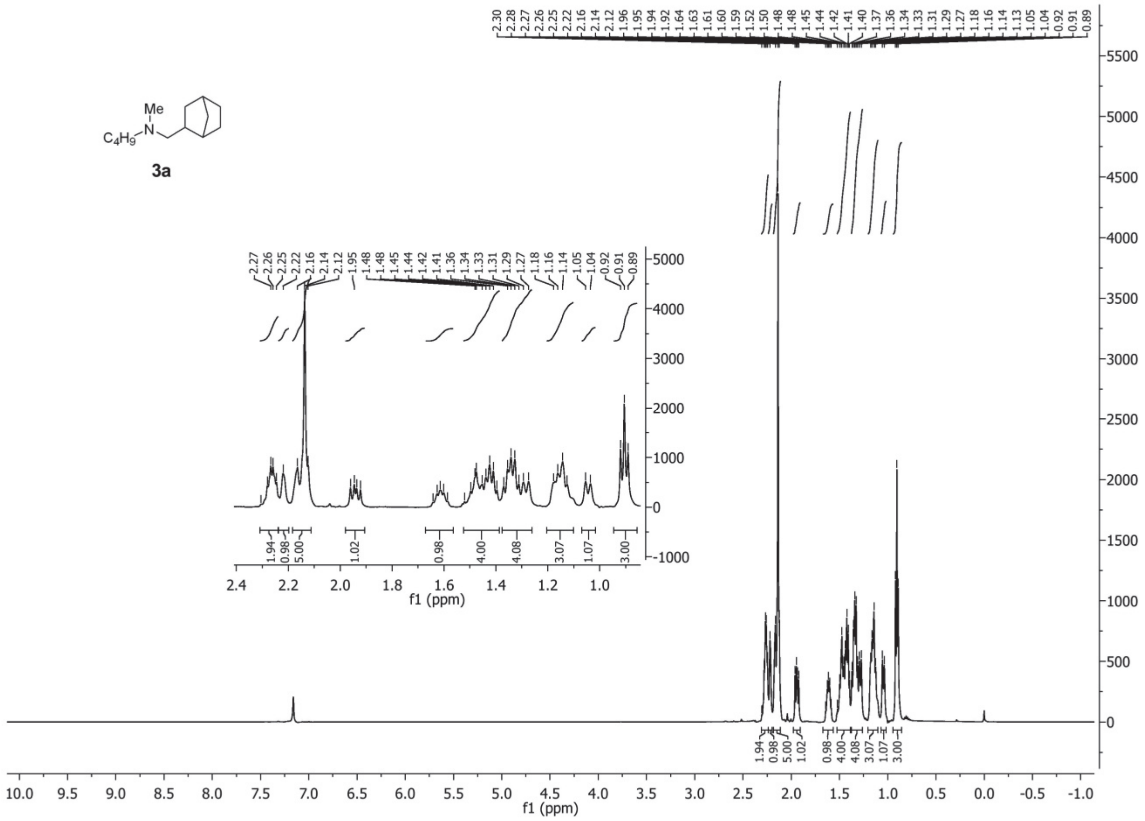

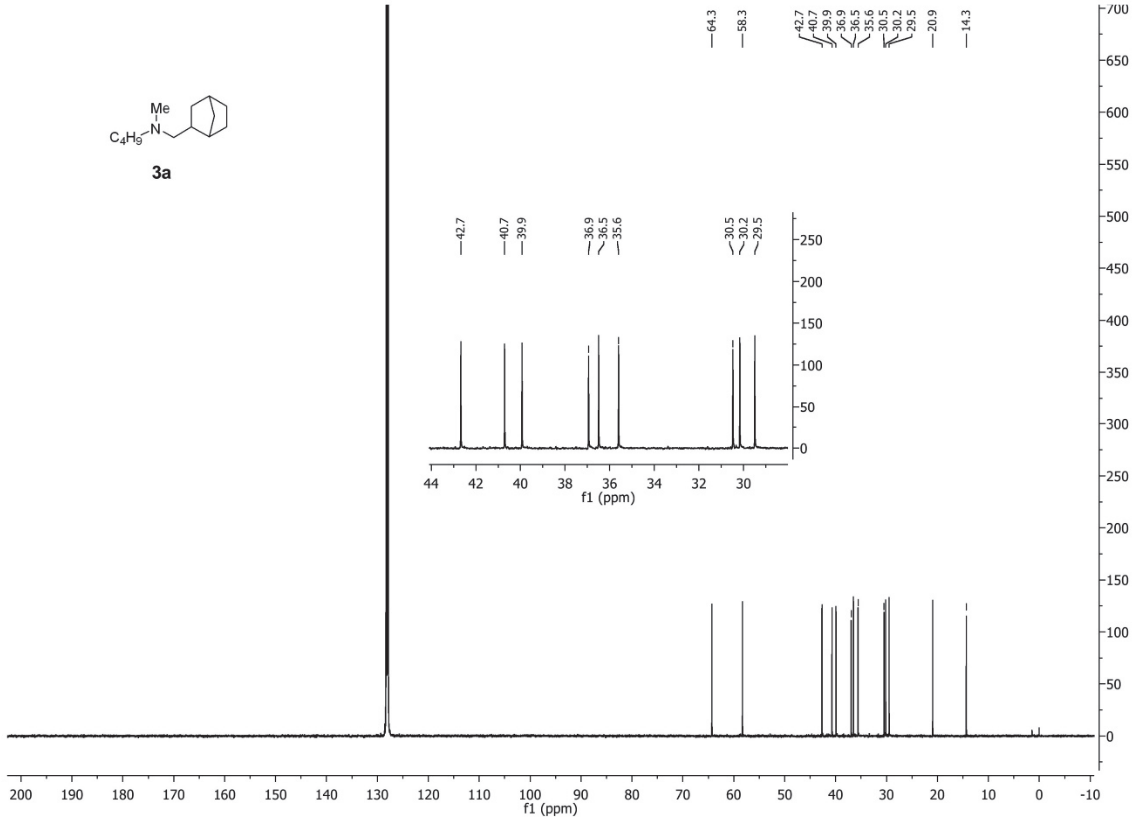

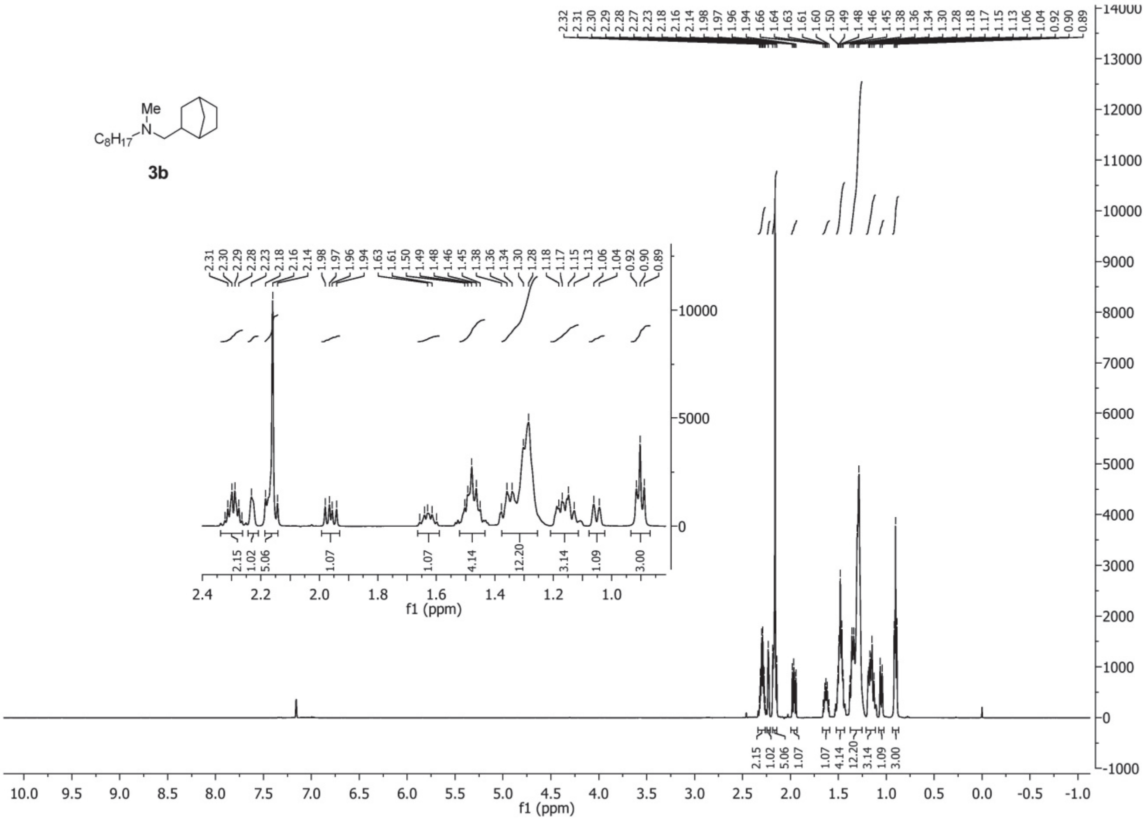
Me
N
C_8H_{17}
3b
f1 (ppm)

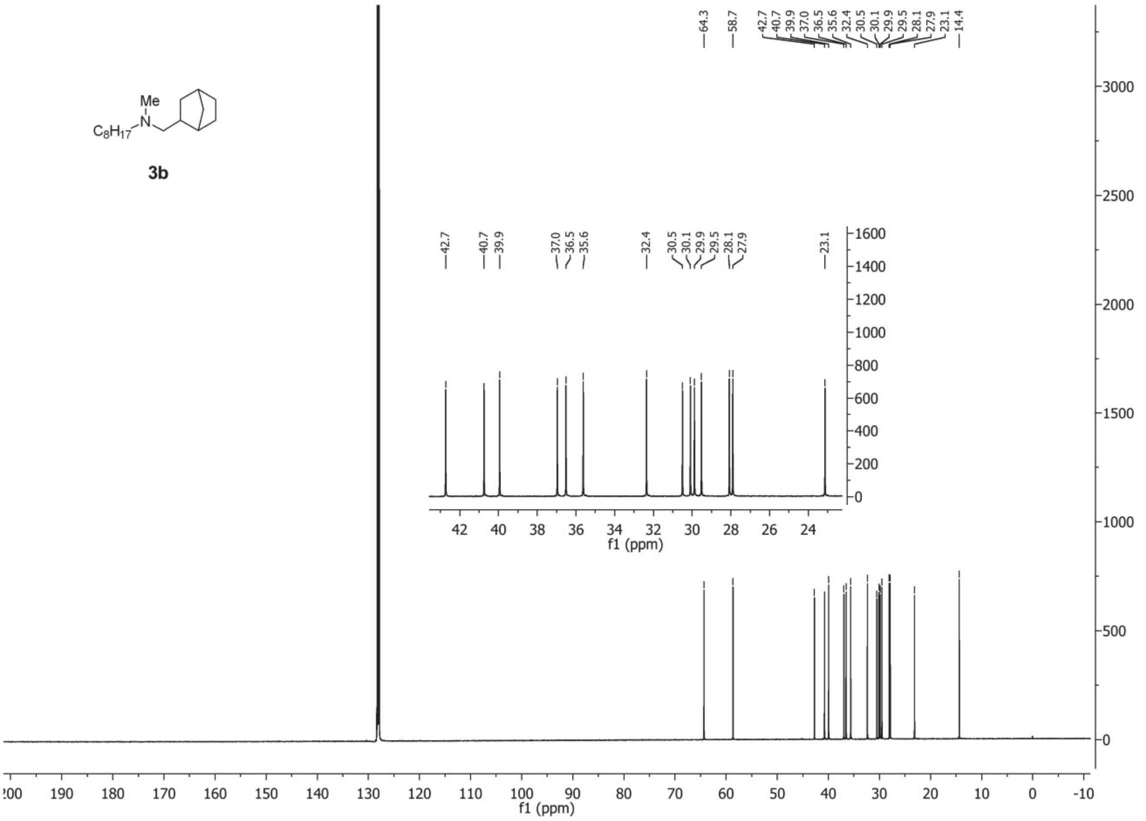
Me
N
C_8H_{17}
3b
f1 (ppm)

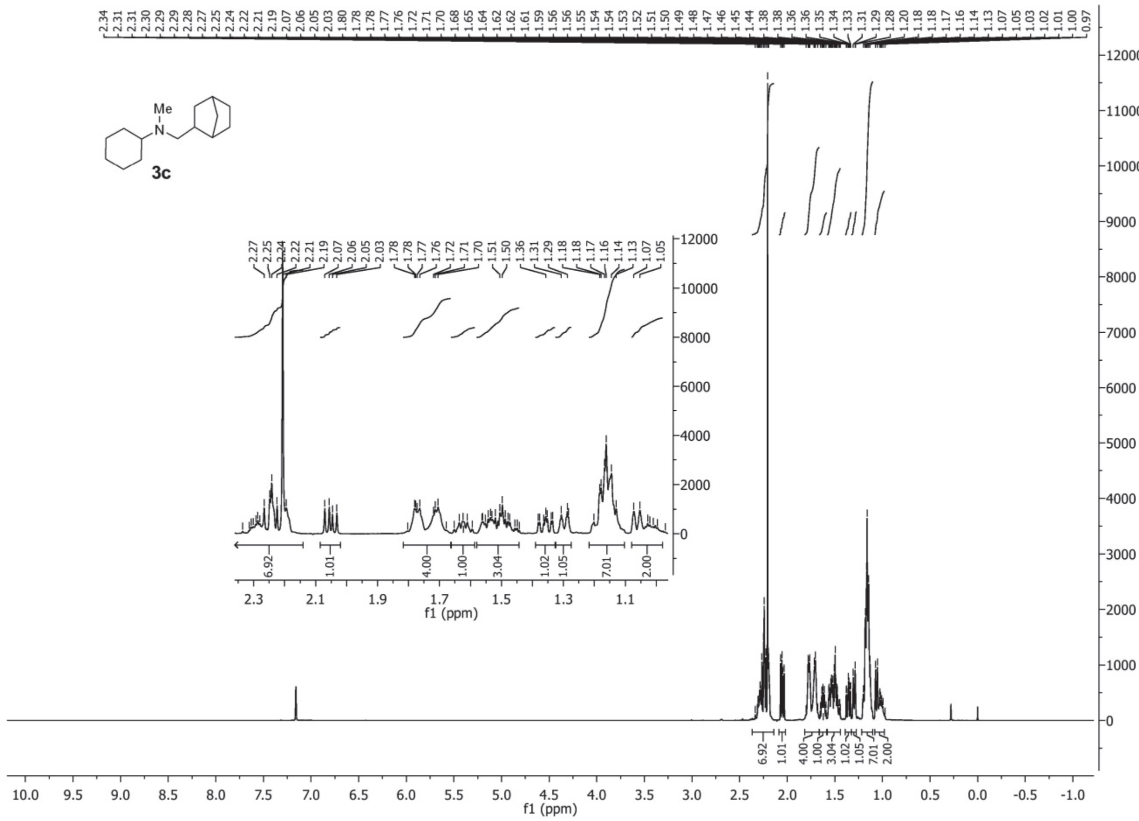
Me
N
3c
f1 (ppm)

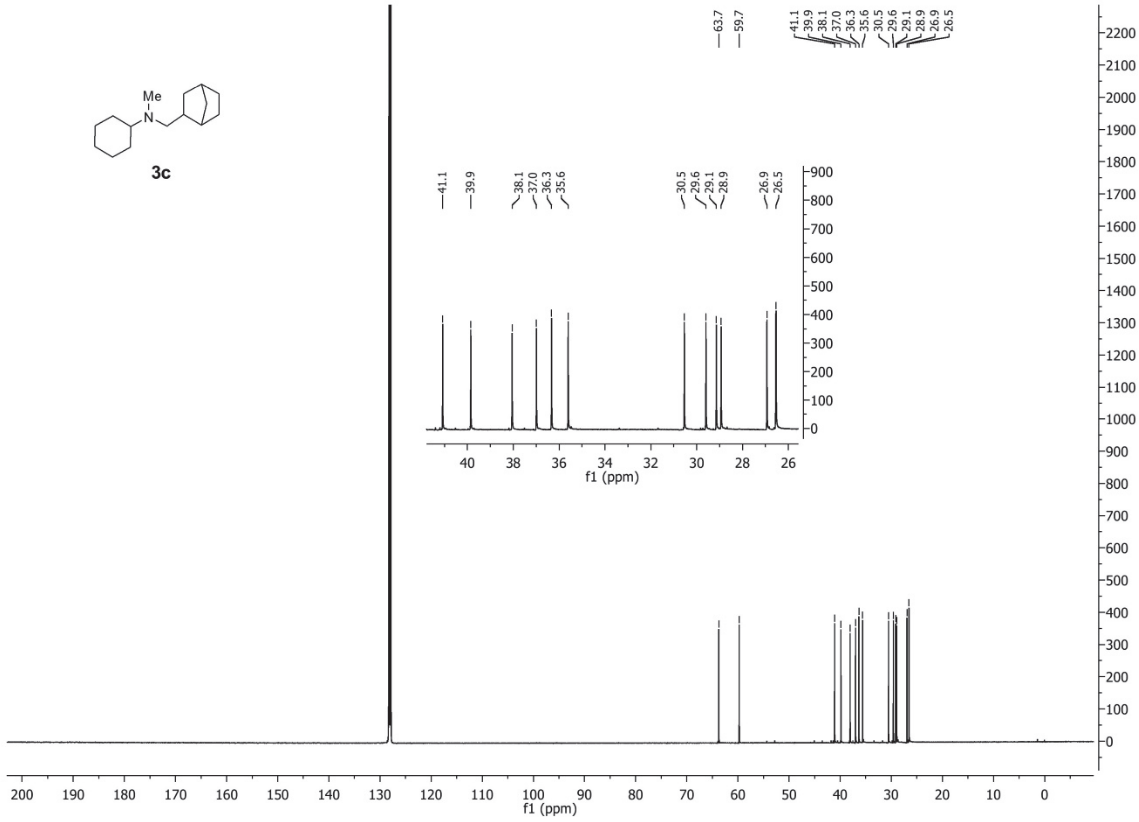
Me
N
3c
63.7
59.7
41.1
39.9
38.1
37.0
36.3
35.6
30.5
29.6
29.1
28.9
26.9
26.5
f1 (ppm)

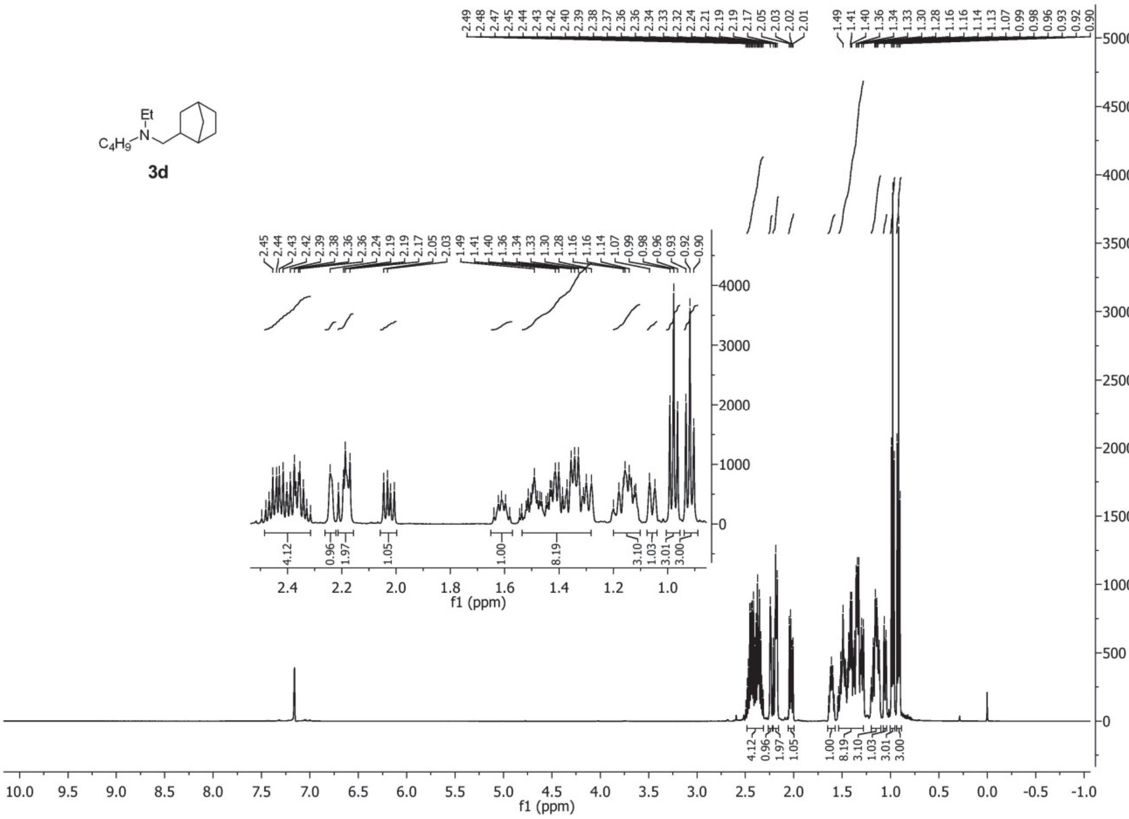
2.49 2.48 2.47 2.45 2.44 2.43 2.42 2.40 2.39 2.38 2.37 2.36 2.36 2.34 2.33 2.32 2.24 2.21 2.19 2.19 2.17 2.05 2.03 2.02 2.01
1.49 1.41 1.40 1.36 1.34 1.33 1.30 1.28 1.16 1.16 1.14 1.13 1.07 0.99 0.98 0.96 0.93 0.92 0.90
Et
N
C_4H_9
3d
4.12 0.96 1.97 1.05 1.00 8.19 3.10 1.03 3.01 3.00
f1 (ppm)

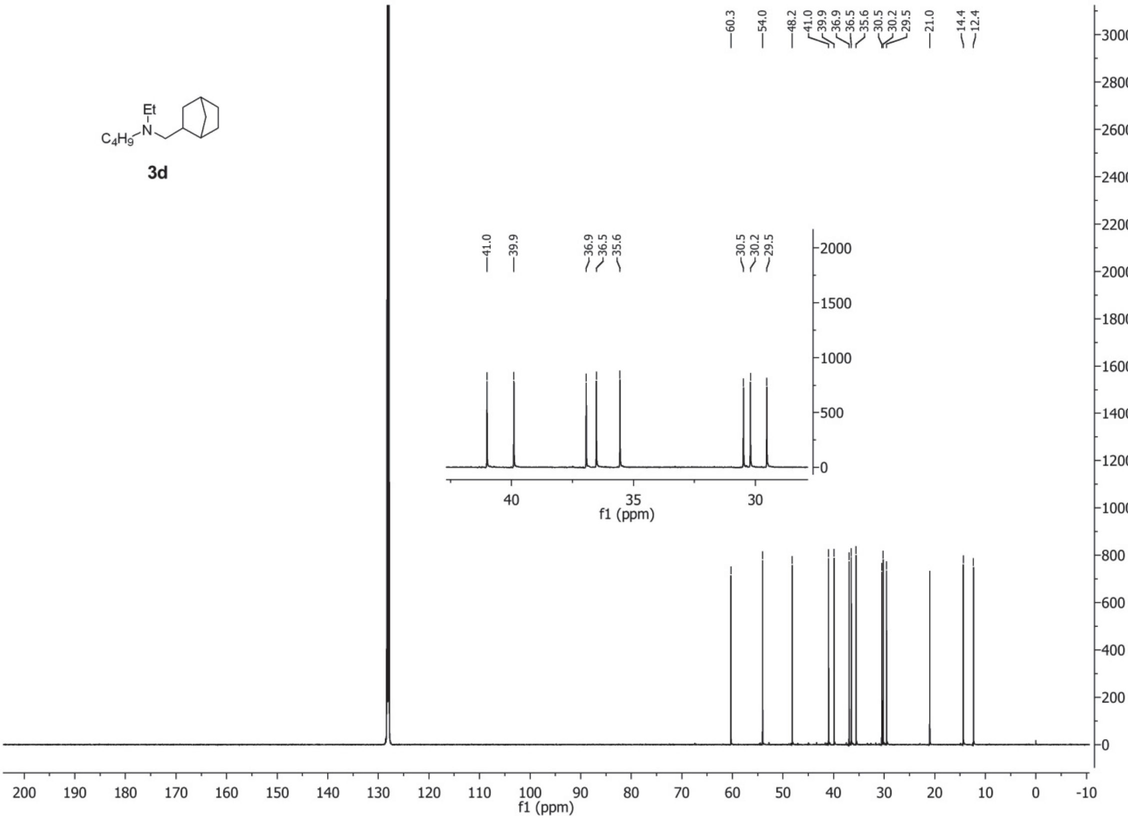
60.3 54.0 48.2 41.0 39.9 36.9 36.5 35.6 30.5 30.2 29.5 21.0 14.4 12.4
Et
N
C_4H_9
3d
f1 (ppm)

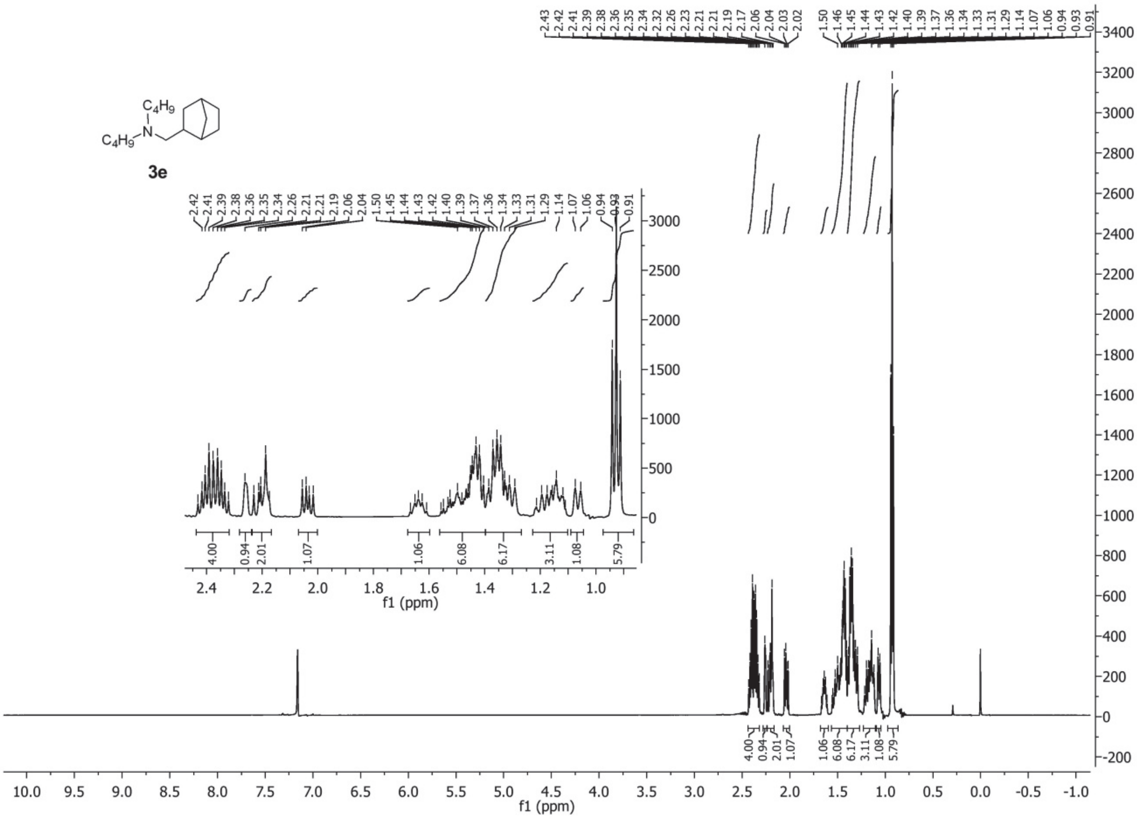
2.43 2.42 2.41 2.39 2.38 2.36 2.35 2.34 2.32 2.26 2.23 2.21 2.21 2.19 2.17 2.06 2.04 2.03 2.02
1.50 1.46 1.45 1.44 1.43 1.42 1.40 1.39 1.37 1.36 1.34 1.33 1.31 1.29 1.14 1.07 1.06 0.94 0.93 0.91
C_4H_9
C_4H_9
N
3e
4.00 0.94 2.01 1.07 1.06 6.08 6.17 3.11 1.08 5.79
f1 (ppm)

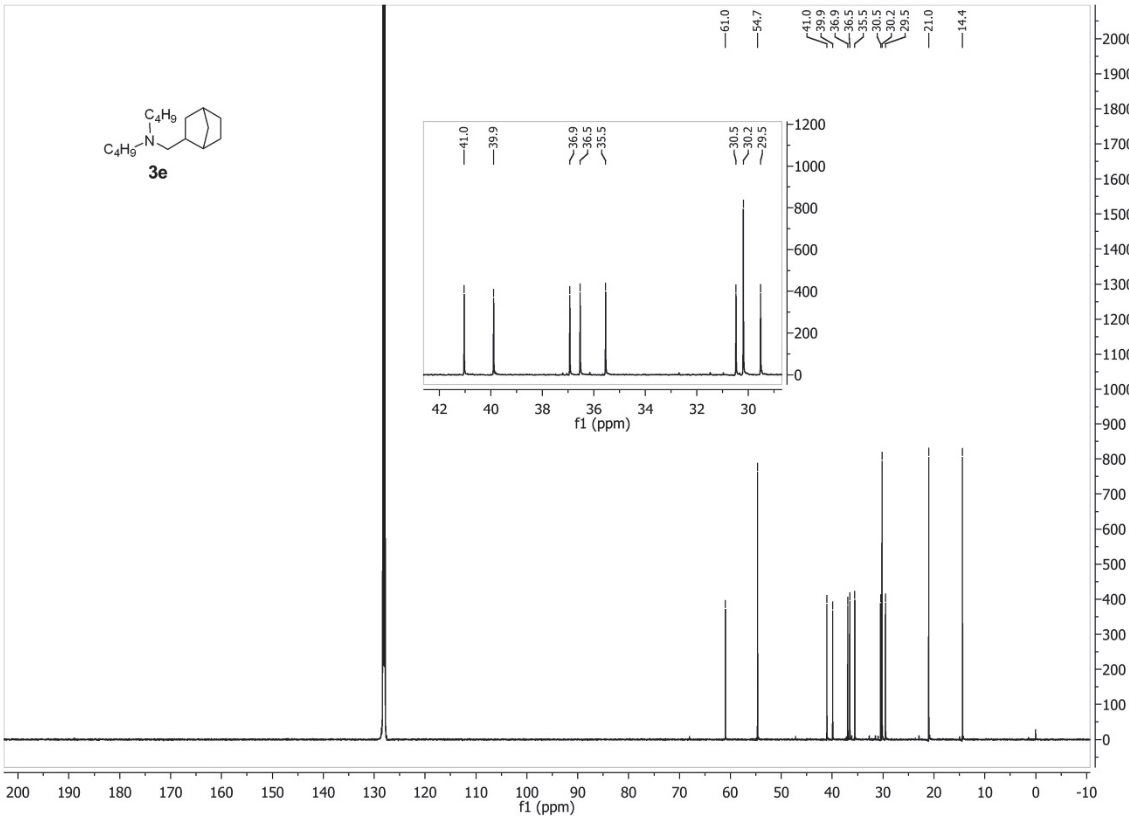
61.0
54.7
41.0 39.9 36.9 36.5 35.5 30.5 30.2 29.5
21.0
14.4
C_4H_9
C_4H_9
N
3e
f1 (ppm)

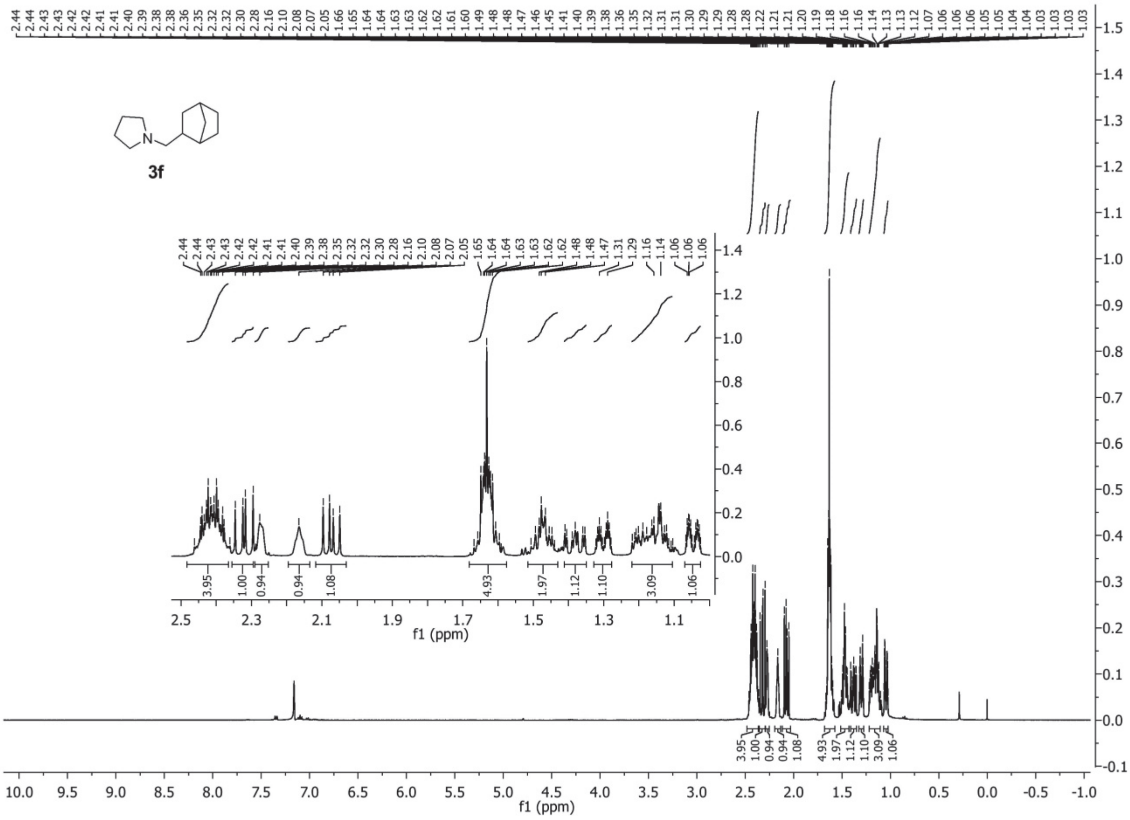
3f
f1 (ppm)

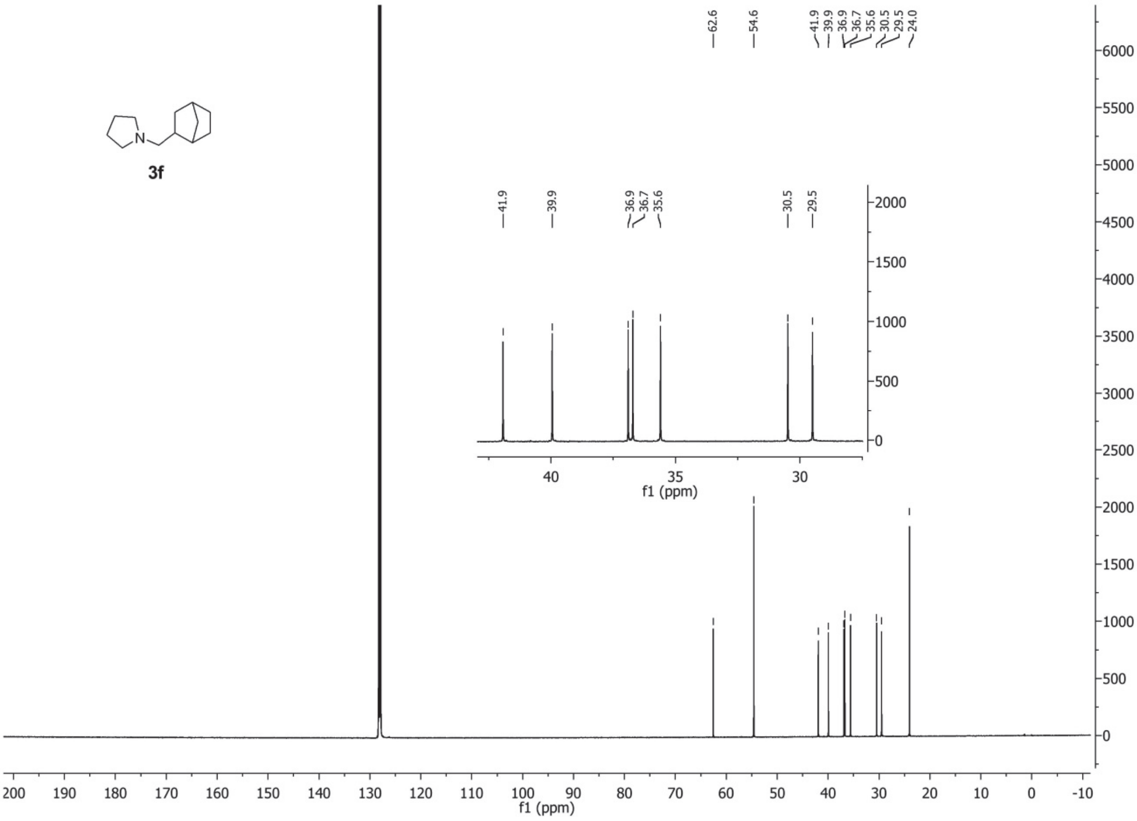
62.6
54.6
41.9
39.9
36.9
36.7
35.6
30.5
29.5
24.0
3f
f1 (ppm)

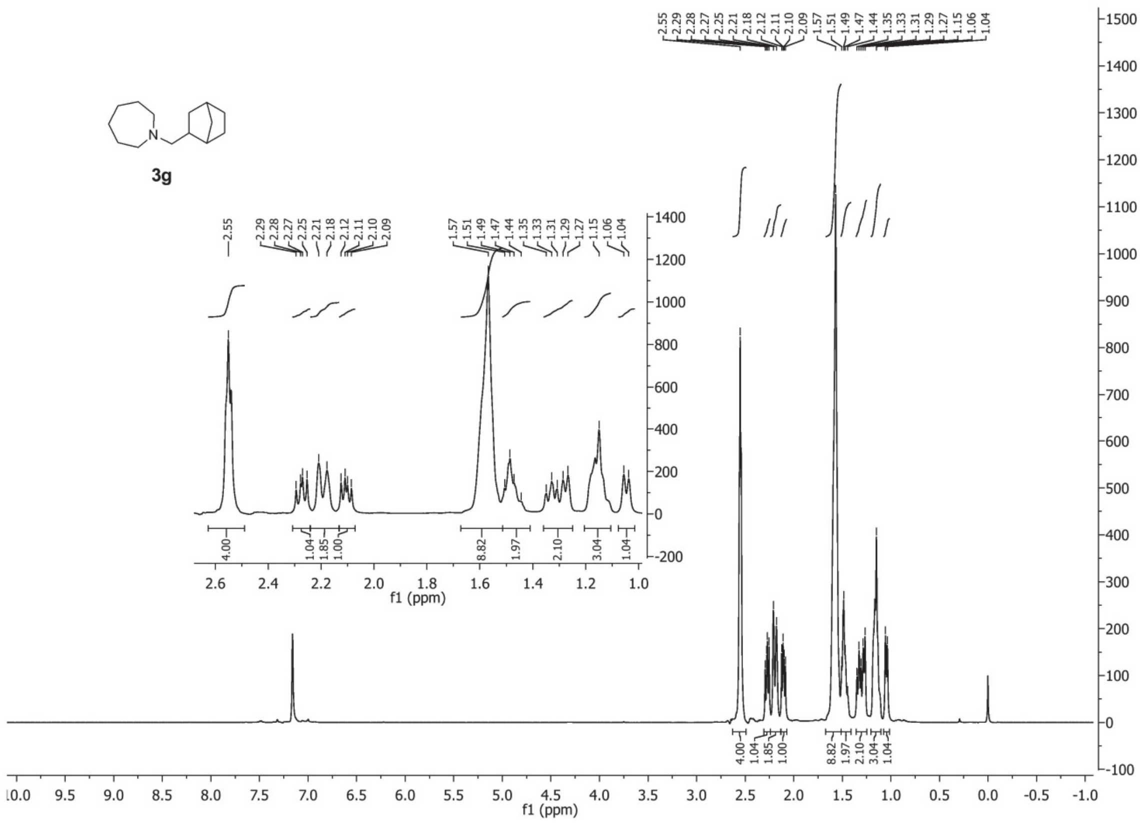

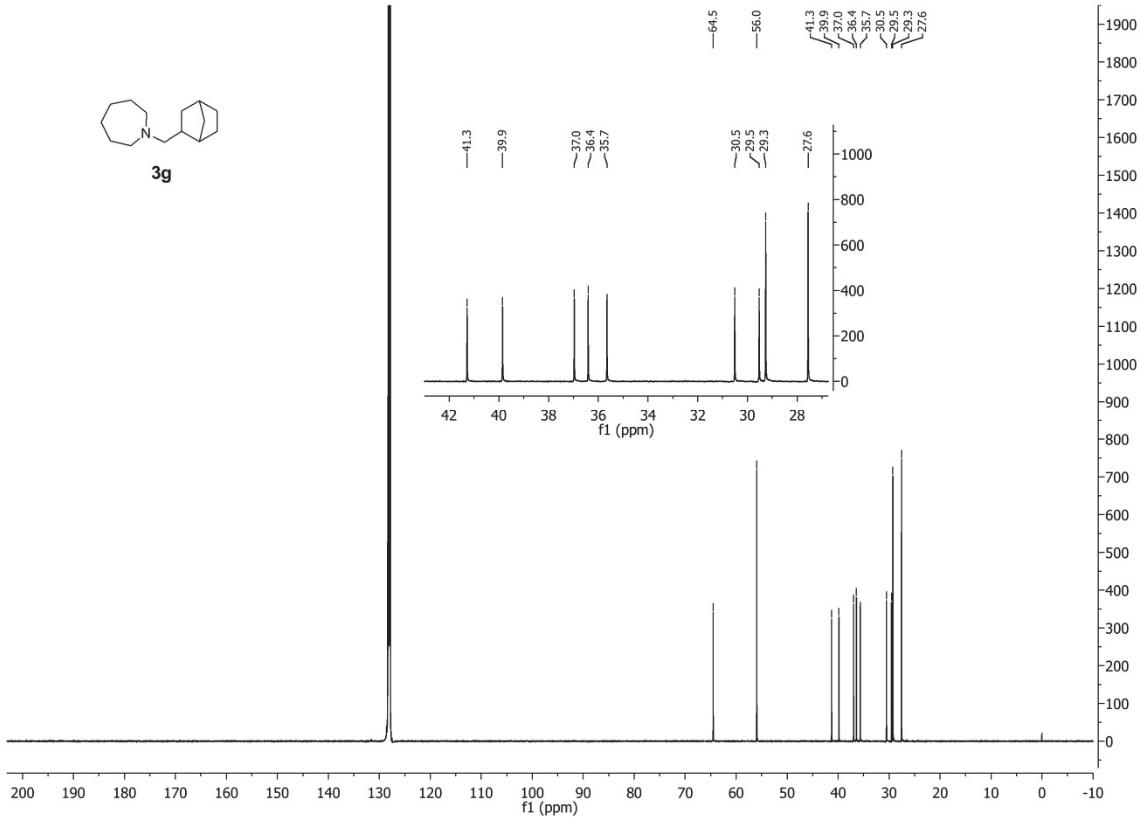

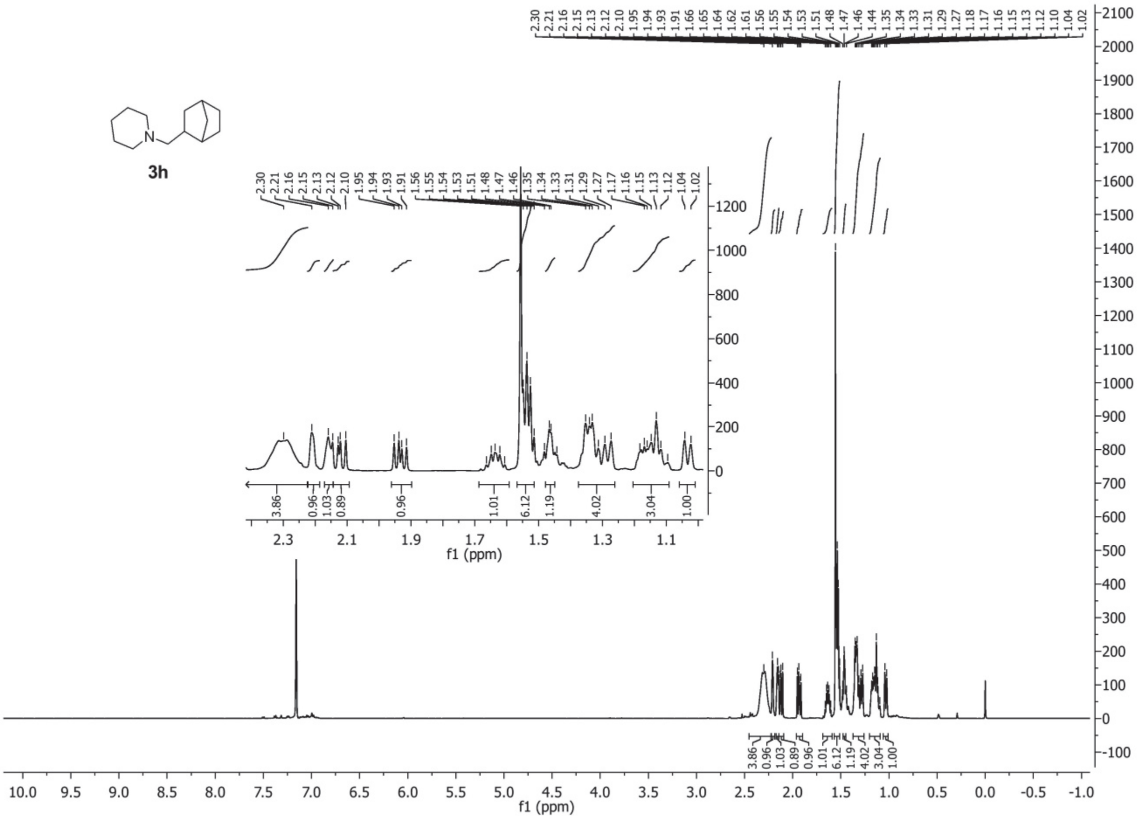
3h
f1 (ppm)

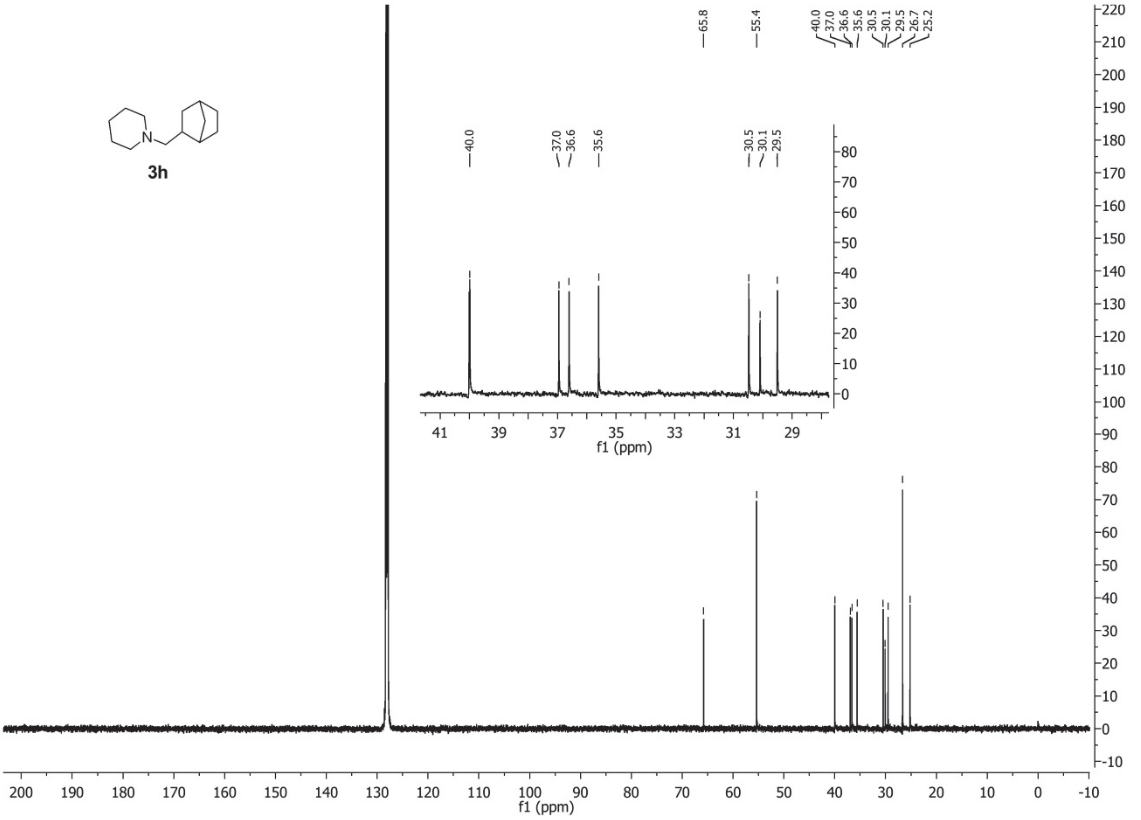
3h
65.8
55.4
40.0
37.0
36.6
35.6
30.5
30.1
29.5
26.7
25.2
f1 (ppm)

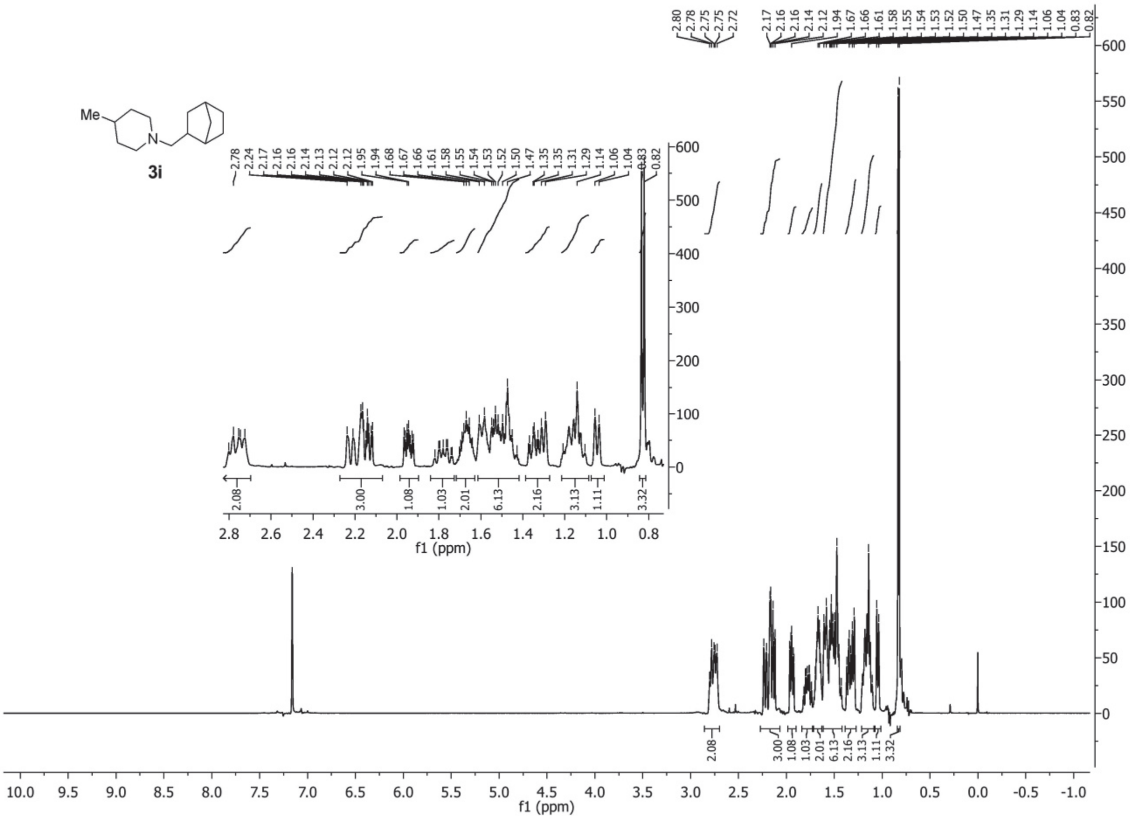
Me
N
3i
f1 (ppm)

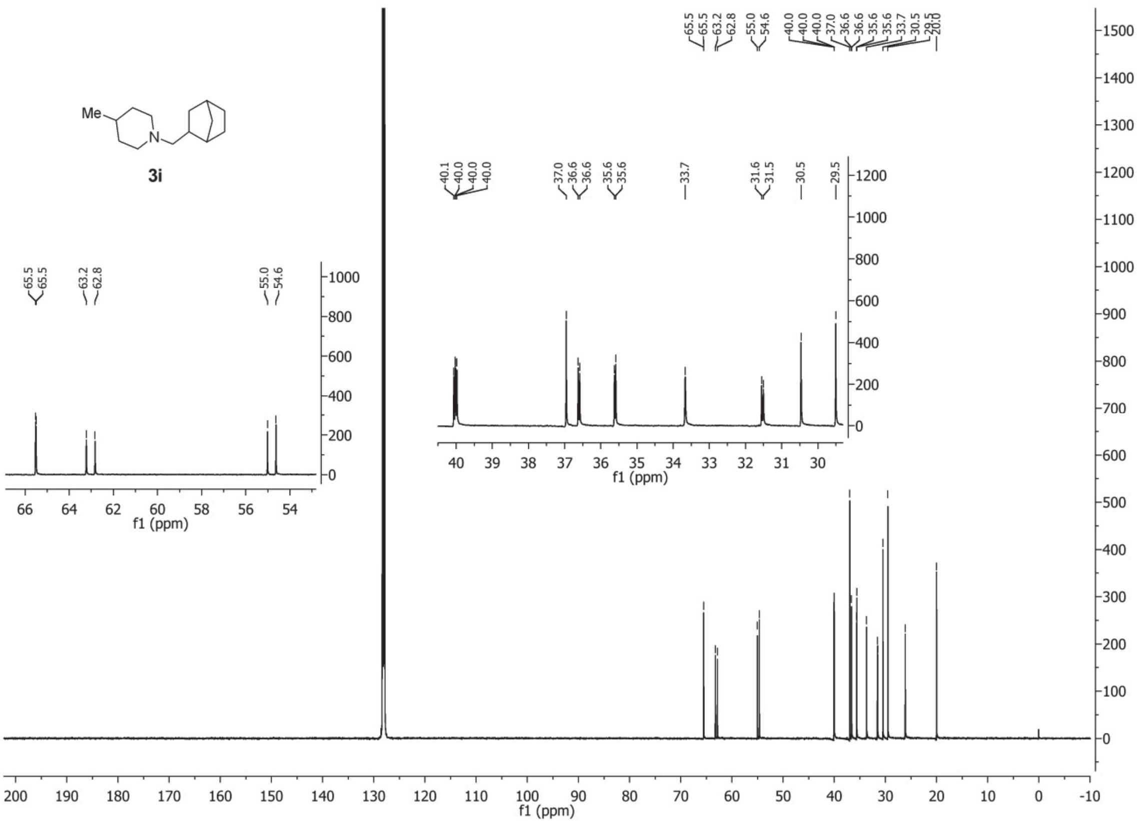
Me
N
3i
f1 (ppm)

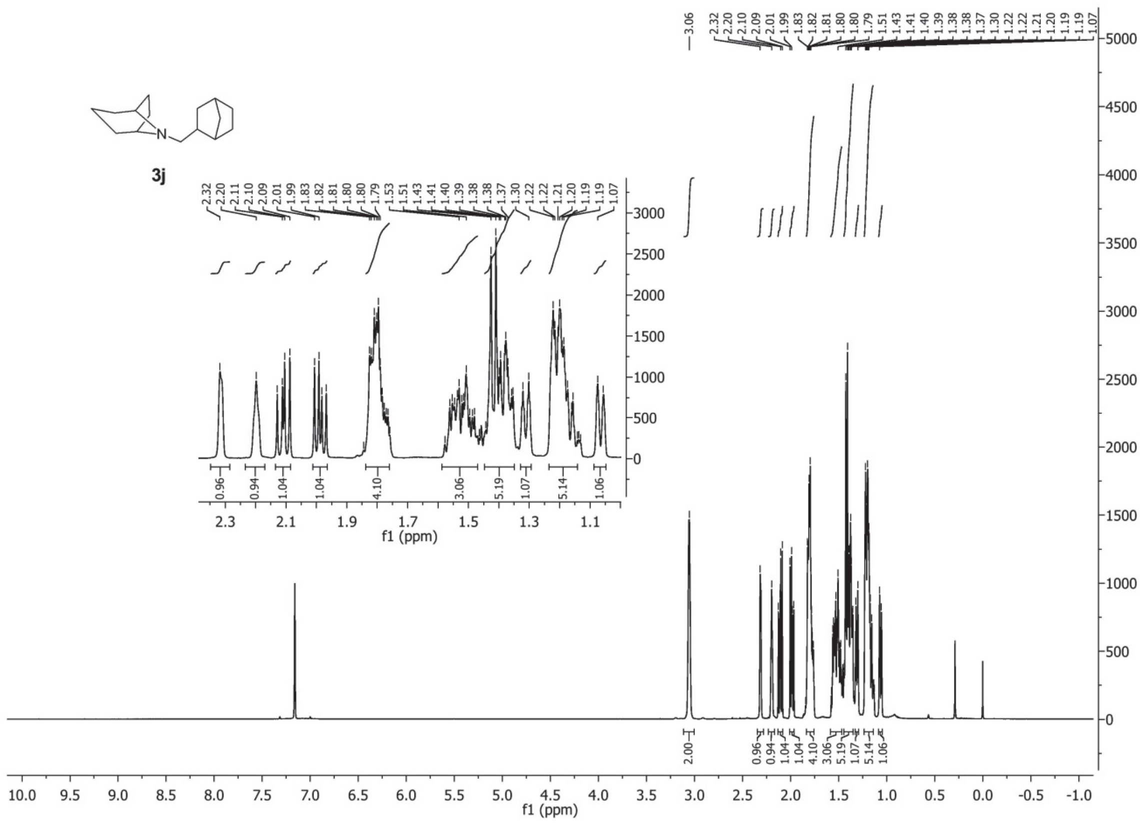

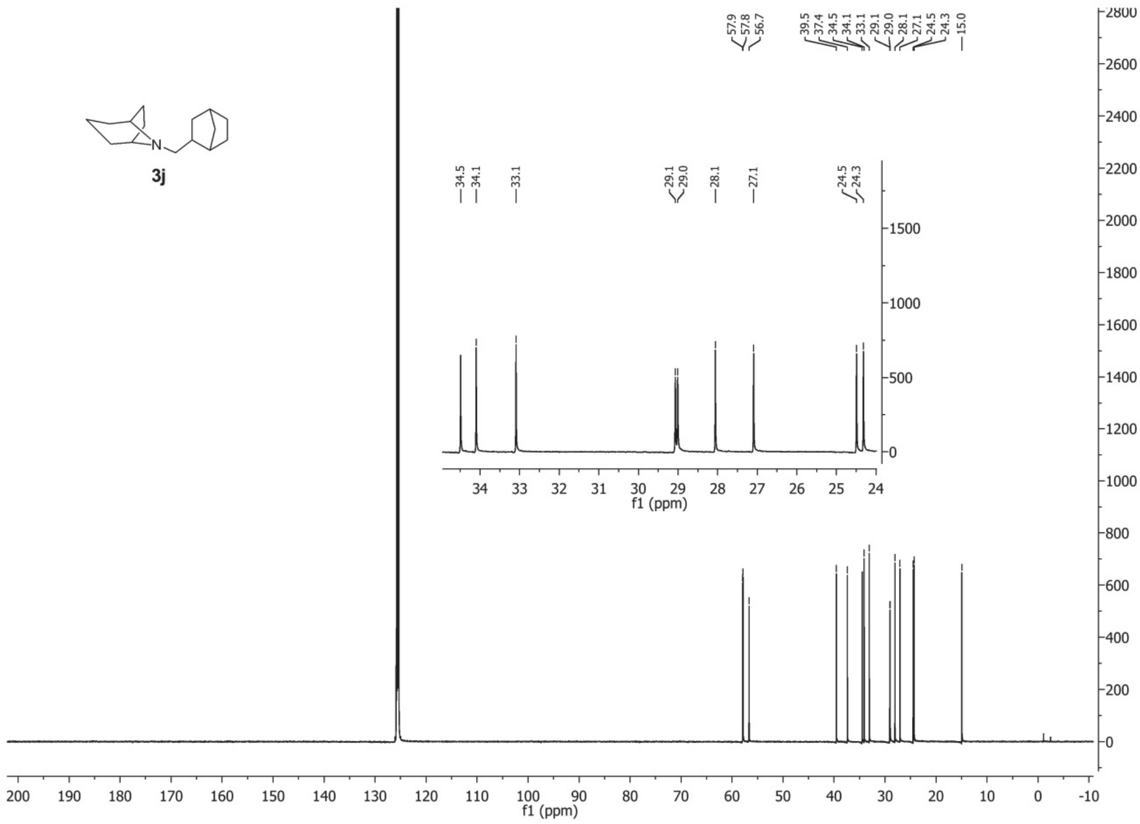

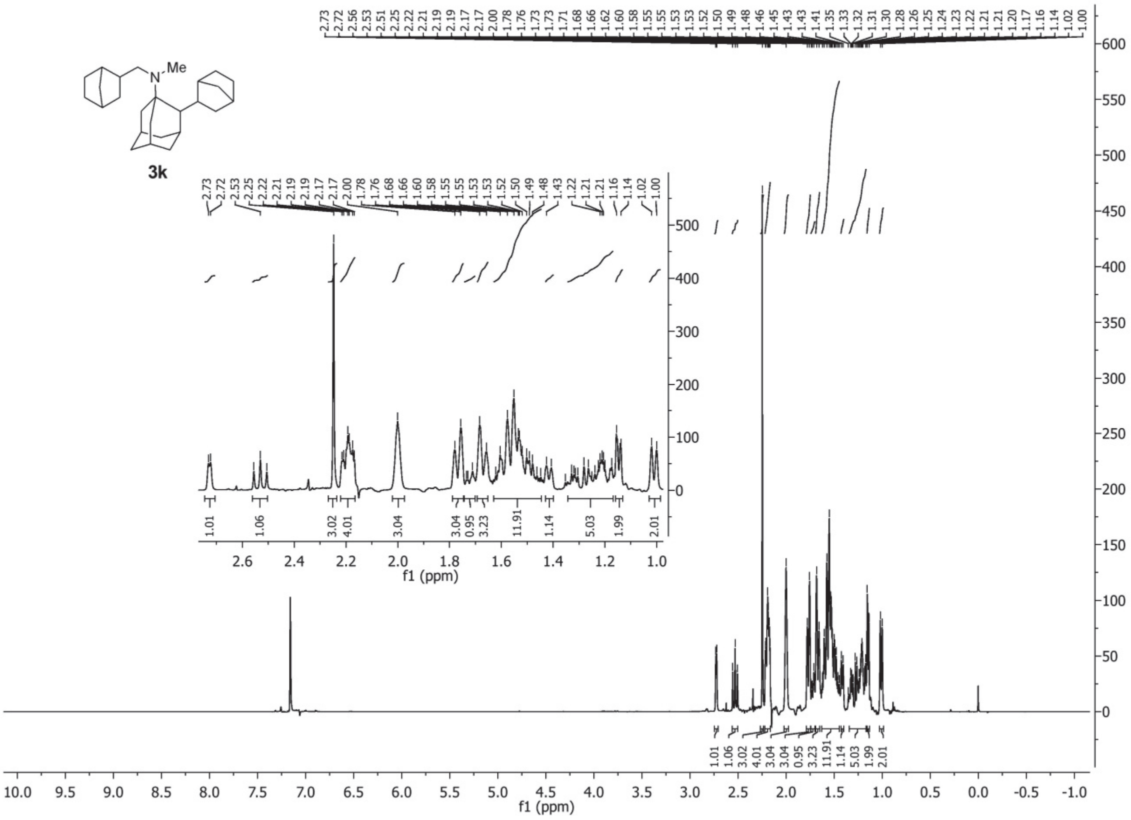
Me
3k
2.73 2.72 2.56 2.53 2.51 2.25 2.22 2.21 2.19 2.19 2.17 2.17 2.00 1.78 1.76 1.73 1.73 1.71 1.68 1.66 1.62 1.60 1.58 1.55 1.55 1.53 1.53 1.52 1.50 1.49 1.48 1.46 1.45 1.43 1.43 1.41 1.35 1.33 1.32 1.31 1.30 1.28 1.26 1.25 1.24 1.23 1.22 1.22 1.21 1.21 1.20 1.17 1.16 1.14 1.02 1.00
1.01 1.06 3.02 4.01 3.04 3.04 0.95 3.23 11.91 1.14 5.03 1.99 2.01
f1 (ppm)

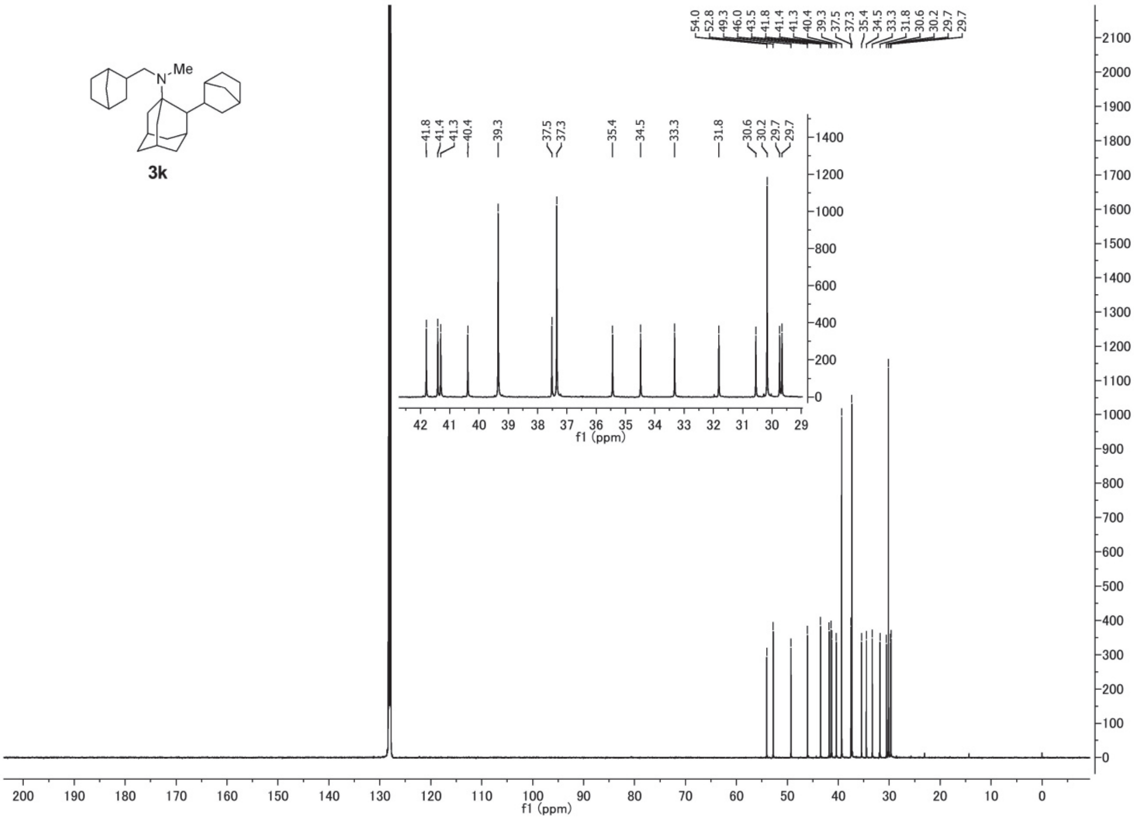
Me
3k
54.0 52.8 49.3 46.0 43.5 41.8 41.4 41.3 40.4 39.3 37.5 37.3 35.4 34.5 33.3 31.8 30.6 30.2 29.7 29.7
f1 (ppm)

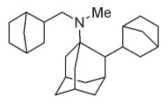

3k

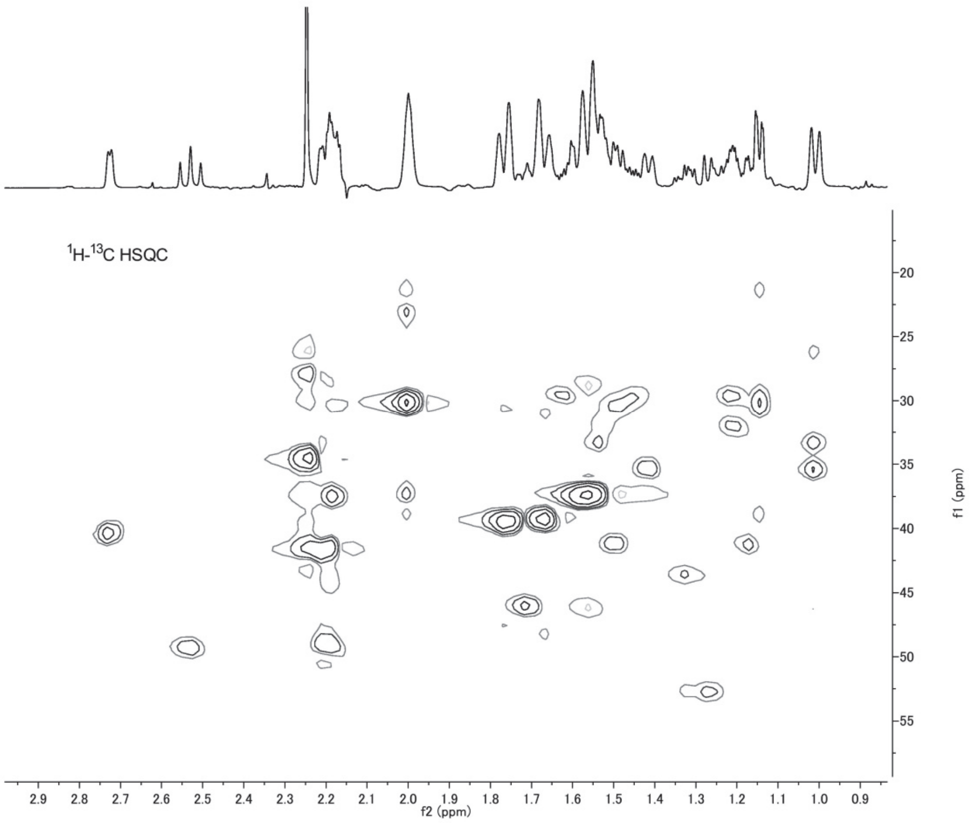

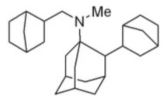

3k

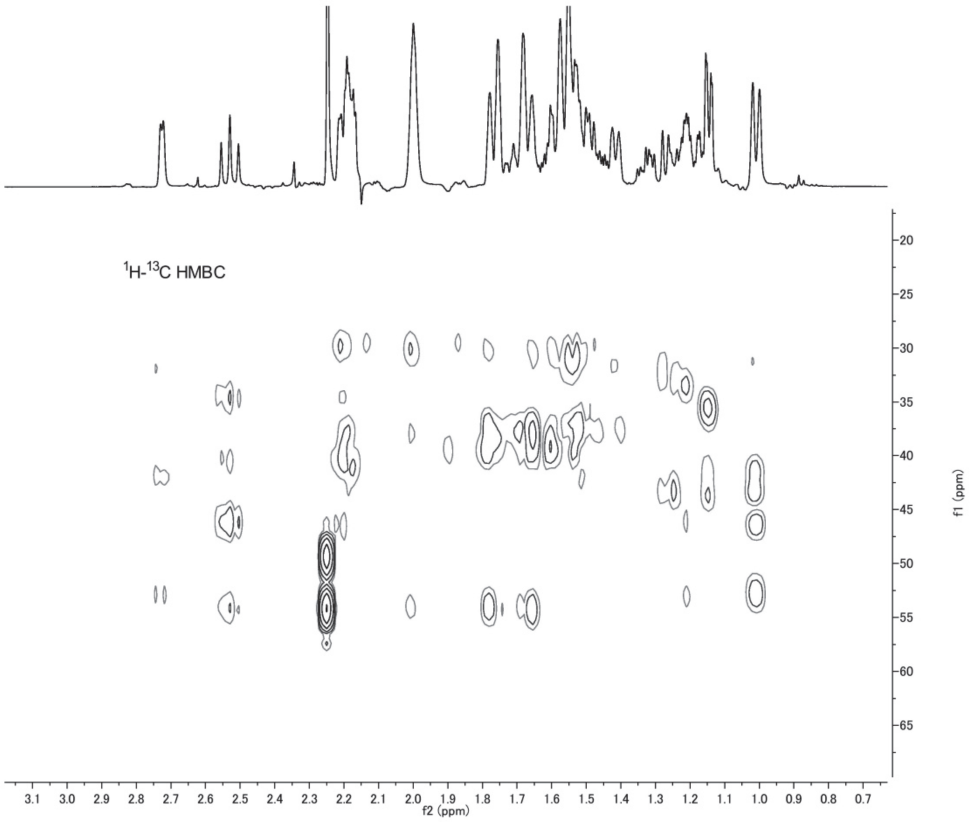

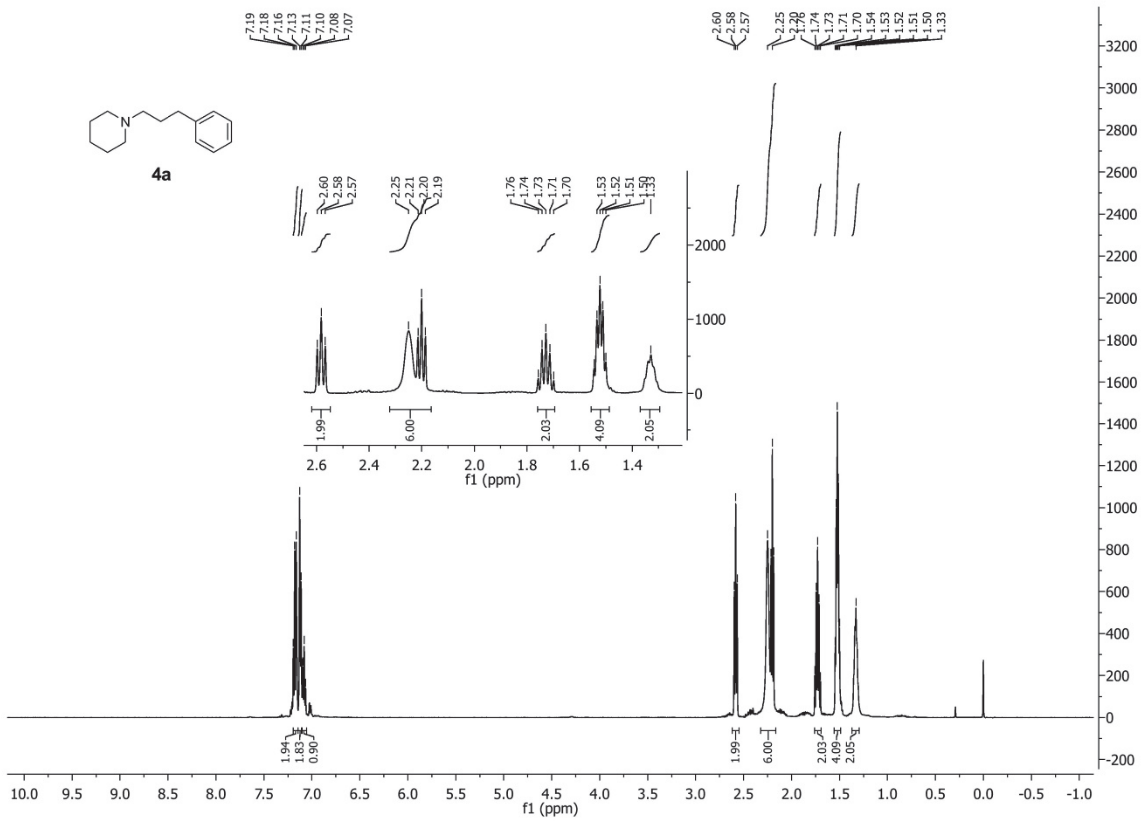
4a
f1 (ppm)

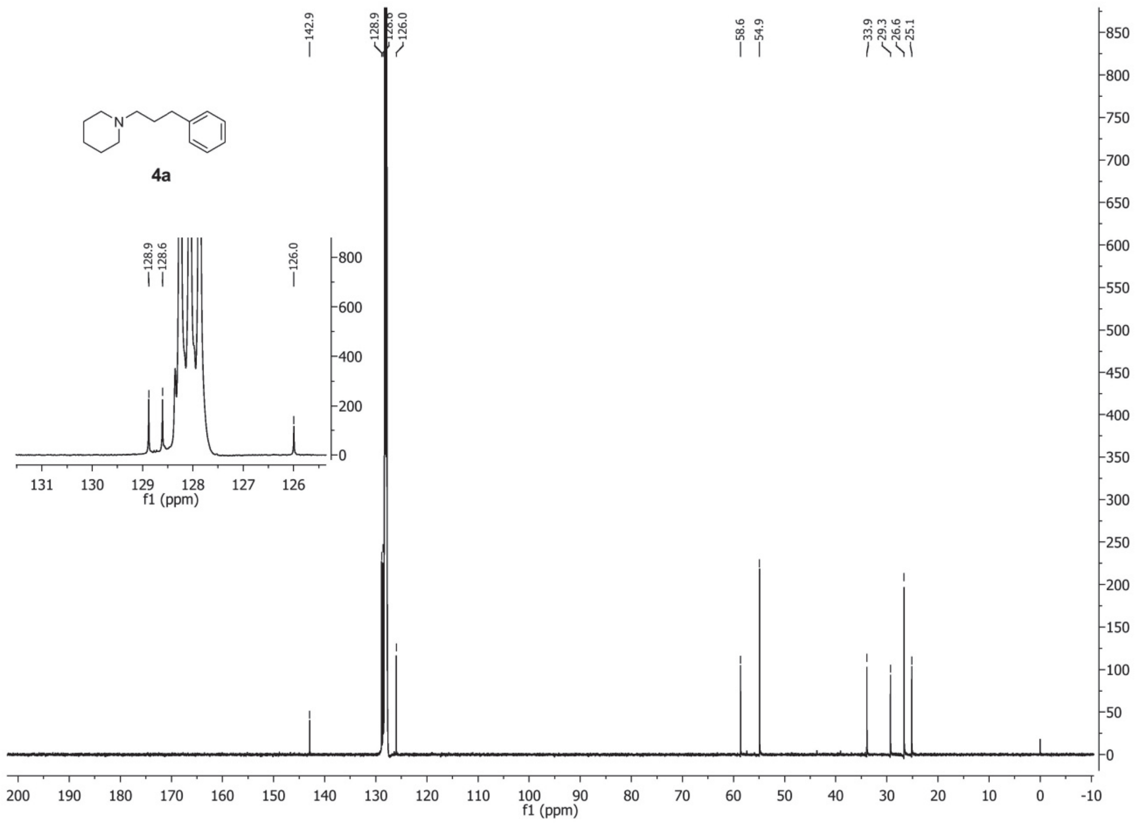
4a
f1 (ppm)

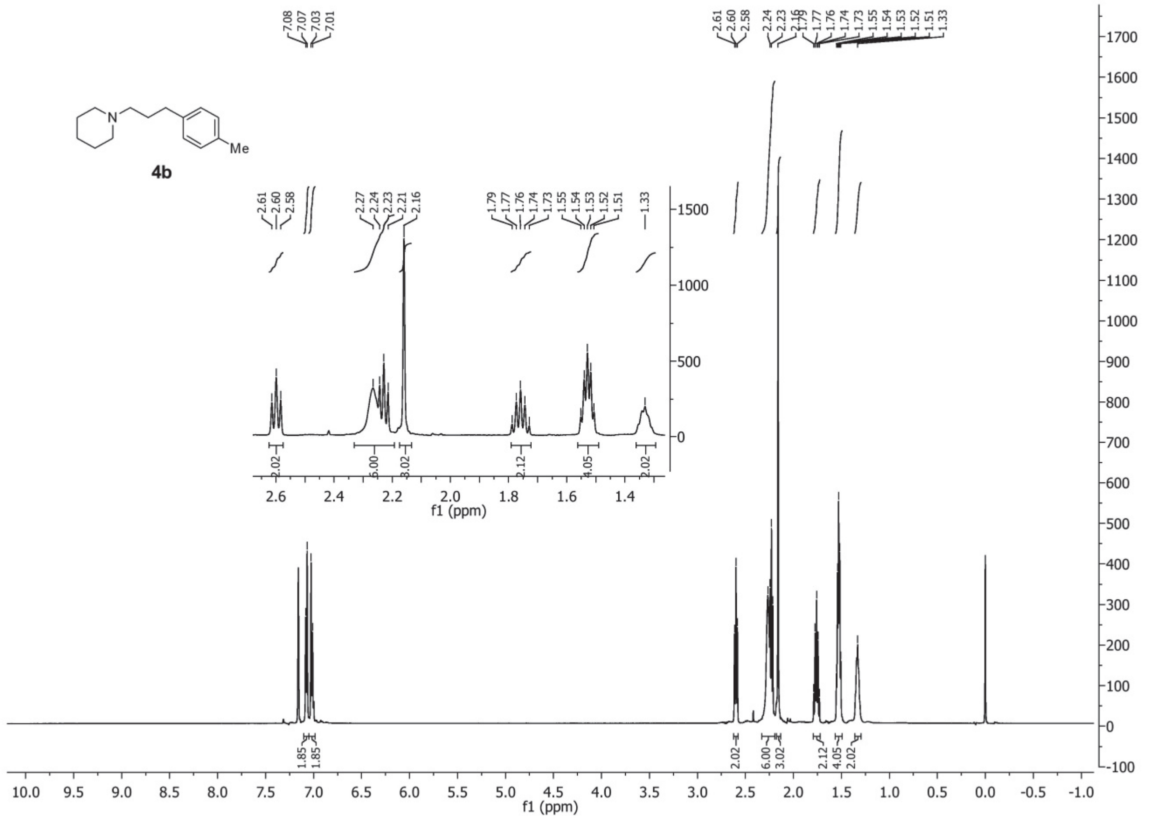
Me
4b
f1 (ppm)

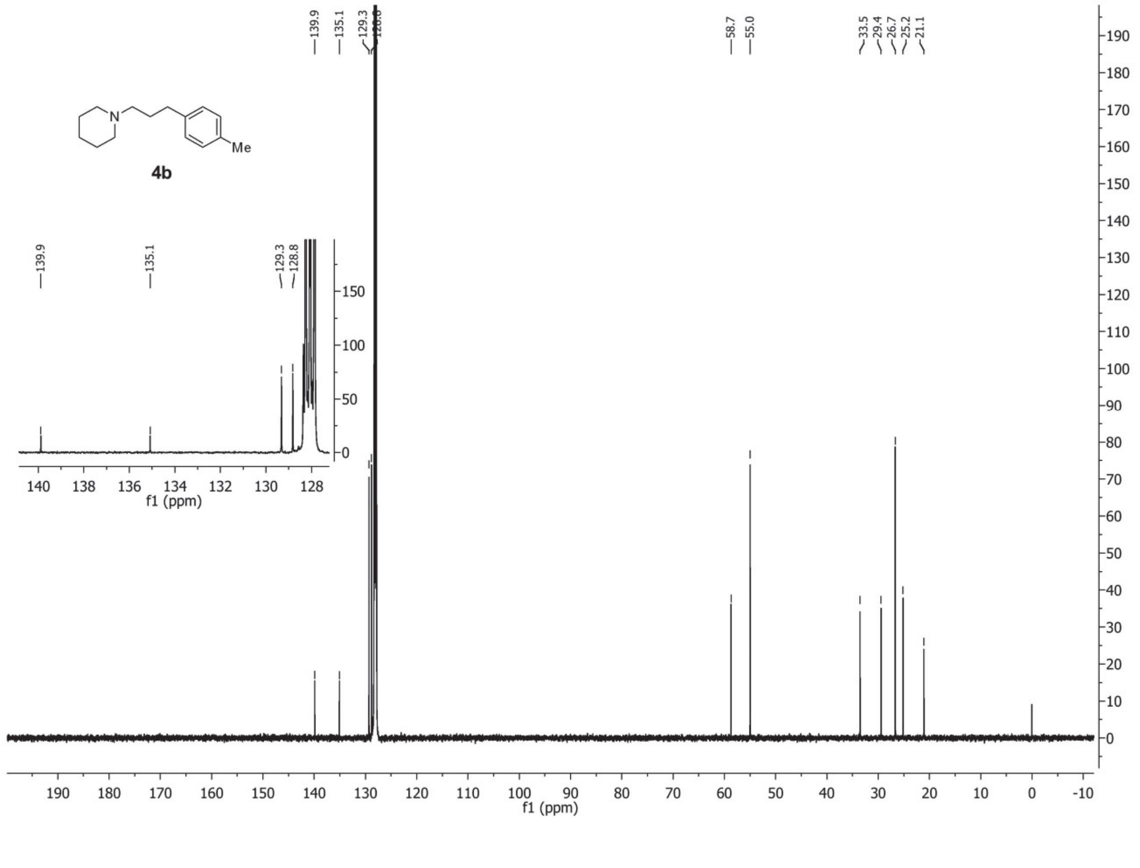
Me
4b
f1 (ppm)

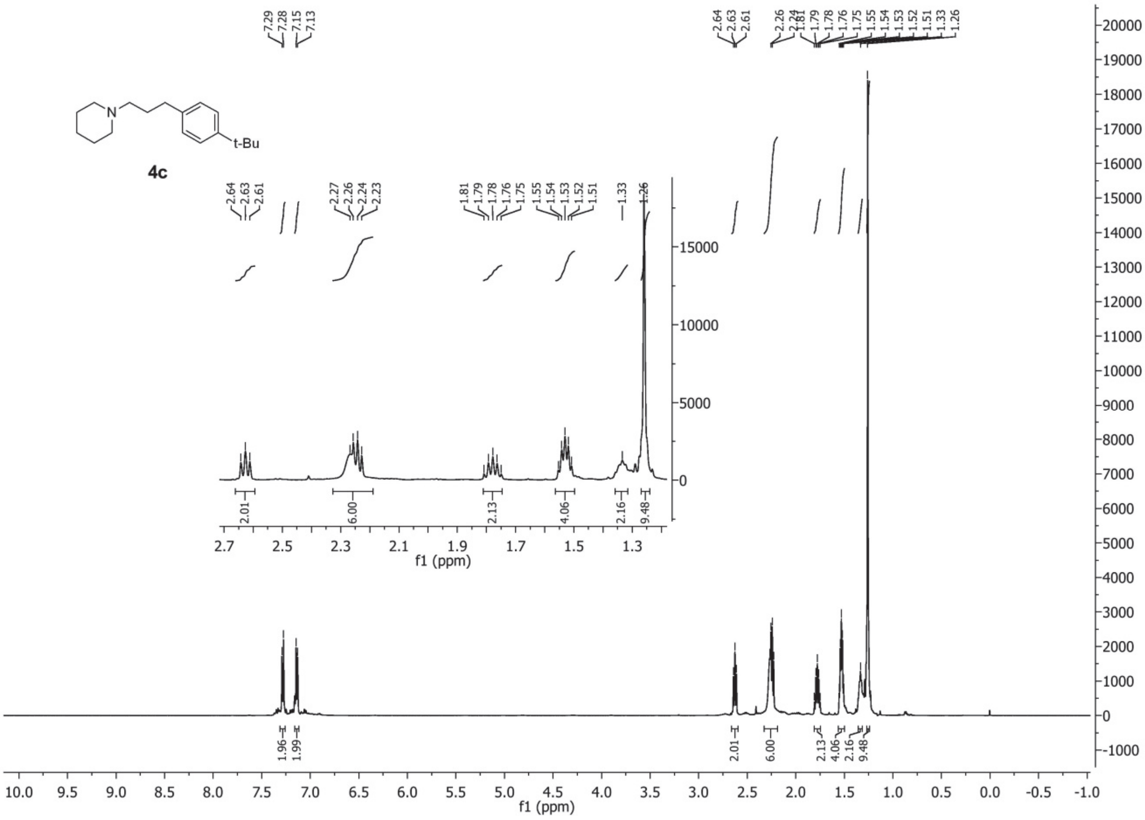
7.29
7.28
7.15
7.13
2.64
2.63
2.61
2.26
2.24
1.81
1.79
1.78
1.76
1.75
1.55
1.54
1.53
1.52
1.51
1.33
1.26
t-Bu
4c
1.96
1.99
2.01
6.00
2.13
4.06
2.16
9.48
f1 (ppm)

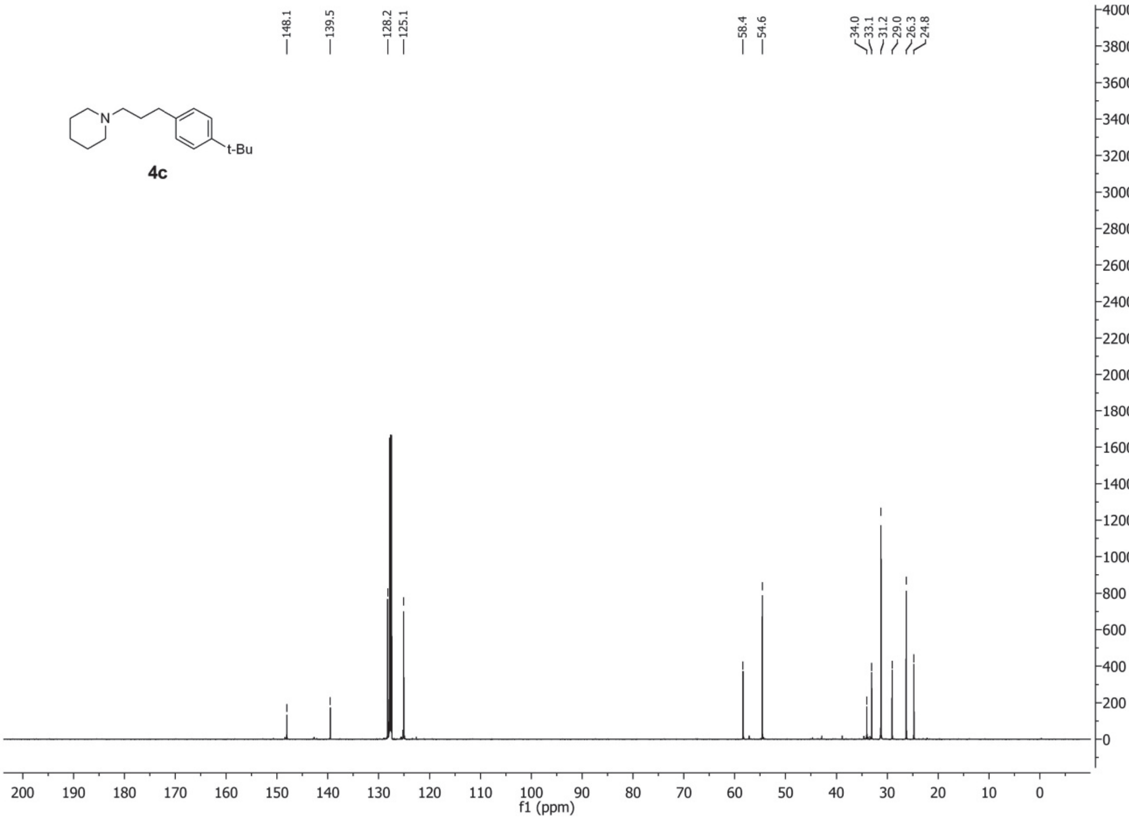
148.1
139.5
128.2
125.1
58.4
54.6
34.0
33.1
31.2
29.0
26.3
24.8
t-Bu
4c
f1 (ppm)

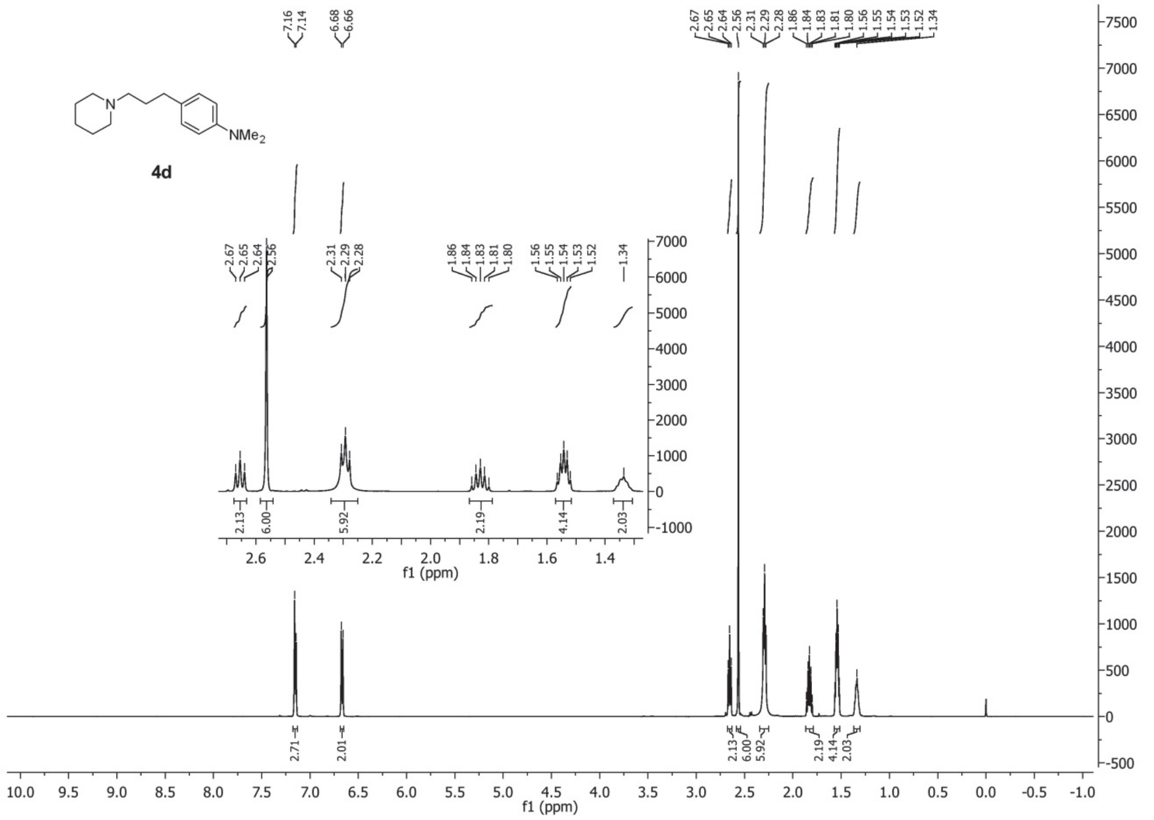
NMe2
4d
7.16
7.14
6.68
6.66
2.67
2.65
2.64
2.56
2.31
2.29
2.28
1.86
1.84
1.83
1.81
1.80
1.56
1.55
1.54
1.53
1.52
1.34
2.71
2.01
2.13
6.00
5.92
2.19
4.14
2.03
f1 (ppm)

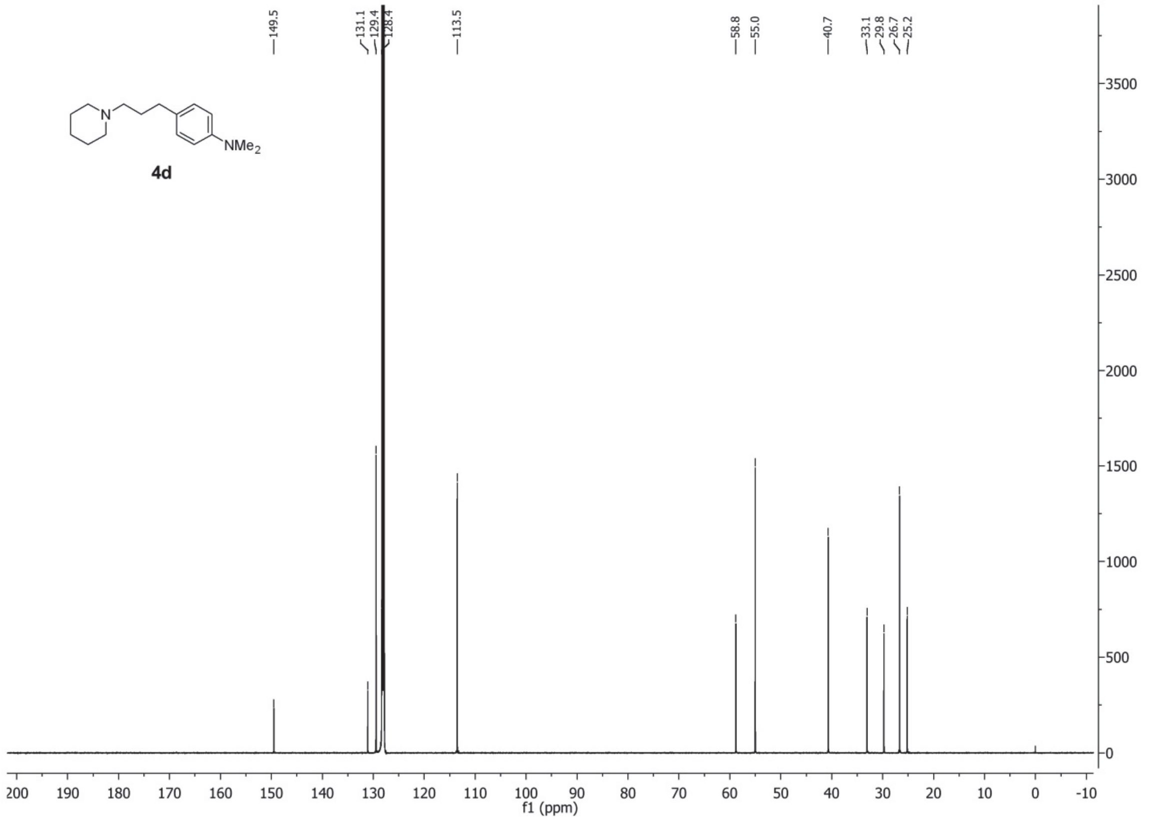
NMe2
4d
149.5
131.1
129.4
128.4
113.5
58.8
55.0
40.7
33.1
29.8
26.7
25.2
f1 (ppm)

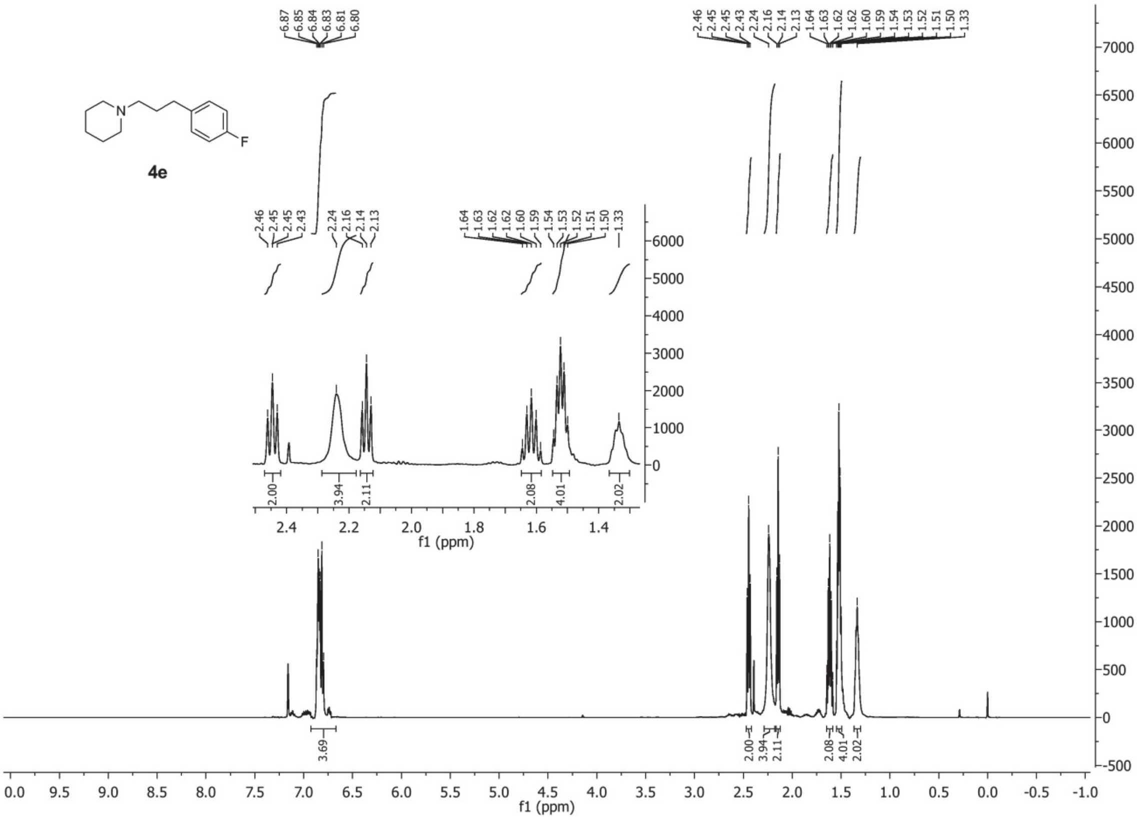
4e
f1 (ppm)

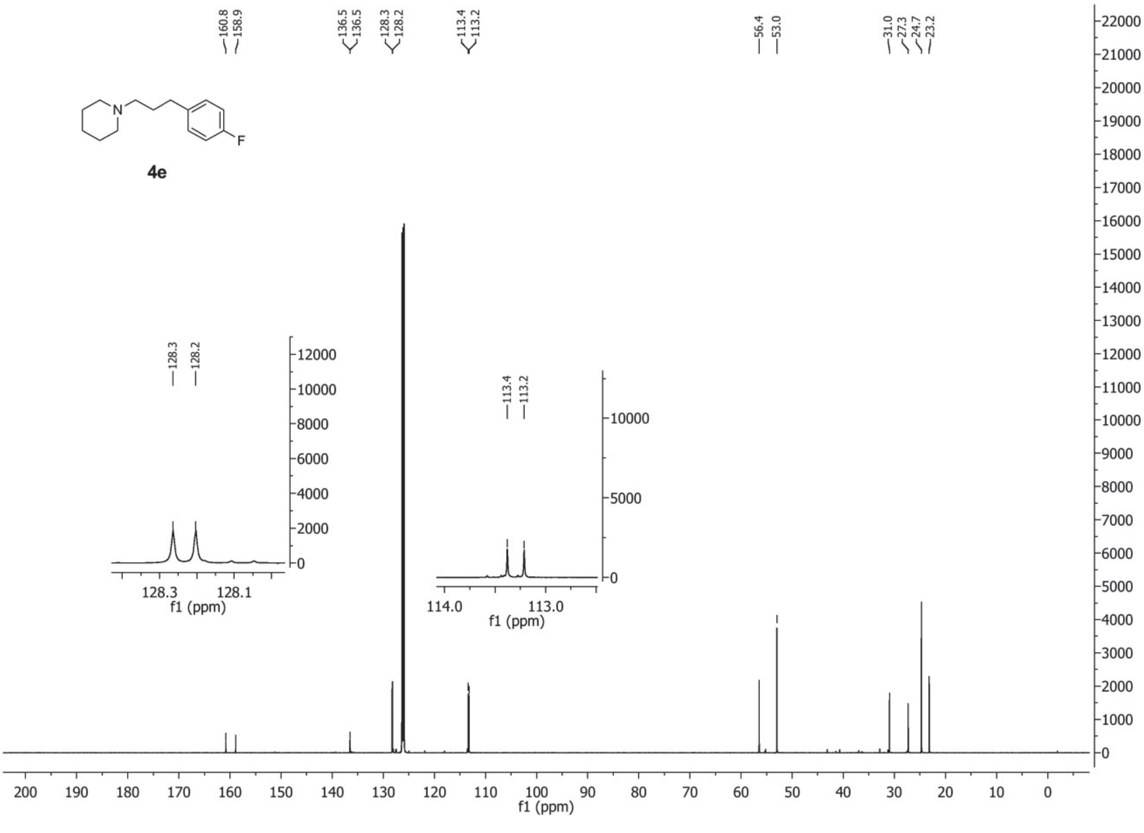
4e
f1 (ppm)

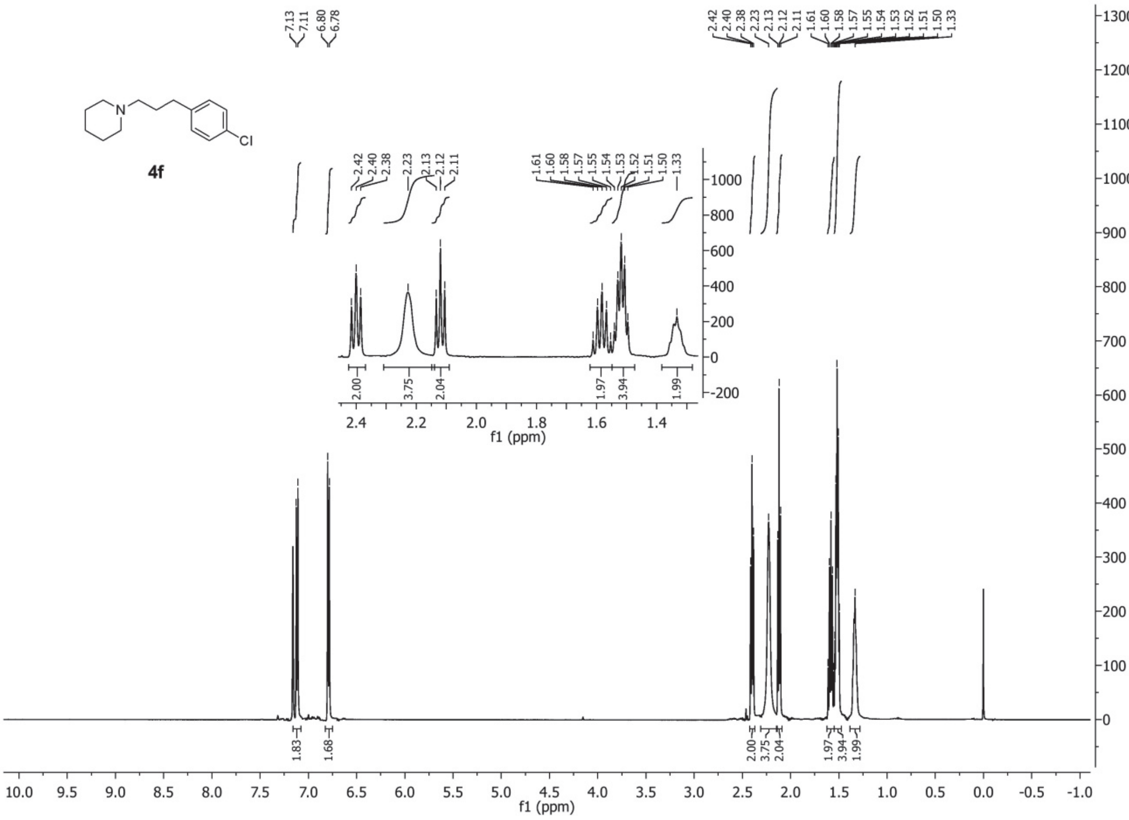
4f
7.13
7.11
6.80
6.78
2.42
2.40
2.38
2.23
2.13
2.12
2.11
1.61
1.60
1.58
1.57
1.55
1.54
1.53
1.52
1.51
1.50
1.33
f1 (ppm)

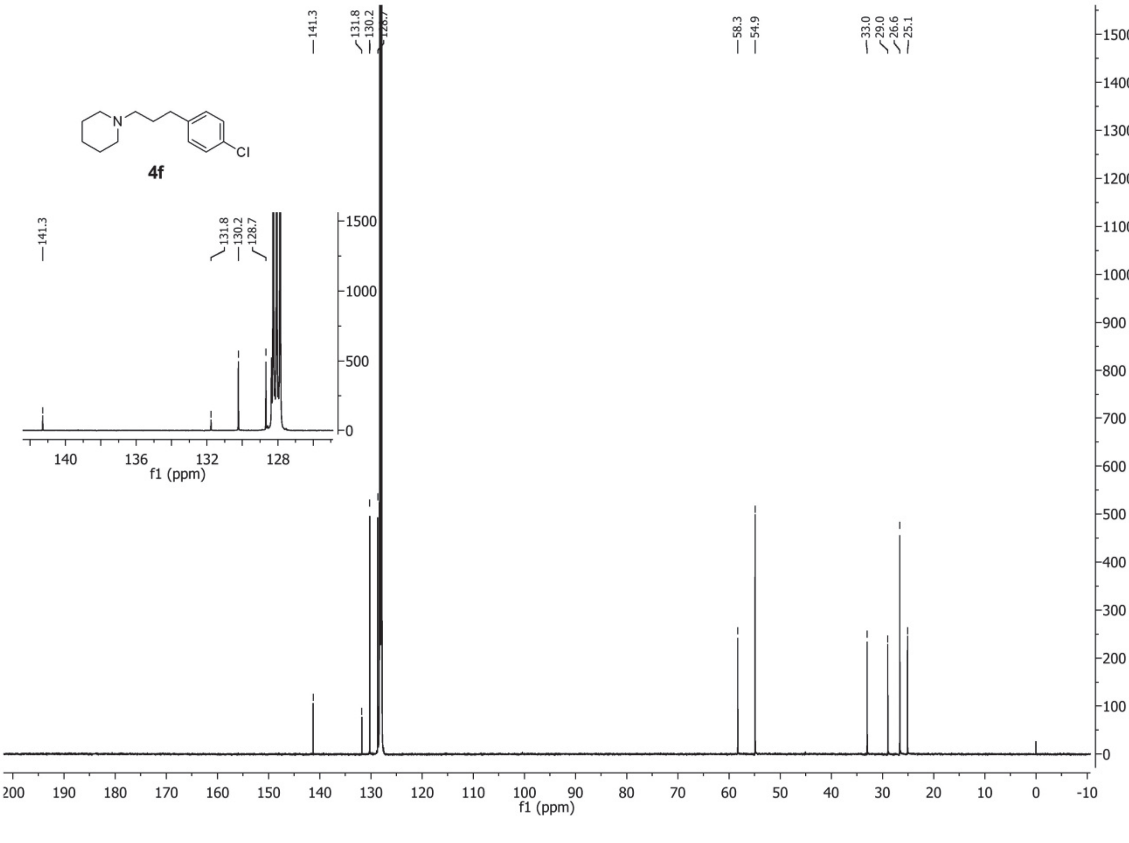
4f
141.3
131.8
130.2
128.7
58.3
54.9
33.0
29.0
26.6
25.1
f1 (ppm)

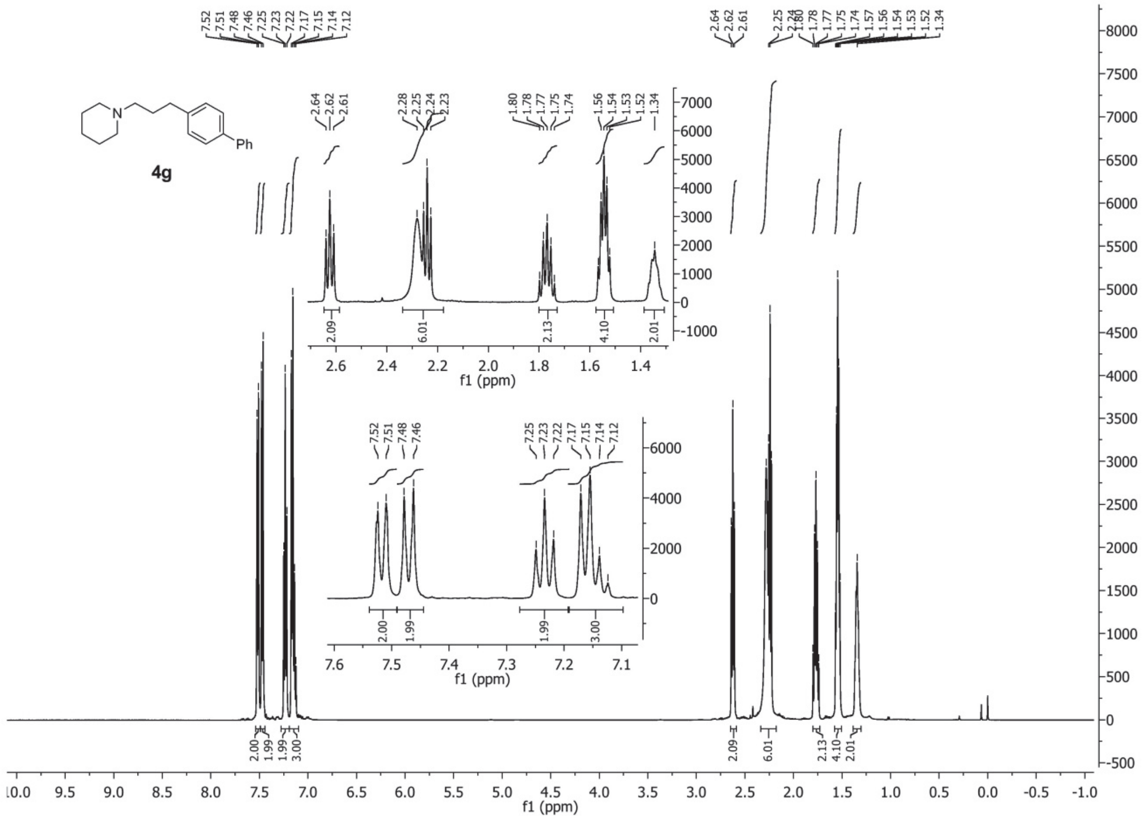
Ph
4g
f1 (ppm)

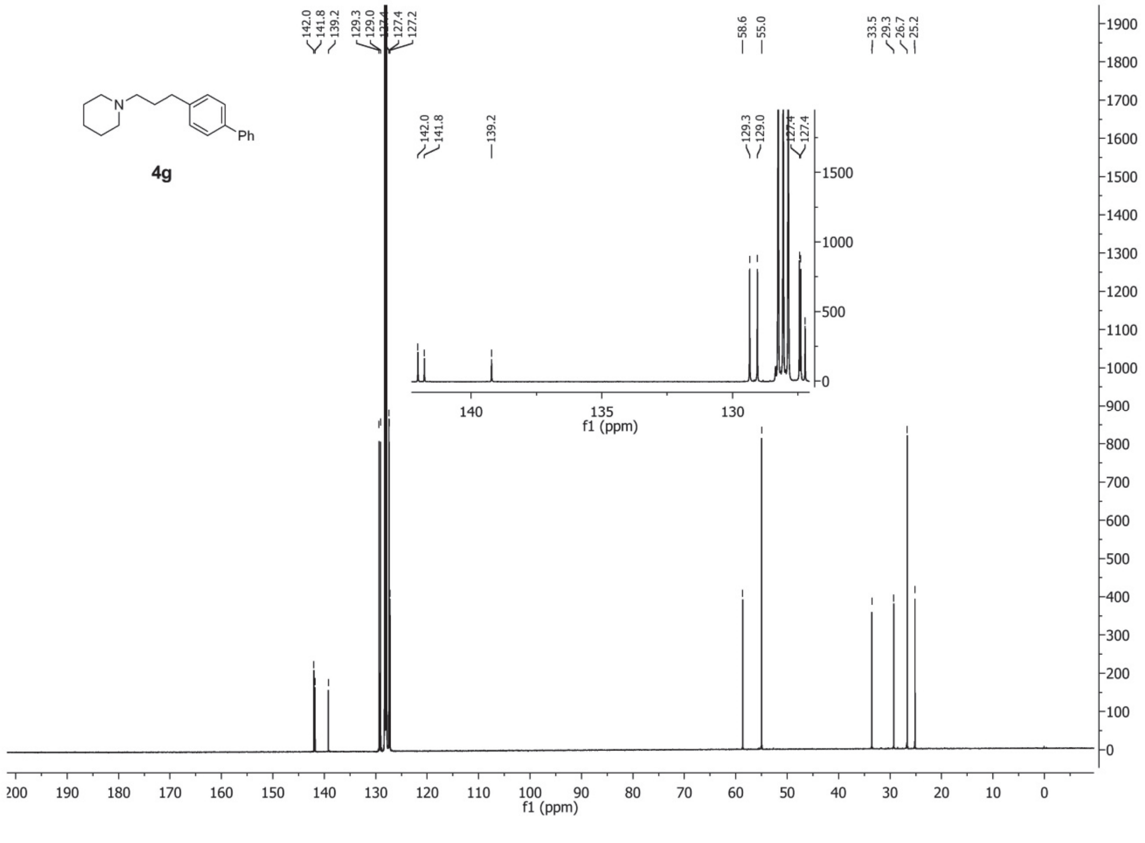
Ph
4g
f1 (ppm)

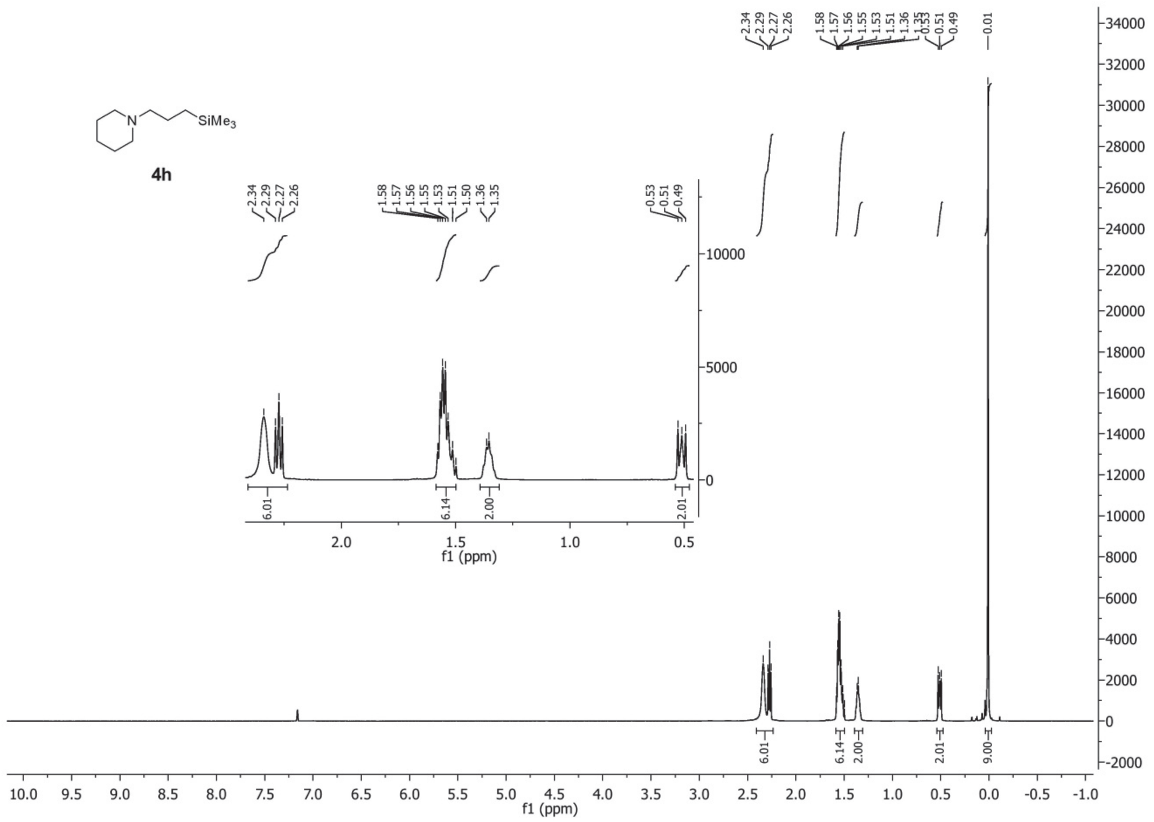
SiMe3
4h
f1 (ppm)

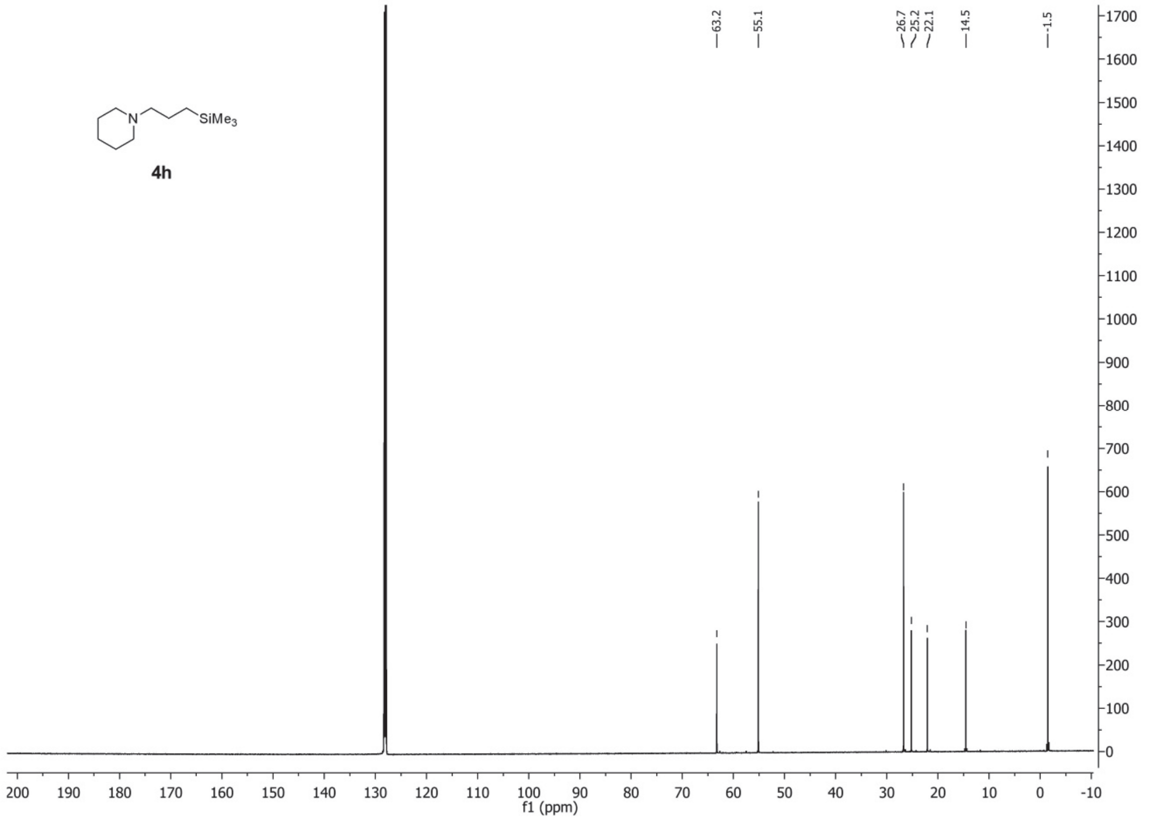
SiMe3
4h
63.2
55.1
26.7
25.2
22.1
14.5
-1.5
f1 (ppm)

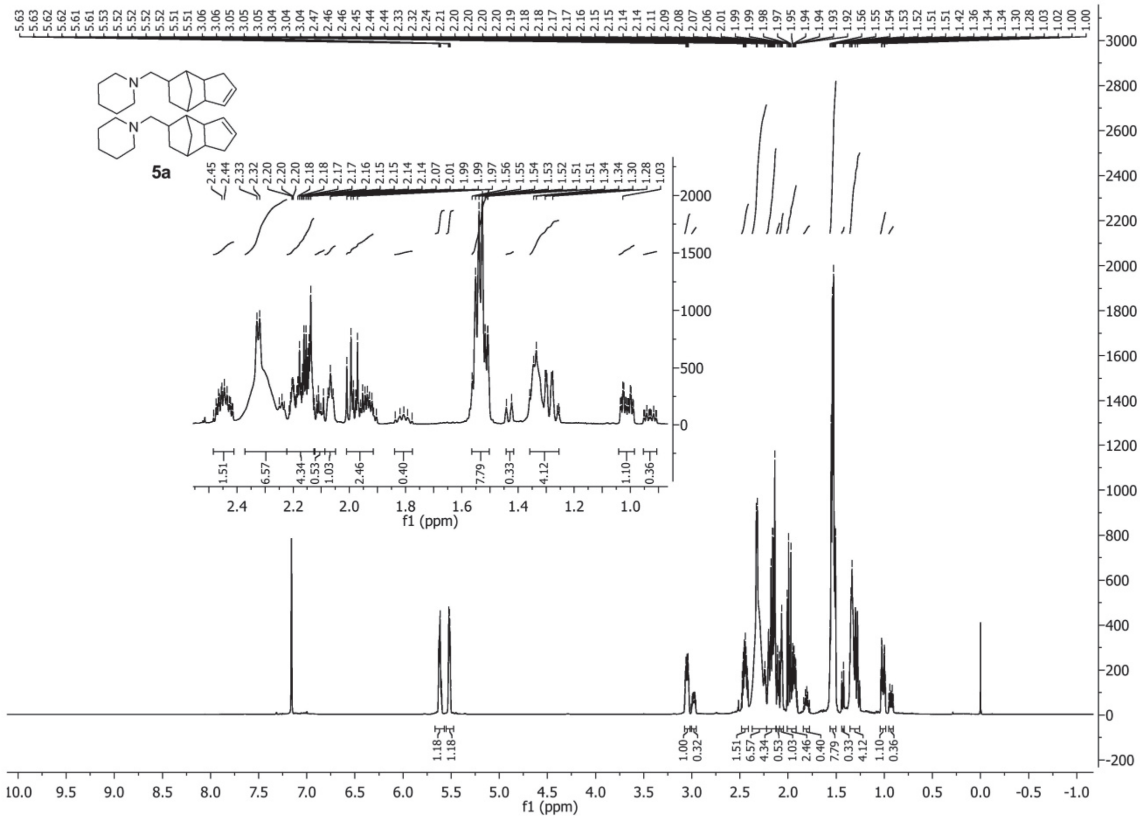
5a
f1 (ppm)

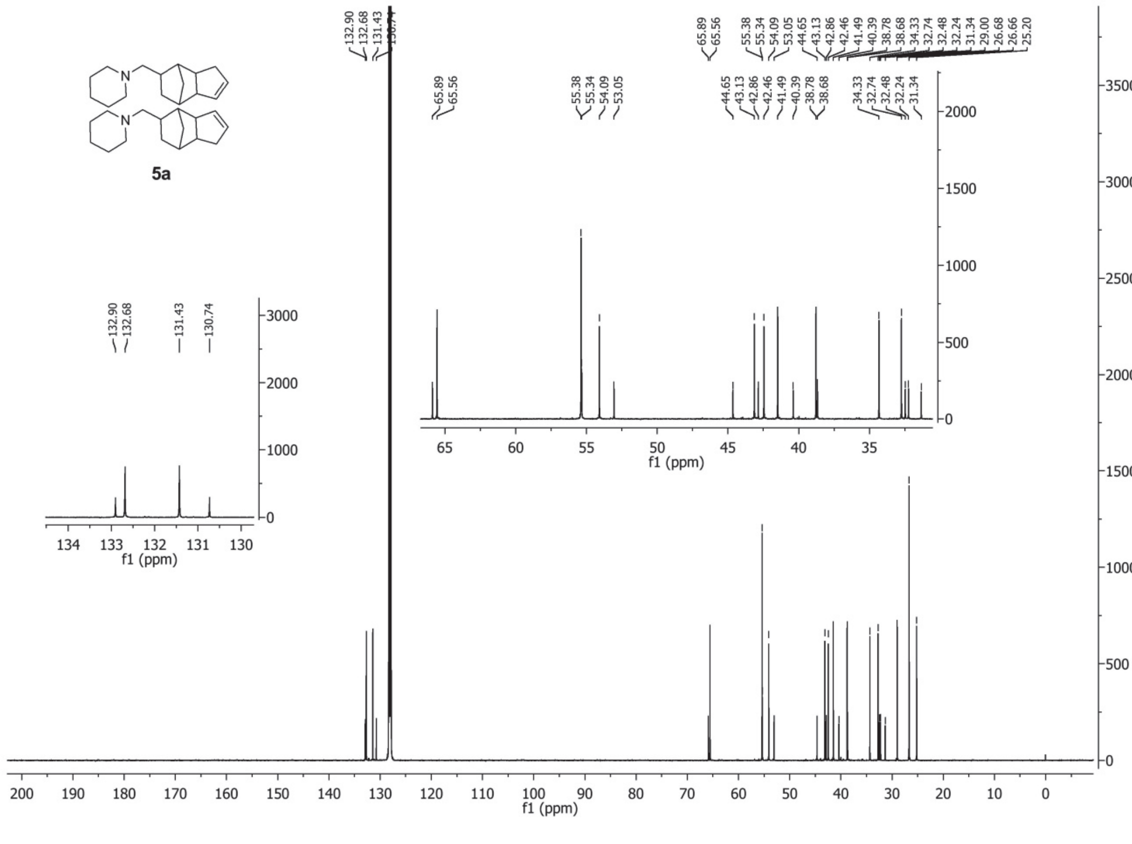
5a
f1 (ppm)

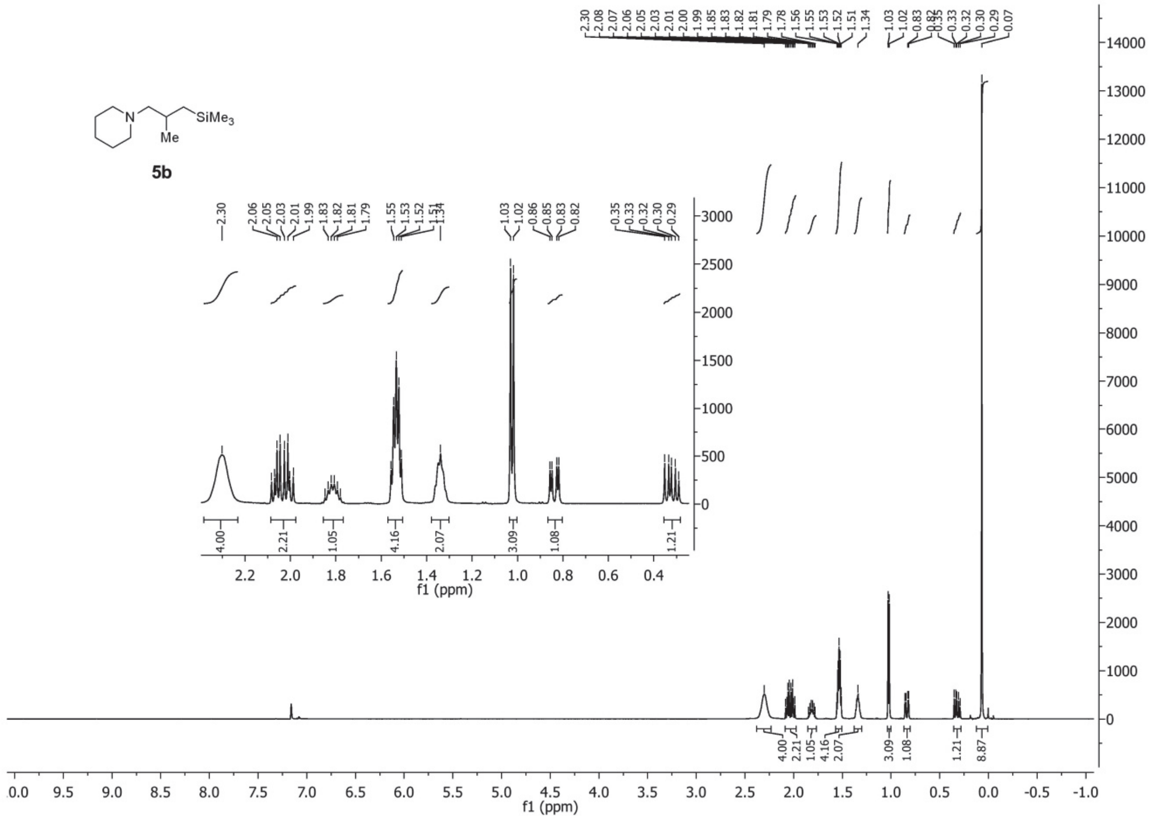
SiMe3
Me
5b
f1 (ppm)

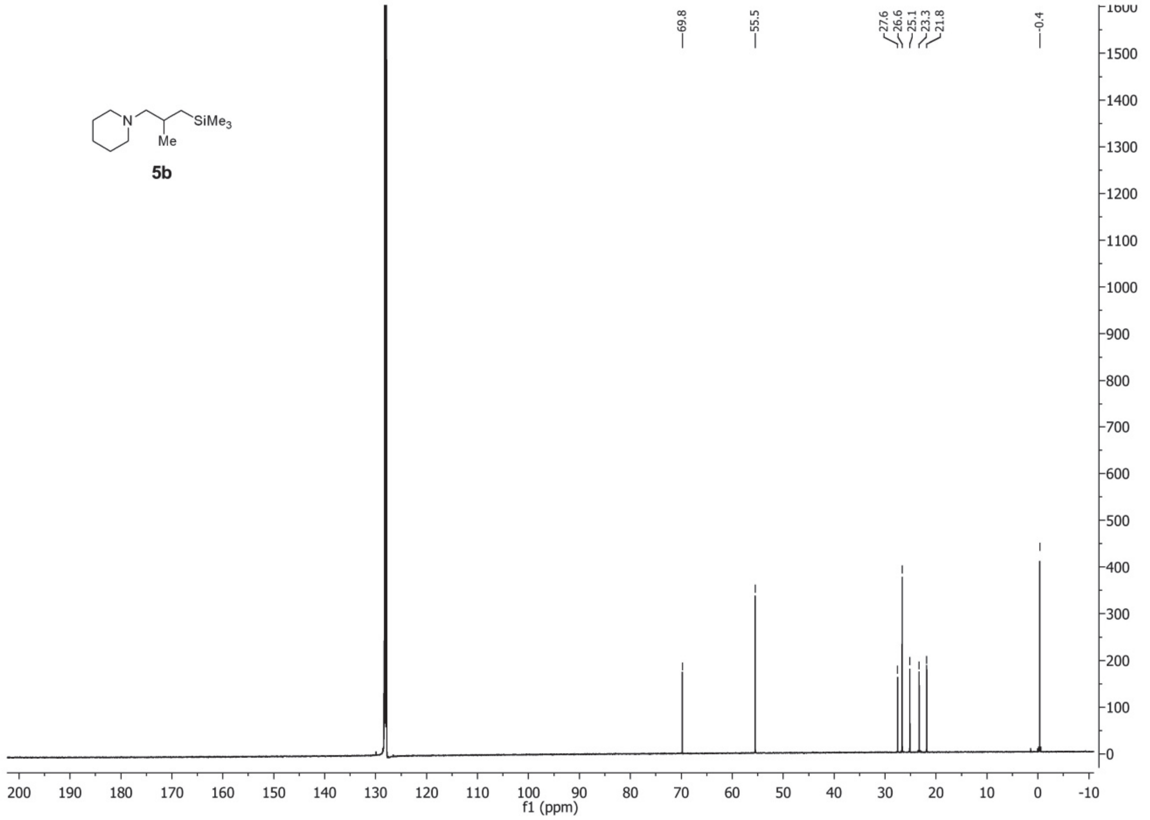
69.8
55.5
27.6
26.6
25.1
23.3
21.8
-0.4
SiMe3
Me
5b
f1 (ppm)

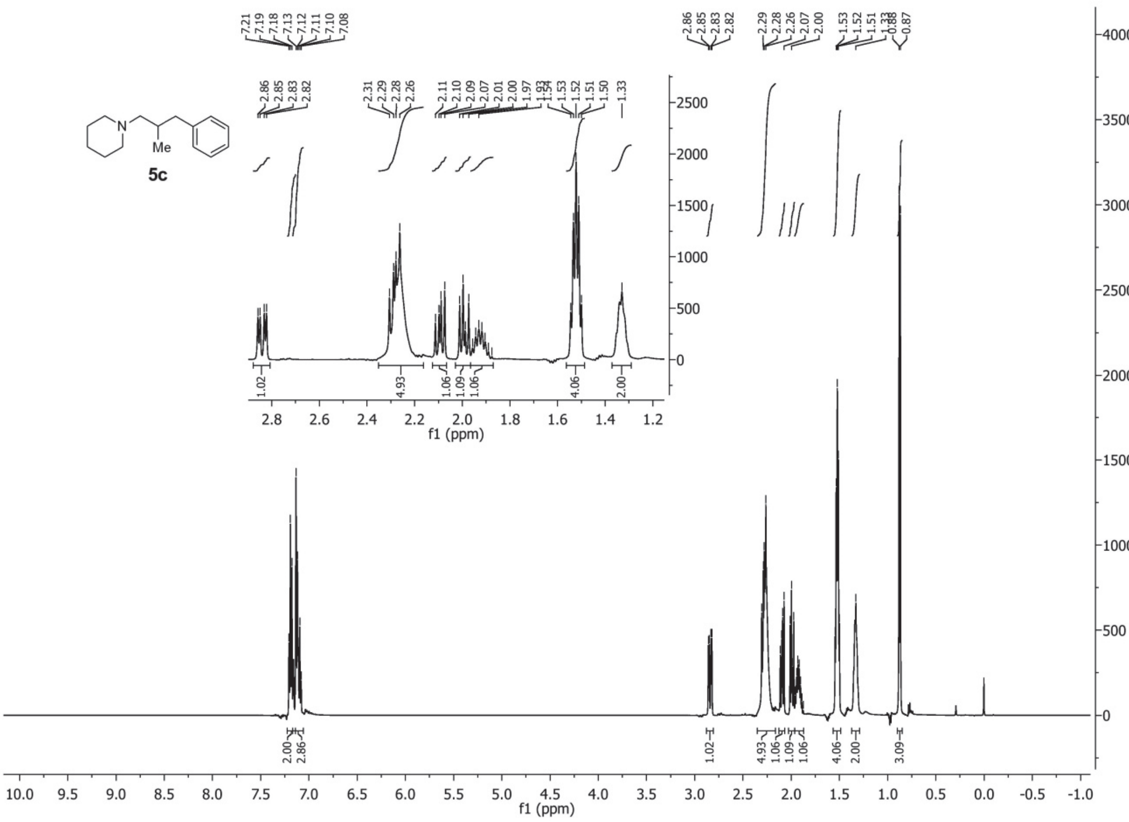
N
Me
5c
f1 (ppm)

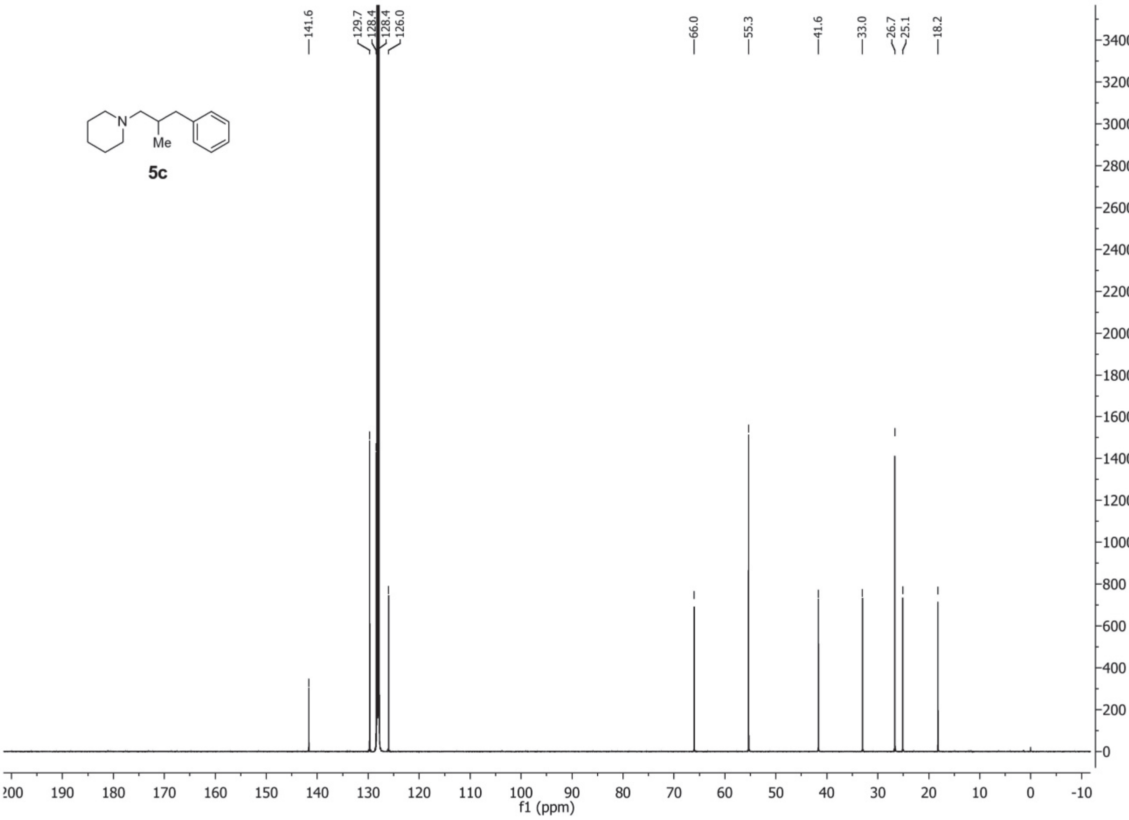
N
Me
5c
f1 (ppm)

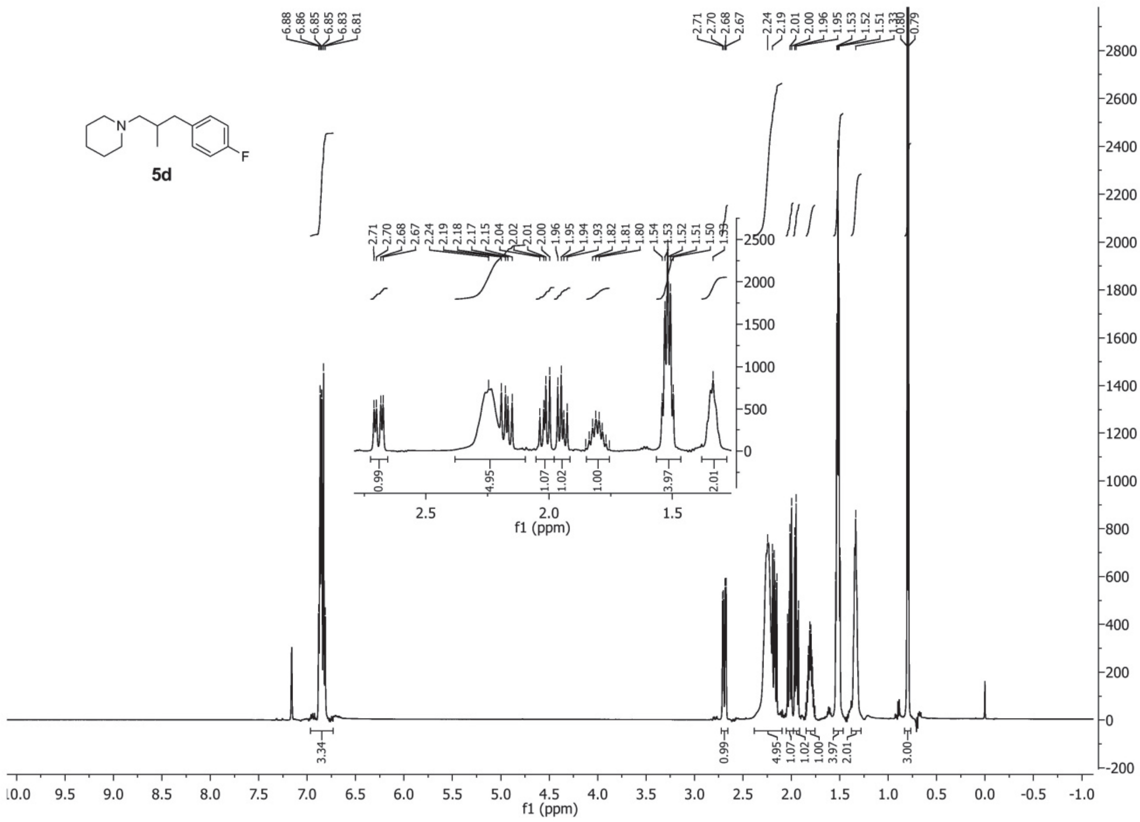

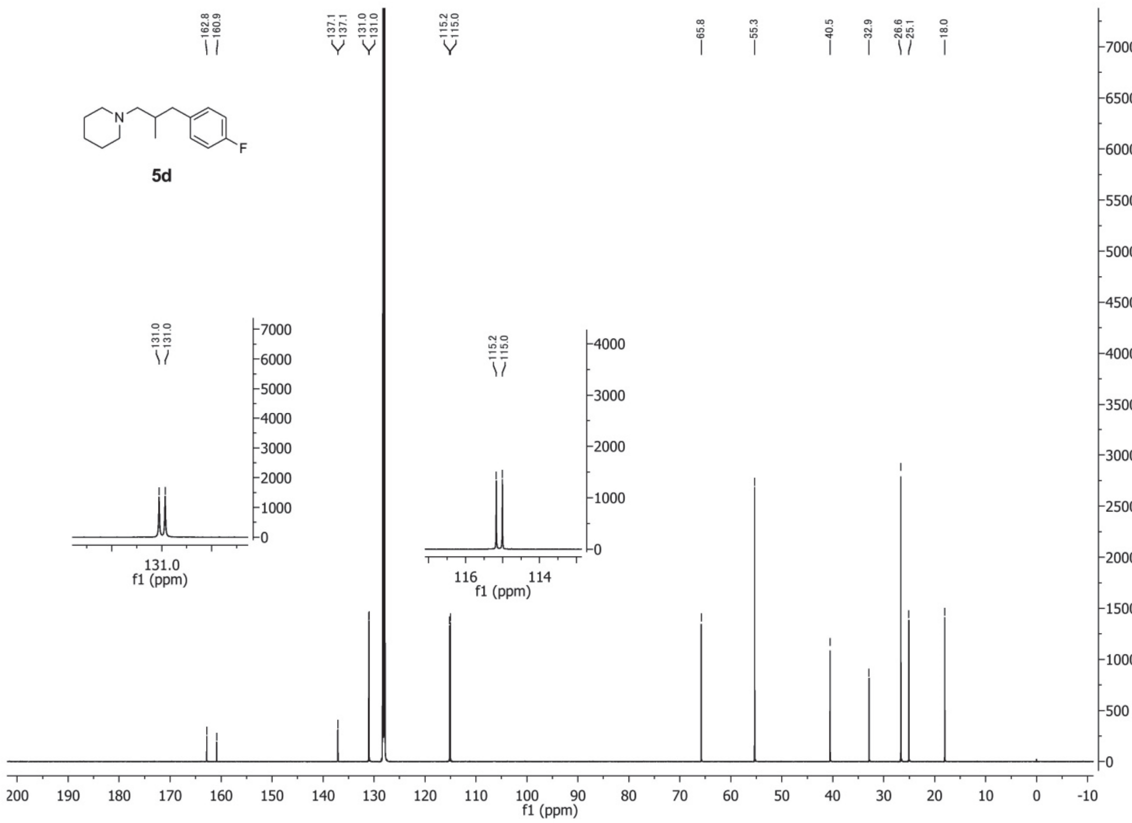

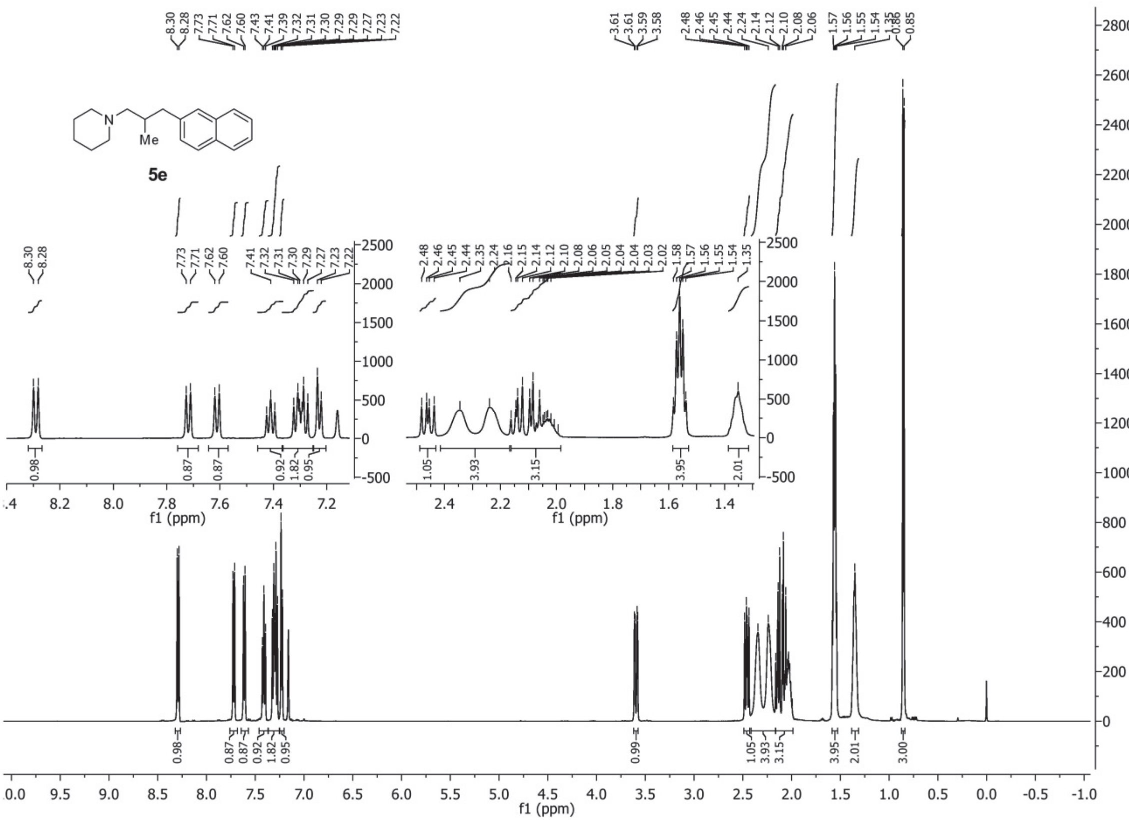
5e
f1 (ppm)

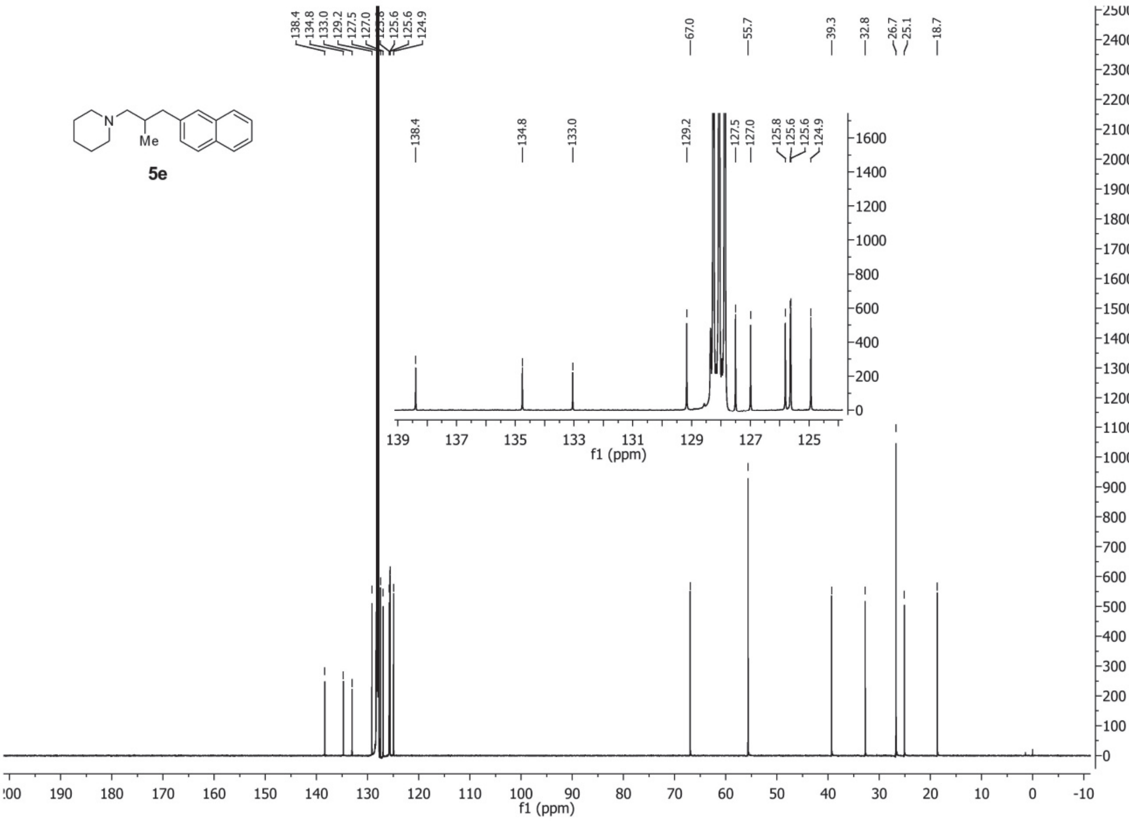
5e
f1 (ppm)

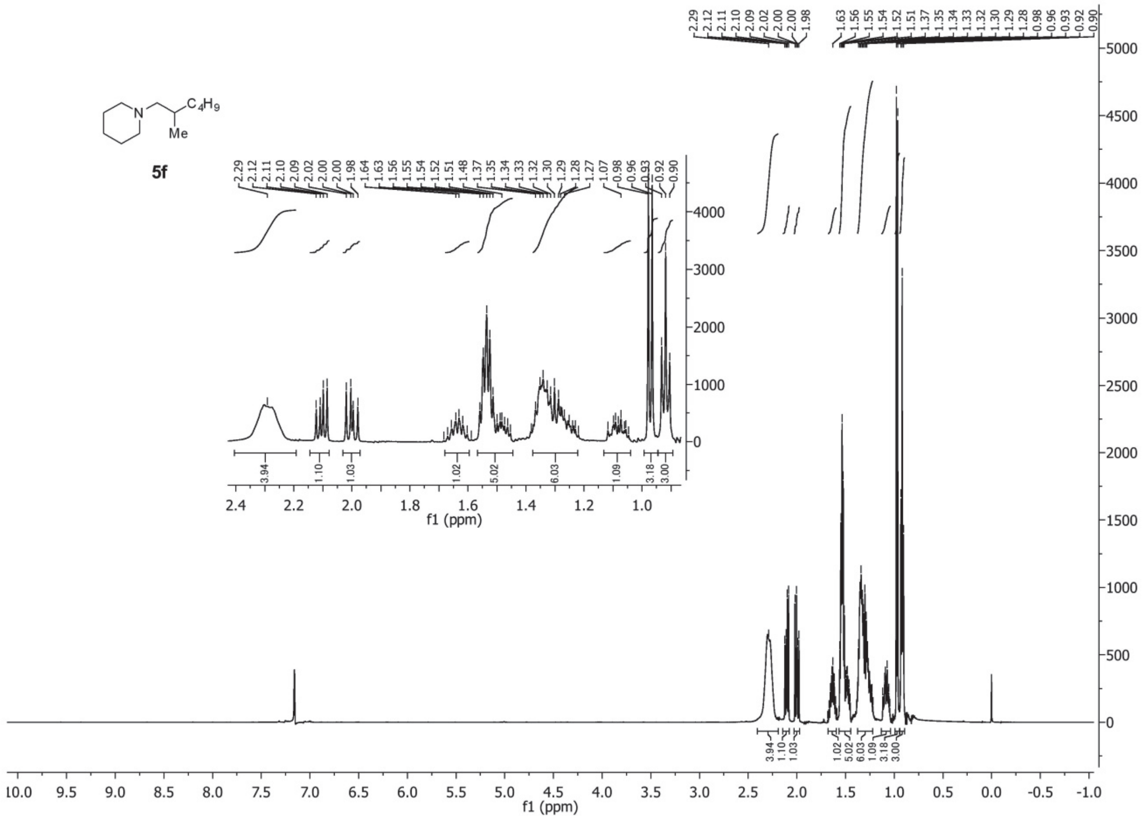
5f
N
C4H9
Me
2.29 2.12 2.11 2.10 2.09 2.02 2.00 2.00 1.98
1.63 1.56 1.55 1.54 1.52 1.51 1.37 1.35 1.34 1.33 1.32 1.30 1.29 1.28 0.98 0.96 0.93 0.92 0.90
3.94 1.10 1.03 1.02 5.02 6.03 1.09 3.18 3.00
f1 (ppm)

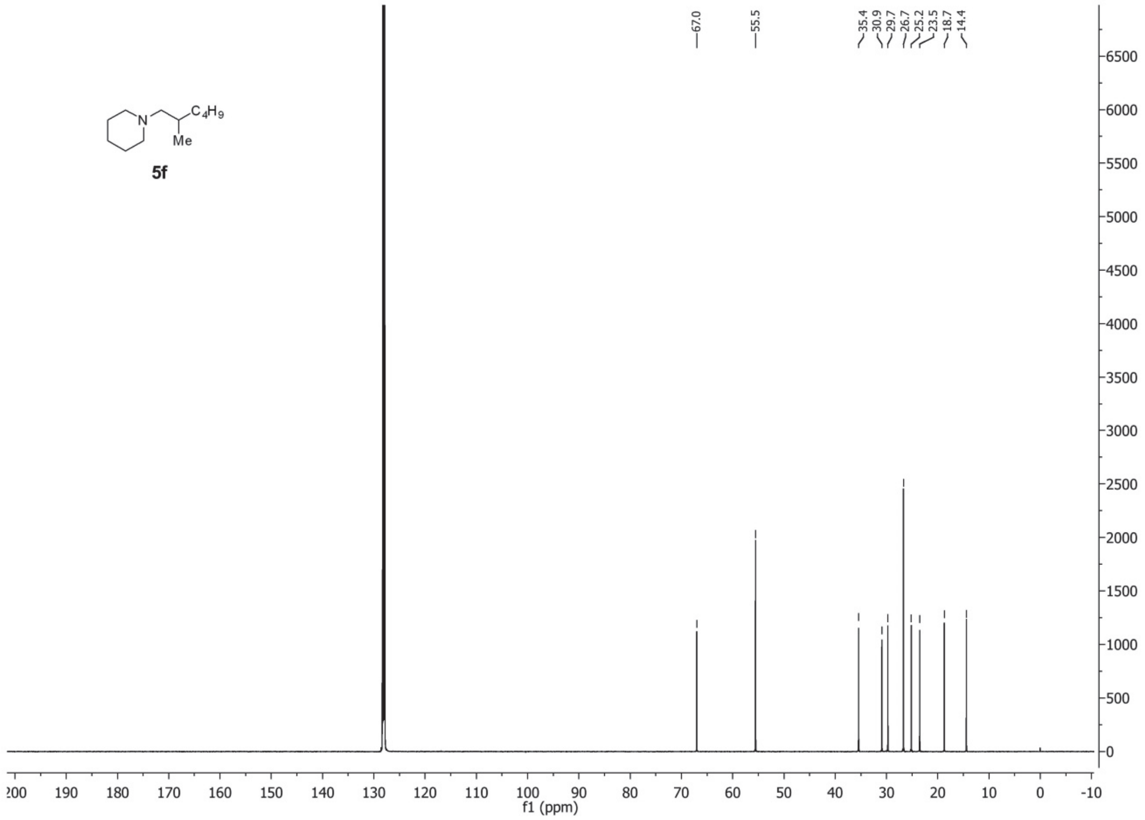
5f
N
C4H9
Me
67.0
55.5
35.4 30.9 29.7 26.7 25.2 23.5 18.7 14.4
f1 (ppm)

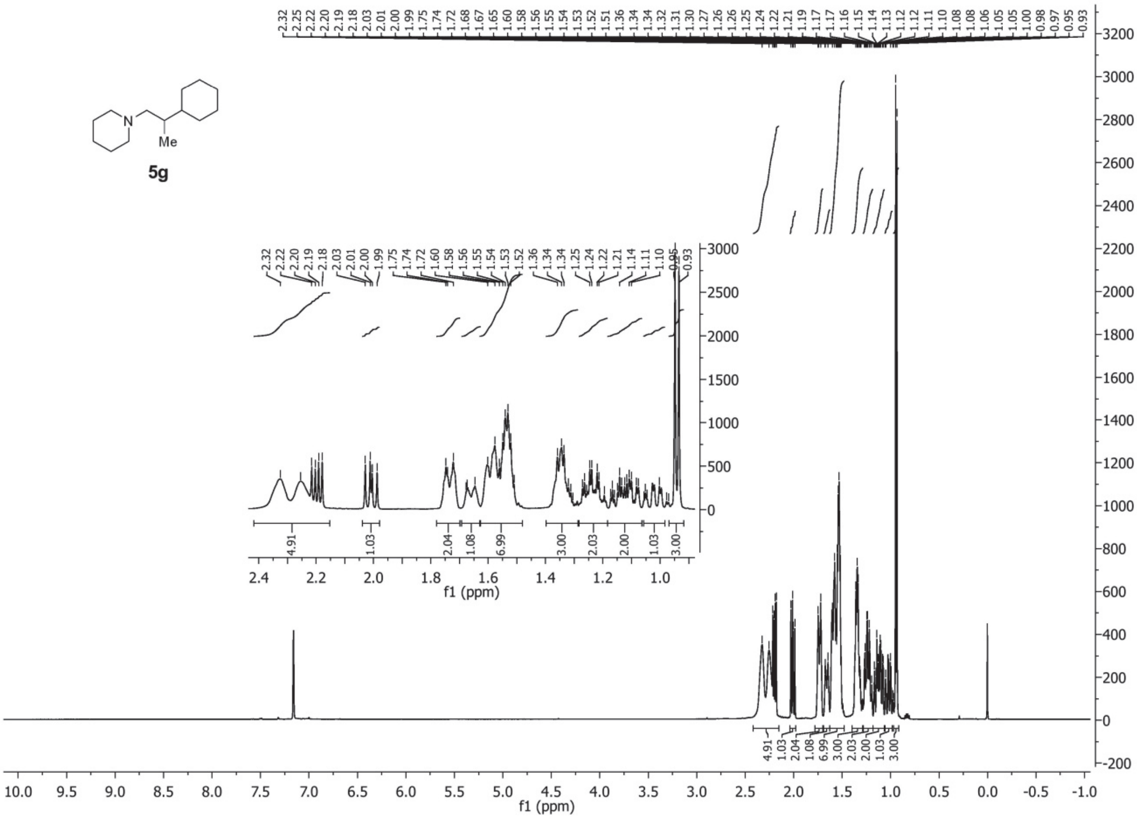
2.32 2.25 2.22 2.20 2.19 2.18 2.03 2.01 2.00 1.99 1.75 1.74 1.72 1.68 1.67 1.65 1.60 1.58 1.56 1.55 1.54 1.53 1.52 1.51 1.36 1.34 1.32 1.31 1.30 1.27 1.26 1.25 1.24 1.22 1.21 1.19 1.17 1.16 1.15 1.14 1.13 1.12 1.11 1.10 1.08 1.06 1.05 1.00 0.98 0.97 0.95 0.93
N
Me
5g
4.91
1.03
2.04
1.08
6.99
3.00
2.03
2.00
1.03
3.00
f1 (ppm)

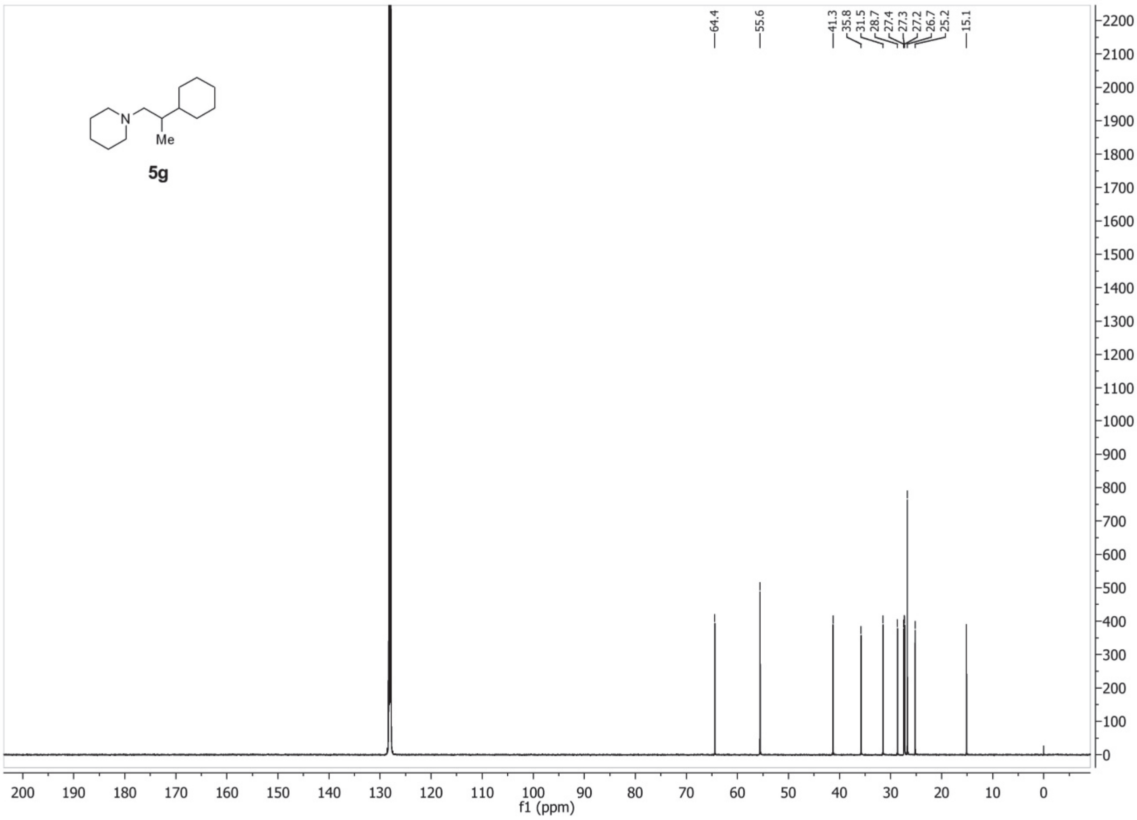
64.4
55.6
41.3
35.8
31.5
28.7
27.4
27.3
27.2
26.7
25.2
15.1
N
Me
5g
f1 (ppm)

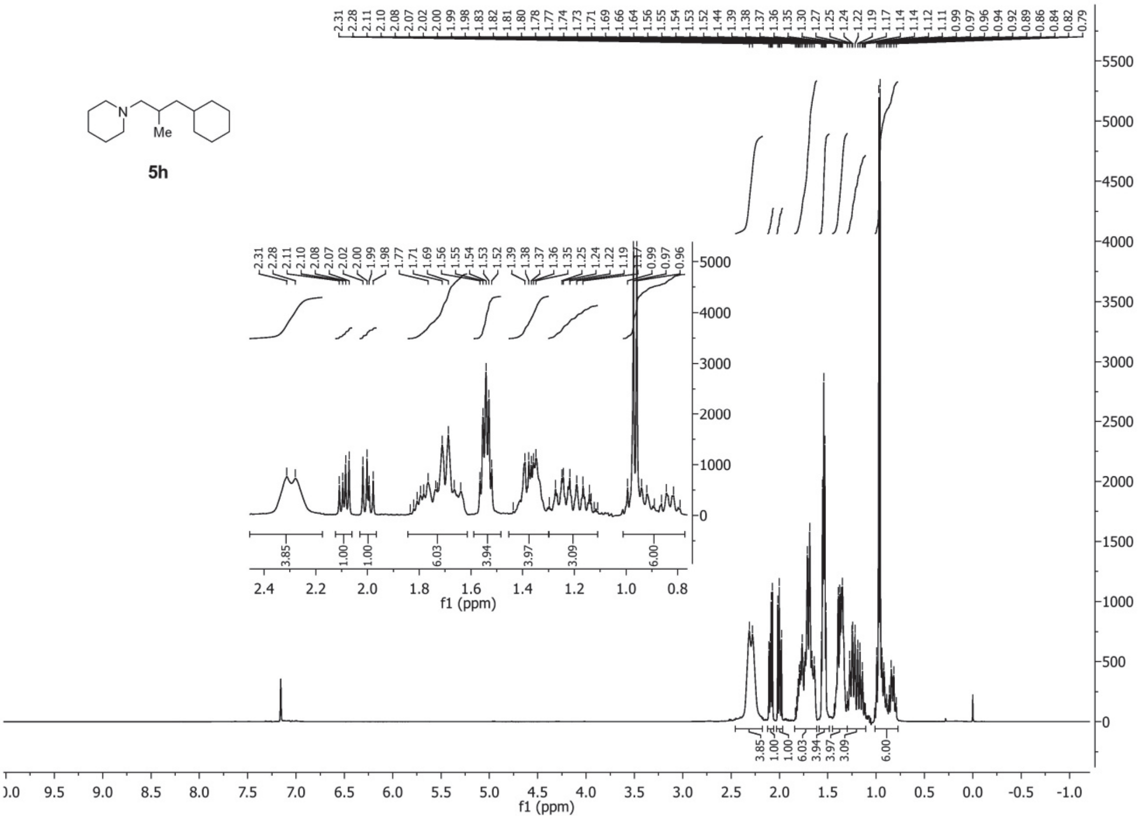
5h
Me
2.31 2.28 2.11 2.10 2.08 2.07 2.02 2.00 1.99 1.98 1.83 1.82 1.81 1.80 1.78 1.77 1.74 1.73 1.71 1.69 1.66 1.64 1.56 1.55 1.54 1.53 1.52 1.44 1.39 1.38 1.37 1.36 1.35 1.30 1.27 1.25 1.24 1.22 1.19 1.17 1.14 1.14 1.12 1.11 0.99 0.97 0.96 0.94 0.92 0.89 0.86 0.84 0.82 0.79
3.85 1.00 1.00 6.03 3.94 3.97 3.09 6.00
f1 (ppm)

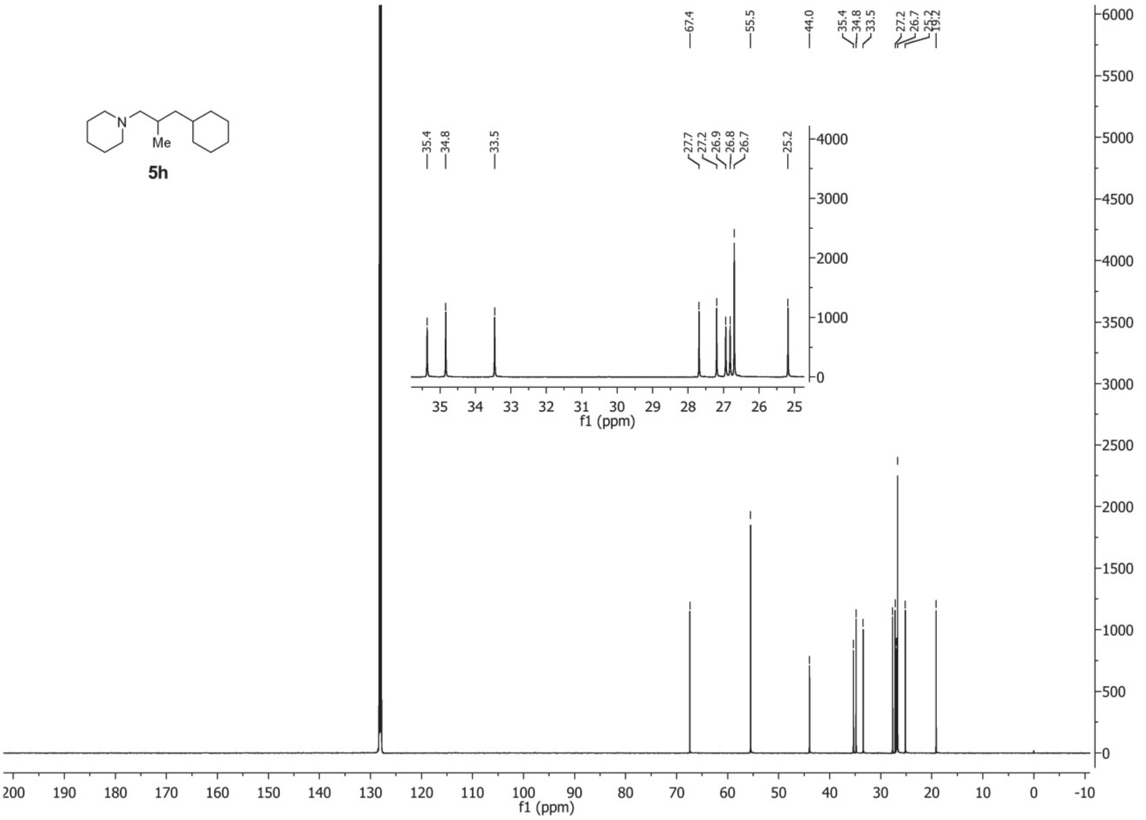
5h
Me
67.4 55.5 44.0 35.4 34.8 33.5 27.2 26.7 25.2 19.2
35.4 34.8 33.5 27.7 27.2 26.9 26.8 26.7 25.2
f1 (ppm)

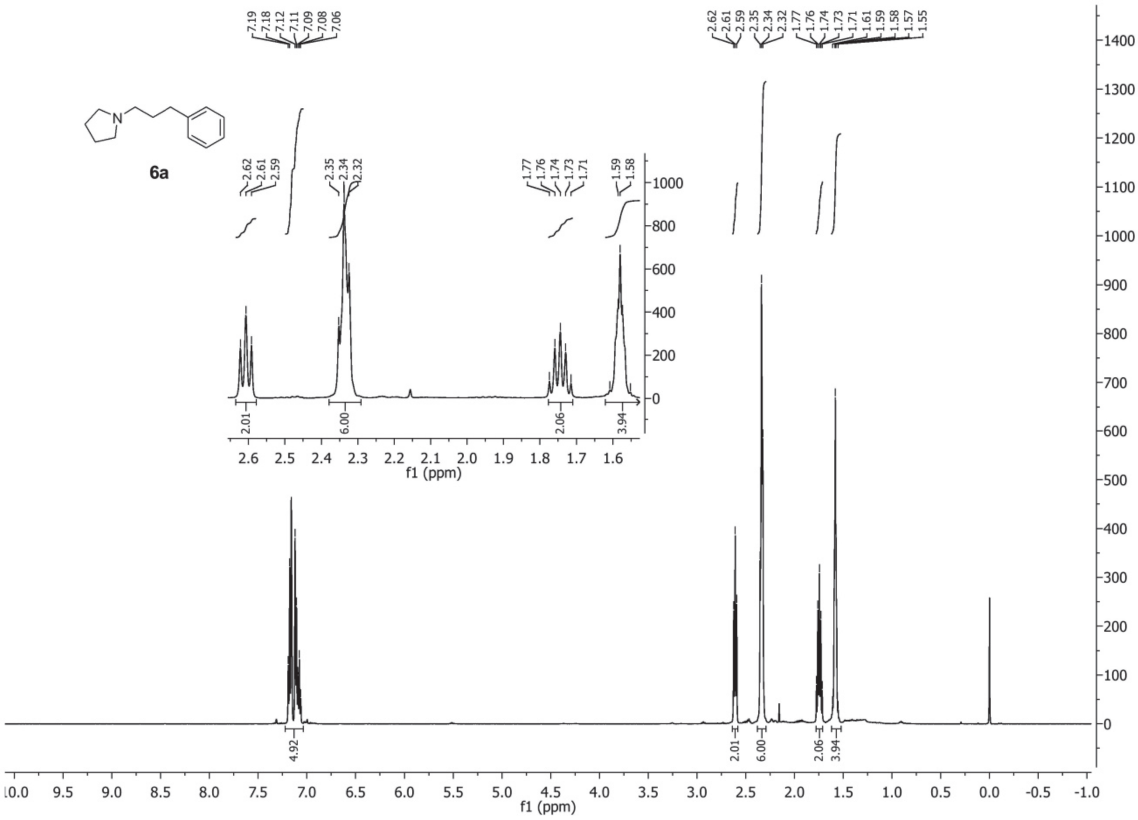
6a
7.19
7.18
7.12
7.11
7.09
7.08
7.06
2.62
2.61
2.59
2.35
2.34
2.32
1.77
1.76
1.74
1.73
1.71
1.61
1.59
1.58
1.57
1.55
4.92
2.01
6.00
2.06
3.94
f1 (ppm)

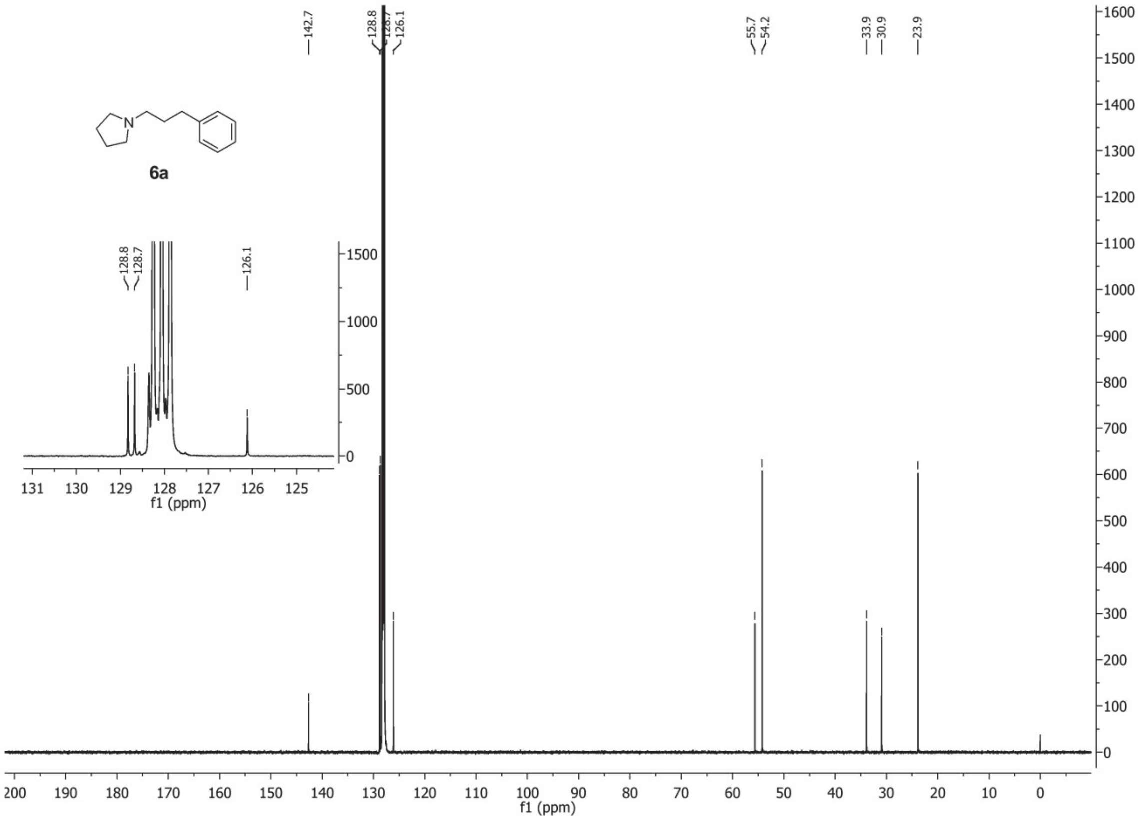
6a
142.7
128.8
128.7
126.1
55.7
54.2
33.9
30.9
23.9
f1 (ppm)
